# When Dicty Met Myco, a (Not So) Romantic Story about One Amoeba and Its Intracellular Pathogen

**DOI:** 10.3389/fcimb.2017.00529

**Published:** 2018-01-09

**Authors:** Elena Cardenal-Muñoz, Caroline Barisch, Louise H. Lefrançois, Ana T. López-Jiménez, Thierry Soldati

**Affiliations:** Department of Biochemistry, Sciences II, Faculty of Sciences, University of Geneva, Geneva, Switzerland

**Keywords:** *Dictyostelium discoideum*, *Mycobacterium marinum*, host-pathogen interactions, model organisms, phagocytosis, infection, methods

## Abstract

In recent years, *Dictyostelium discoideum* has become an important model organism to study the cell biology of professional phagocytes. This amoeba not only shares many molecular features with mammalian macrophages, but most of its fundamental signal transduction pathways are conserved in humans. The broad range of existing genetic and biochemical tools, together with its suitability for cell culture and live microscopy, make *D. discoideum* an ideal and versatile laboratory organism. In this review, we focus on the use of *D. discoideum* as a phagocyte model for the study of mycobacterial infections, in particular *Mycobacterium marinum*. We look in detail at the intracellular cycle of *M. marinum*, from its uptake by *D. discoideum* to its active or passive egress into the extracellular medium. In addition, we describe the molecular mechanisms that both the mycobacterial invader and the amoeboid host have developed to fight against each other, and compare and contrast with those developed by mammalian phagocytes. Finally, we introduce the methods and specific tools that have been used so far to monitor the *D. discoideum*—*M. marinum* interaction.

## Introduction

### The amoeba *Dictyostelium discoideum*

*Dictyostelium discoideum* is a soil amoeba that feeds on bacteria by phagocytosis. The Amoebozoa branch split from the common lineage leading to fungi and animals, shortly after these crown organisms split from the Plant lineage (Eichinger et al., 2005). *D. discoideum* has a very AT-rich (77.75%) haploid genome, organized into 6 chromosomes, which has been sequenced and annotated (dictyBase, 2004; Eichinger et al., 2005). The isolation in the 70's of axenic strains that could feed on liquid medium by pinocytosis (Watts and Ashworth, 1970) enabled the easy laboratory culture of *D. discoideum*. In addition, this amoeba is genetically tractable, being relatively easy to transform for gene expression from extrachromosomal plasmids, generation of knockins, and knockouts by homologous recombination (Veltman et al., 2009a,b; Wiegand et al., 2011; Mukai et al., 2016), and mutagenesis by Restriction Enzyme-Mediated Integration (REMI) (Kuspa, 2006). Moreover, *D. discoideum* can be used in multiple cell biology, biochemistry, and imaging assays, as well as for high-throughput screens and RNAseq analysis (Eichinger and Rivero, 2013; Kicka et al., 2014; Rosengarten et al., 2015). For these reasons, *D. discoideum* has been extensively used as a model system to study very diverse biological processes such as motility, chemotaxis, vesicular trafficking, gene expression, facultative multicellularity, and host-pathogen interactions (Barry and Bretscher, 2010; Maniak, 2011; Stevense et al., 2011; Loomis, 2014; Tosetti et al., 2014; Nichols et al., 2015).

#### Central role of autophagy in development and host-microbe interaction

In conditions of nutrient repletion, *D. discoideum* lives as a vegetative unicellular organism. However, when resources become scarce, the amoeba triggers a developmental program for the aggregation of hundreds of thousands of cells into a true multicellular organism (Raper, 1935; Dormann et al., 2000; Williams, 2006). This process is known as the “developmental cycle” of *D. discoideum* and has been extensively studied as one of the evolutionary origins of multicellularity. Aggregation and morphogenesis during development require a high level of cellular activity. To undertake this metabolic demand in the absence of nutrients, *D. discoideum* relays on macroautophagy (hereafter referred as autophagy), a major catabolic pathway in eukaryotes (Otto et al., 2004). Non-selective autophagy consists of the engulfment of bulk cytosolic material in a double-membrane compartment called the autophagosome. Upon fusion with lysosomes, autolysosomes degrade, and recycle their content, enabling cell survival under starvation. One of the major regulators of autophagy is the target of rapamycin complex 1 (TORC1). Under nutrient repletion, TORC1 downregulates autophagy by repressing the expression of autophagy genes and/or by phosphorylation and inhibition of proteins involved in autophagosome formation (reviewed in Nakatogawa, 2015). In *D. discoideum*, the TORC1 complex consists of the TOR serine/threonine kinase and two TOR activators, Lst8, and Raptor (Wullschleger et al., 2006; Rosel et al., 2012). Like in mammals, starvation decreases TORC1 activity, leading to induction of autophagy (King et al., 2011; Rosel et al., 2012).

Since many of the well-studied proteins known to participate in mammalian autophagy are conserved in this amoeba, autophagy can be easily monitored in *D. discoideum*. For instance, like in mammals, the activity of *D. discoideum* TORC1 can be analyzed by monitoring the phosphorylation state of Raptor and the TORC1 effector protein 4E-BP1 by immunoblotting (Rosel et al., 2012; Cardenal-Muñoz et al., 2017). In addition, in *D. discoideum* also the changes in autophagosome formation can be distinguished from changes in autophagic degradation by the differential response of Atg18 [WD-repeat protein interacting with phosphoinositides (WIPI) in mammals] and Atg8a (LC3/GABARAP family proteins in mammals) (Calvo-Garrido et al., 2010). Accordingly, during induction of autophagosome formation Atg8 and Atg18 translocate from the cytosol to membranes of nascent and elongating phagophores (the yet unclosed autophagic double membrane compartment). Immediately after closure of the autophagosome, Atg8 stays in both inner and outer membranes, while Atg18 dissociates. Upon fusion of the autophagosome with lysosomes, the hydrolytic enzymes delivered from the lysosome not only degrade the autophagic cargo but also the inner membrane of the autolysosome and its associated Atg8, while the Atg8 on the outside is recycled to the cytosol (reviewed in Dominguez-Martin et al., 2017).

In recent years, *D. discoideum* has also become an interesting model to study the molecular mechanisms regulating xenophagy (reviewed in Mesquita et al., 2016). Xenophagy is a selective autophagy pathway specifically recognizing and digesting intracellular pathogens. It relies on selective receptors that recruit the cargo to be degraded [i.e., bacteria or remnants of their phagosome decorated with “eat-me” signals such as ubiquitin (Ub) or galectins in mammals] to autophagic membranes (Thurston et al., 2009, 2016; Boyle and Randow, 2013; Noad et al., 2017). Several xenophagy receptors have been described in mammalian cells, but only one, p62, has been identified or studied so far in *D. discoideum* (Calvo-Garrido and Escalante, 2010; Gerstenmaier et al., 2015; Lampe et al., 2015; Cardenal-Muñoz et al., 2017). In addition, although *D. discoideum* lacks galectins, which recognize bacterial or host membrane glycans, another family of cytosolic lectins in this amoeba, the discoidins (Dsc), share many molecular and biological characteristics with galectins. Dsc are highly expressed upon starvation (Rosen et al., 1973), bind to self-glycans during development (Cooper and Barondes, 1984; Eitle et al., 1993) and can be secreted to the extracellular medium (Barondes et al., 1984). Interestingly, Dsc bind to sonicated extracts of *Escherichia coli* and *Klebsiella aerogenes* (Cooper et al., 1983), as well as to glutaraldehyde-fixed *K. aerogenes* bacteria (Madley and Hames, 1981). Recent studies have revealed that Dsc are enriched on compartments containing virulent *Legionella pneumophila* strains (i.e., Corby and JR32) but not the less virulent *L. hackelieae*, suggesting a role in innate immunity (Shevchuk et al., 2009; Urwyler et al., 2009). Moreover, one ortholog of the human TRIM37, a member of the Tripartite Motif protein superfamily (TRIMs) of autophagy receptors and regulators that often interact with galectins (Mandell et al., 2014; Kimura et al., 2015; Chauhan et al., 2016), has been identified in *D. discoideum* (Dunn et al., 2018). It will be interesting to uncover whether or not Dsc, TRIM-like protein and p62 cooperate in the immunity of *D. discoideum* against pathogenic bacteria.

### The bacterial pathogen *Mycobacterium marinum*

*Mycobacterium* is the only genus of the *Mycobacteriaceae* family. It comprises more than 150 different species, among them two of the most important human pathogens, *M. leprae* and *M. tuberculosis* (Mtb), causing leprosy and tuberculosis, respectively. The latter represents the most severe bacterial diseases, responsible for 1.8 million deaths worldwide in 2015 (World Health Organization, 2015, http://www.who.int/tb/publications/global_report). One of the important features of Mtb is its capacity to persist inside its host in a non-replicating state with low metabolic activity. This phenomenon is called dormancy (or latency) and affects 2–3 billion people (i.e., one third of the world population). Latent tuberculosis is non-transmissible and presents no clinical manifestation. However, the disease reactivates in 5–10% of the cases usually as a consequence of immunosuppression, becoming contagious. Therefore, providing treatments to prevent the reactivation of latent tuberculosis is an important concern in the control of this disease (World Health Organization, 2015).

Mtb belongs to the group of genetically related mycobacteria, the Mtb complex, that cause tuberculosis in human or other animals. However, another group of human pathogens emerging as a major public health problem is the Non-Tuberculous Mycobacteria (NTM) or Mycobacteria Other Than Tubercle bacilli (MOTT), which includes among others the species *M. ulcerans, M. abscessus, M. avium-intracellulare* complex (MAC) and *M. marinum* (Ryu et al., 2016). In contrast to Mtb, with no environmental reservoir demonstrated so far, *M. marinum* is an intracellular pathogenic mycobacterium with ubiquitous distribution. It can be found in aquatic environments, and it affects a wide range of freshwater and marine vertebrates including fish, amphibians, and turtles (Decostere et al., 2004). During infection of these animals, *M. marinum* forms granulomatous lesions highly similar to the ones produced by Mtb in humans. In addition, *M. marinum* also develops a latent disease in the zebrafish model (Parikka et al., 2012). Very importantly, and reflecting their genetic proximity, *M. marinum* is also an opportunistic pathogen of humans, but due to its optimal growth at 30°C, *M. marinum* infections are mostly localized and restricted to the skin and extremities. These lesions are usually painless and can be treated with anti-Mtb antibiotics when healing is not spontaneous (Aubry et al., 2017). At the cellular level, the infection course of *M. marinum* is also very similar to that of Mtb (reviewed in Ramakrishnan, 2004; Tobin and Ramakrishnan, 2008). Both bacteria avoid degradation within the host cells by arresting the maturation of their phagosomes and, contrary to the non-tuberculous *M. avium*, they escape to the host cytosol prior to dissemination (Stamm et al., 2003; Hagedorn et al., 2009; Simeone et al., 2015; Jamwal et al., 2016).

The genome of the *M. marinum* strain M was sequenced and annotated in 2008 by Stinear et al. (2008). Whole genome comparisons revealed that *M. marinum* is the ancestor of *M. ulcerans* and that both species are the closest relatives of the Mtb complex (Stinear et al., 2000, 2008). Among these species, *M. marinum* has the largest genome with 6.6 Mb, while the genome size of *M. ulcerans* and Mtb is 5.8 and 4.4 Mb, respectively. The genome of *M. marinum* shares 85% nucleotide identity with Mtb and 97% with *M. ulcerans*. Although phylogenetically and genetically close, Mtb and *M. marinum* present some differences such as the cell wall composition (Daffe et al., 1991; Tonjum et al., 1998), the abundance of proline-glutamic acid polymorphic guanine-cytosine-rich sequence (PE-PGRS) and proline-proline-glutamic acid (PPE) proteins (Cole et al., 1998; Bottai et al., 2012; Ates et al., 2015), and the synthesis of carotenoid pigments, which only occurs in *M. marinum* (Stinear et al., 2008).

Nevertheless, and more importantly, many virulence factors are conserved between *M. marinum* and Mtb. One example are the genes involved in dormancy (Dos-regulon) (Gerasimova et al., 2011). In addition, *M. marinum* and Mtb secrete virulence effector proteins through their complex cell walls thanks to very sophisticated secretion systems. In this context, the two mycobacteria possess five type VII secretion systems (T7SS) called ESX1-5 (reviewed in Groschel et al., 2016), in reference to the first secreted factor identified ESAT6 (Sorensen et al., 1995). The T7SS are not exclusive to mycobacteria and have been found in other bacterial genera such as *Streptomyces* and *Bacillus* (Gey Van Pittius et al., 2001; Pallen, 2002; Unnikrishnan et al., 2017). Despite their diversity, the T7SS share two common characteristics: the presence of FtsK/SpoIIIE transmembrane proteins and the secretion of small peptides of around 100 amino acids with the conserved motif Trp-X-Gly (WXG) (Pallen, 2002). The WXG motif is involved in the generation of a helix-turn-helix structure and is present in two of the most studied T7SS secreted peptides, the 6 kDa early secretory antigenic target ESAT6 and the 10 kDa culture filtrate protein CFP10 (Renshaw et al., 2005). Each ESX system is encoded in its respective ESX cluster or locus, which also contains the genes for secreted peptides and other accessory proteins (Bitter et al., 2009). The ESX loci do not complement each other (reviewed in Abdallah et al., 2007), which suggests that they are involved in different functions. Only three of them have been shown to play a role in pathogenesis: ESX-1, 3, and 5. Whereas ESX-3 is required for iron and zinc acquisition (Serafini et al., 2009, 2013; Siegrist et al., 2009, 2014), ESX-5 is involved in nutrient intake (Ates et al., 2015), cell wall integrity and secretion of PE and PPE proteins. The ESX-1 locus is the most studied, since it was shown to be very important for mycobacterial virulence (Pym et al., 2002). The attenuated *M. microti* and *M. bovis* BCG bacille Calmette–Guérin (BCG), used for vaccines, lack a partially overlapping region of the ESX-1 locus, which was called Region of Difference 1 (RD1). This region comprises two genes that encode the above mentioned secreted peptides ESAT6 and CFP10. Early studies using transposon libraries and purified proteins identified ESX-1, and in particular ESAT6 and CFP10, as membranolytic factors in both Mtb and *M. marinum* (Hsu et al., 2003; Gao et al., 2004). Importantly, the deletion of *M. marinum* RD1 can be complemented with homologous genes from Mtb, thus confirming the functional similarity of the RD1 regions in both species (Gao et al., 2004). Even though a 3.7 kb portion of *M. marinum* RD1 is partially duplicated within its genome (Stinear et al., 2008), deletion of RD1 prevents *M. marinum* escape from its phagosome as occurs with Mtb, suggesting that the duplicated fragment of *M. marinum* is not functional (Simeone et al., 2015; Cardenal-Muñoz et al., 2017).

These pathologic, phylogenetic, and genomic aspects make of *M. marinum* an interesting model to study mycobacterial infections. Importantly, *M. marinum* also has advantages for laboratory work. Despite the fact that this species is considered a slow growing mycobacterium, *M. marinum* grows faster than Mtb, with a doubling time of 6–8 h vs. the 20–24 h of Mtb. Furthermore, *M marinum* is a level 2 biosafety organism, and numerous molecular tools are available and transposable from Mtb to *M. marinum*, and *vice versa*.

### The *D. discoideum*—*M. marinum* infection model

*D. discoideum* has been used to study conserved mechanisms of bacterial pathogenicity and host defenses involved in many human diseases induced by diverse bacteria genera such as *Francisella, Legionella, Salmonella*, or *Mycobacterium* (Bozzaro and Eichinger, 2011; Weber and Hilbi, 2014; Brenz et al., 2017; Cardenal-Muñoz et al., 2017; Steiner et al., 2017). In both *D. discoideum* and human phagocytes, the main cell-autonomous defenses rely on the highly conserved microbicidal machinery that arms the phagosome (Boulais et al., 2010). This includes a number of genes and pathways involved in bacteria recognition, phagocytic uptake, actin dynamics, phagosome identity, acidification and maturation, lysosomal degradation, production of Reactive Oxygen Species (ROS), metal poisoning and nutrition immunity (recently reviewed in Dunn et al., 2018).

The ability of *M. marinum* to replicate within *D. discoideum* was first described in 2003 (Solomon et al., 2003). Solomon and collaborators monitored monolayers of *D. discoideum* infected with GFP-producing *M. marinum* at 25.5°C, a temperature optimal for the joint growth of host and bacterium. For these long-term infection assays, and to avoid rapid host cell death, *D. discoideum* needs to be infected at low multiplicity of infection (MOI ≤1–10). After an initial incubation without antibiotics, to allow the phagocytosis of *M. marinum*, streptomycin was added to the infection sample to impede the growth of extracellular bacteria. In this first approach, the increased growth of *M. marinum* was assessed by direct observation, but other quantitative methods such as luminescence recording, fluorescence-activated cell sorting (FACS) and colony-forming units (CFUs) counting have confirmed the intracellular proliferation of this bacterium within *D. discoideum*, both in static and shaking cultures (Solomon et al., 2003; Hagedorn and Soldati, 2007; Arafah et al., 2013). Other antibiotics such as amikacin or a mix of penicillin and streptomycin can be used to kill the extracellular *M. marinum* when performing infection assays in *D. discoideum* (Arafah et al., 2013). The presence of extracellular bacteria in the infection sample can be tested by incubating live cells with an anti-*M. marinum* serum (anti-mar) and performing immunofluorescence assays (Supplementary Table 1); Only the extracellular bacteria, and not those inside the amoeba, are accessible to the serum (Hagedorn et al., 2009).

## *M. marinum* infection course in *D. discoideum*

**Figure 1:**
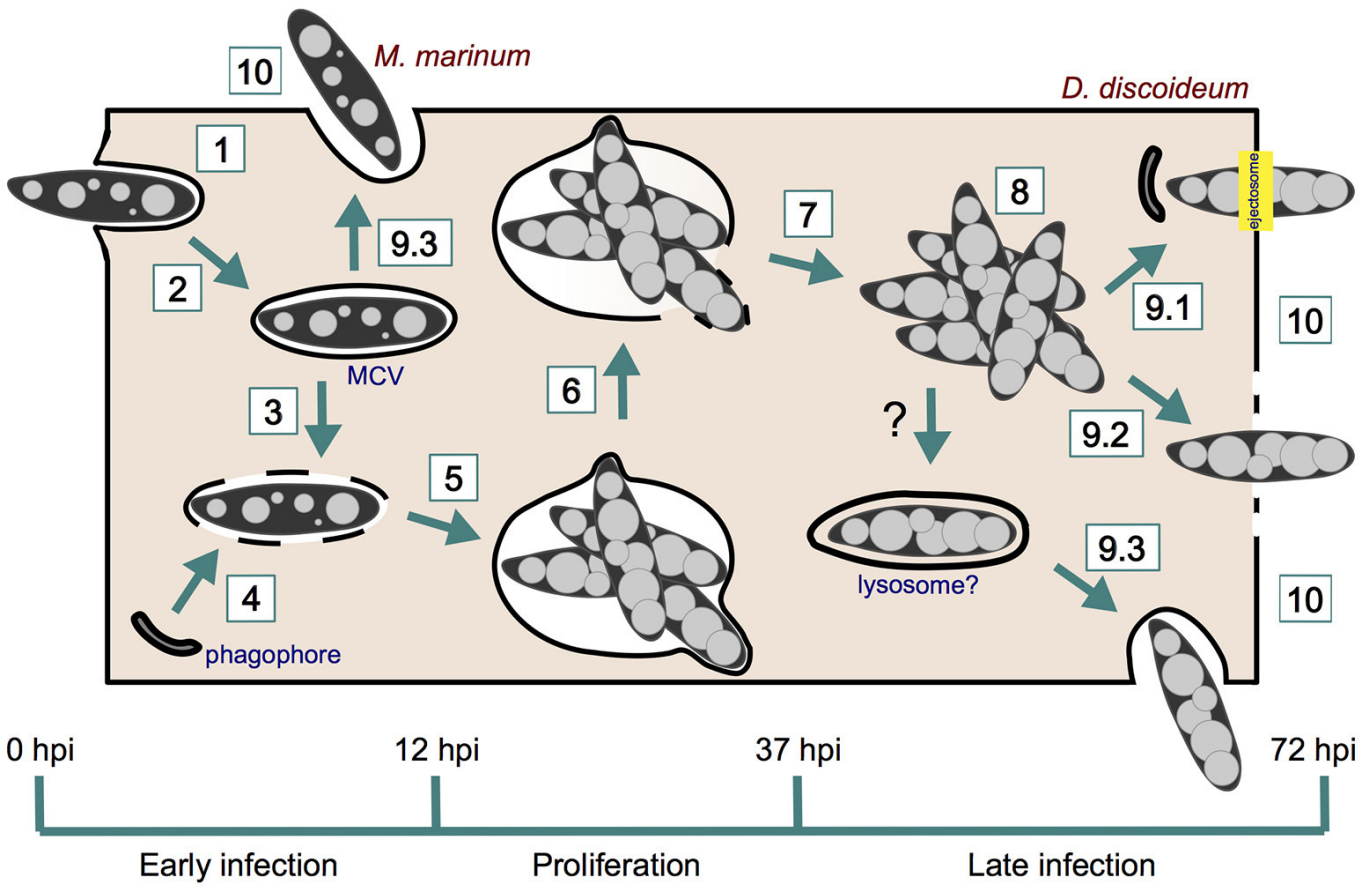
*M. marinum* infection course in *D. discoideum*. *M. marinum* is phagocytosed by *D. discoideum* (1) and rapidly manipulates its phagocytic pathway to reside within a replicative niche (2). The ESX-1 secretion system of *M. marinum* perforates the MCV (3), which induces the recruitment of phagophores for membrane repair (4). *M. marinum* proliferates within its MCV (5), which finally breaks (6) and release mycobacteria to *D. discoideum* cytosol (7). Bacteria continue growing in the host cytosol (8) prior to egress by ejection (9.1), lytic death (9.2), or exocytosis (9.3). Phagophores are also recruited to the site of ejection for plasma membrane repair. Recapture into lysosome-like compartments may precede late exocytosis. However, this has not been shown yet in *D. discoideum*. Early exocytosis (9.3) can be induced upon starvation. Intercellular dissemination occurs after *M. marinum* release from the amoeba (10).

The infection cycle of *M. marinum* in *D. discoideum* starts with the phagocytosis of the bacterium by the amoeba, and finishes with the bacterial egress from the cell (Figure 1 and Table 1). Three different growth stages of *M. marinum* can be distinguished during the infection cycle: (i) an initial phase during the first 12 h post-infection (hpi), when *M. marinum* actively manipulates the *D. discoideum* phagocytic pathway to convert the phagosome where it resides into a replication-permissive niche [mycobacteria-containing vacuole (MCV)], (ii) an enhanced proliferation phase between 12 and 37 hpi, and (iii) a phase of arrested proliferation and/or decreased bacterial load due to bacterial death or release after 37 hpi (Hagedorn and Soldati, 2007). This infection cycle might be impacted by the number of bacteria initially taken up. When a single amoeba internalizes a clump of bacteria, the *M. marinum* clump either cannot initiate its intracellular growth, remaining in a dormant or undigested state as occurs in zebrafish, or causes cytotoxicity to *D. discoideum* (Solomon et al., 2003; Parikka et al., 2012).

**Table 1:** *M. marinum* infection stages in *D. discoideum* and methods used for their analysis.

**Infection stage**	**Method used for analysis**	***D. discoideum*** **strains/markers**		***M. marinum*** **strains/markers**	
1. Phagocytosis	FACS (Sattler et al., 2013)	wt	–	wt	pMSP12::GFP
	IFA (Hagedorn et al., 2009)	wt	F-actin (phalloidin)	wt	pMSP12::GFP
	InfectChip (Delince et al., 2016)	wt	–	wt	pCherry10
	Live microscopy (Hagedorn et al., 2009)	wt	GFP-ABD	wt	pR2Hyg
	TEM (Hagedorn et al., 2009)	wt	–	wt	–
2. Niche establishment	IFA (Hagedorn and Soldati, 2007; Kolonko et al., 2014)	wt, *wshA*-	ArpC4-GFP, calmodulin, cathepsin D, Coronin-GFP, F-actin, p80, vacuolins, VMC, VatA, GFP-WASH	wt, L1D	pCherry3, pMSP12::GFP
	Live microscopy (Kolonko et al., 2014; Barisch et al., 2015a,b; Gerstenmaier et al., 2015; Barisch and Soldati, 2017b; Cardenal-Muñoz et al., 2017)	wt	ABD-GFP, AmtA-mCherry/GFP, DQ Green BSA, LysoSensor Green, NR, GFP-Rab5a, GFP-Rab7a, GFP-Rab11c, TRITC-dextran, VacA-GFP, VatB-RFP, VatM-GFP	wt, ΔRD1	pCherry3/10, pMSP12::DsRed/GFP, Vibrant DyeCycle Ruby
	TEM (Cardenal-Muñoz et al., 2017)	wt	–	wt	–
3. MCV membrane damage	IFA (Cardenal-Muñoz et al., 2017)	wt, *atg1*-	Atg8a, GFP-p62, Ub (FK2)	wt, ΔCE	pCherry10
	Immunoblot (Cardenal-Muñoz et al., 2017)	wt	p-4E-BP1 (T70), Abp1, p-Raptor (S863)	wt, ΔRD1	pCherry10, pMSP12::DsRed
	Live microscopy (Cardenal-Muñoz et al., 2017)	wt, *atg1*-	GFP-Atg8a, GFP-Atg18, DQ Green BSA, GFP-Ub	wt, ΔRD1, ΔRD1::RD1-2F9	pCherry10, pMSP12::DsRed, Vibrant DyeCycle Ruby
	qPCR (Cardenal-Muñoz et al., 2017)	wt	*atg1, atg8a, atg8b, p62*	wt, ΔRD1	–
4. MCV membrane repair	IFA (Cardenal-Muñoz et al., 2017)	wt, *atg1*-, *atg1*- Atg1-GFP, *atg8*-	Ub (FK2)	wt	pCherry10
	Immunoblot (Cardenal-Muñoz et al., 2017)	wt	p-4E-BP1 (T70), Abp1, p-Raptor (S863)	wt, ΔRD1	pCherry10, pMSP12::DsRed
	Live microscopy (Cardenal-Muñoz et al., 2017)	wt	GFP-Lamtor1, GFP-Lst8, GFP-Raptor, GFP-Rheb	wt, ΔRD1	pCherry10, pMSP12::DsRed
	TEM (Cardenal-Muñoz et al., 2017)	wt, *atg1*-	–	wt	–
5. Bacteria replication within the MCV	FACS (Hagedorn and Soldati, 2007; Hagedorn et al., 2009; Lelong et al., 2011; Kolonko et al., 2014)	wt, *kil2*-, *racH*-, *vacB*-, *wshA*-	MB38::ESAT-6	wt, L1D, ΔRD1	pMSP12::GFP
	IFA (Solomon et al., 2003; Hagedorn and Soldati, 2007; Lelong et al., 2011)	wt, *kil2*-, *racH*-, *vacB*-	vacuolins, coronin-GFP, p80	wt, L1D	*map24*::GFP, pMSP12::GFP
	Luminescence recording in microplate reader (Ouertatani-Sakouhi et al., 2017)	wt	–	wt	pMV306::*lux*
	TEM (Hagedorn et al., 2009)	wt	–	wt	–
6. MCV rupture	IFA (Hagedorn and Soldati, 2007; Hagedorn et al., 2009; Lelong et al., 2011; Cardenal-Muñoz et al., 2017)	wt, *kil2*-, *racH*-, *vacA*-, *vacB*-	MB38::ESAT-6, p80, Ub (KF2), vacuolins	wt, ΔRD1	pCherry10, pMSP12::GFP
	Live microscopy (Barisch et al., 2015b)	wt	VacA-GFP	wt	pCherry10
	TEM (Hagedorn et al., 2009)	wt, *racH*-	–	wt, ΔRD1	–
7. Escape from the MCV	IFA (Hagedorn and Soldati, 2007; Hagedorn et al., 2009; Gerstenmaier et al., 2015; Cardenal-Muñoz et al., 2017)	wt, *racH*-, *vacA*- *vacB*-	Atg8a, F-actin (phalloidin), MB38::ESAT-6, GFP-p62/Sqstm1, p80, Ub (FK2), vacuolins	wt, ΔRD1	pCherry10, pMSP12::GFP
	Live microscopy (Barisch et al., 2015a,b; Barisch and Soldati, 2017b)	wt	AmtA-mCherry/GFP, RFP/GFP-Plin, VacA-GFP	wt, ΔRD1	pCherry10
	TEM (Hagedorn et al., 2009)	wt, *racH*-	–	wt, ΔRD1	–
8. Cytosolic replication	Luminescence recording in microplate reader (Cardenal-Muñoz et al., 2017)	wt, *atg1*-, *atg8a*-, *p62*-	–	wt, ΔRD1	pMV306::*lux*
9.1. Egress by ejection	CLEM (Gerstenmaier et al., 2015)	wt	F-actin (Lifeact-GFP)	wt	pCherry3
	FACS (Gerstenmaier et al., 2015)	wt, *atg1*-	PI	wt	pMSP12::GFP
	IFA (Hagedorn et al., 2009; Kolonko et al., 2014; Gerstenmaier et al., 2015)	wt, *atg1*-, *atg5*-, *atg6*-, *atg7*-, *p62*/*sqstm1*-, *racH*-, *vacA*-, *vacB*-, *wshA*-, *wshA*- GFP-WASH	GFP-2xFYVE, Arp3, Atg8a, GFP-Atg18, Coronin, F-actin (phalloidin), GFP, MB38::ESAT-6, myoII, myoB, GFP-p62/Sqstm1, p80, PM4C4, Ub (FK2)	wt, ΔRD1	anti-mar, pCherry3, pMSP12::GFP, pR2Hyg
	Live microscopy (Hagedorn et al., 2009; Gerstenmaier et al., 2015)	wt	ABD-GFP, GFP-ABD, DAPI, TRITC-dextran	wt	pMSP12::GFP, pR2Hyg
	TEM (Gerstenmaier et al., 2015)	wt, *atg1*-	–	wt	–
9.2. Egress by host cell death	FACS (Gerstenmaier et al., 2015)	wt, *atg1*-	PI	wt	pMSP12::GFP
	InfectChip (Delince et al., 2016)	wt	–	wt	pCherry10
9.3. Exocytosis	IFA (Hagedorn et al., 2009)	wt	GFP-ABD, F-actin (phalloidin), p80, PM4C4, vacuolin	wt	pMSP12::GFP
	InfectChip (Delince et al., 2016)	wt	–	wt	pCherry10
	Live microscopy (Gerstenmaier et al., 2015)	wt	ABD-GFP, TRITC-dextran	wt	pMSP12::GFP
	SEM (Hagedorn et al., 2009)	wt	–	wt	–
	TEM (Hagedorn et al., 2009)	wt	–	wt	–
10. Intercellular dissemination	Dissemination/Transmission assay [fixed fluorescence microscopy (Hagedorn et al., 2009) or FACS (Gerstenmaier et al., 2015)]	wt, *atg1*-, *racH*-	GFP-ABD, Lifeact-RFP	wt	pMSP12::GFP, pR2Hyg
	IFA (Hagedorn et al., 2009)	wt	F-actin (phalloidin), p80	wt	pMSP12::GFP

### Early *M. marinum* infection phase (~0–12 hpi)

**Figure 2:**
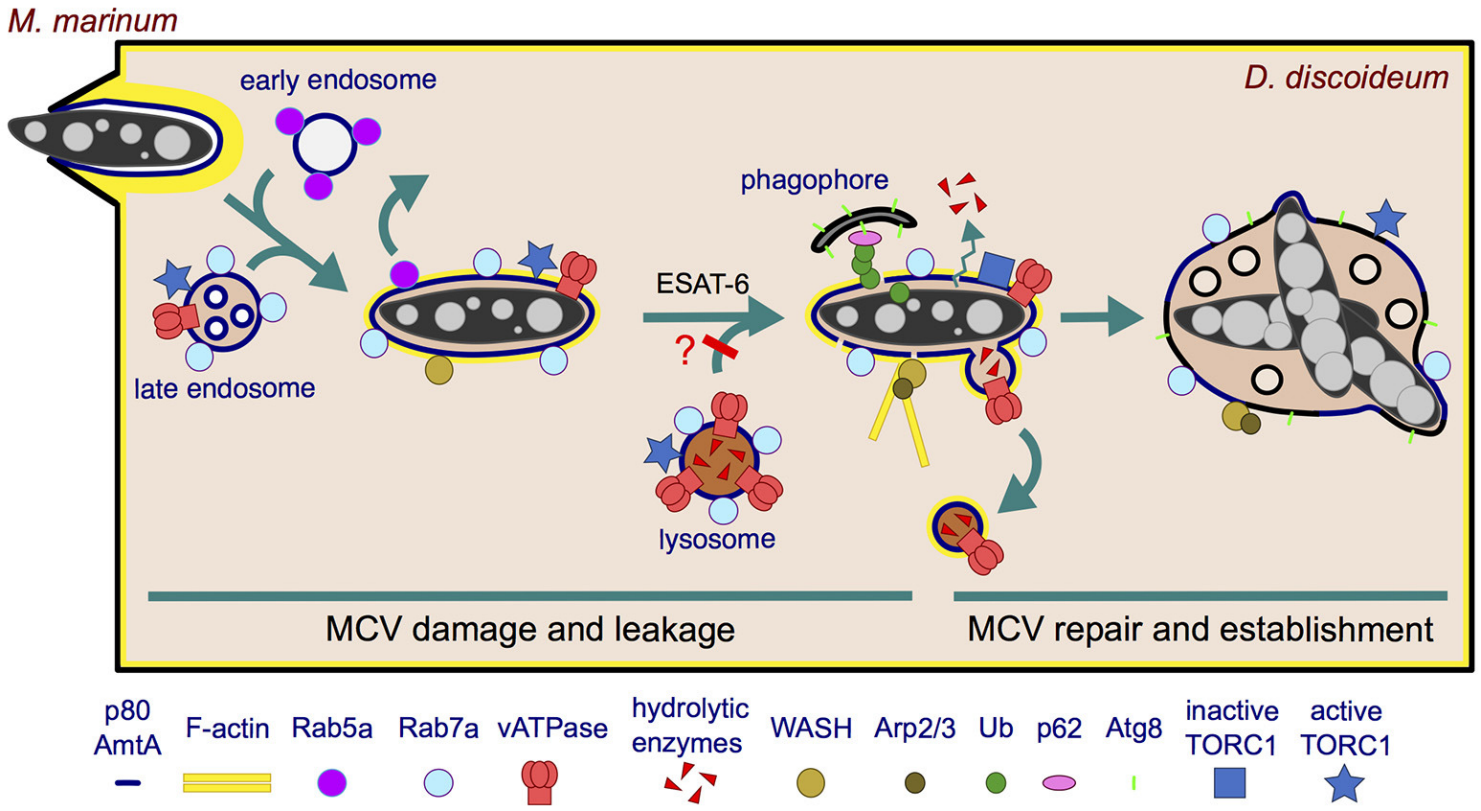
Establishment of the MCV during the first 12 h of infection. *M. marinum* is engulfed by an F-actin-positive phagocytic cup, resulting in an early phagosome that likely fuses sequentially with early and late endosomes. Rab5a is rapidly recycled from the MCV, which transiently acquires characteristics of a late endosome (Rab7a^+^, vATPase^+^, active TORC1^+^) but does not accumulate hydrolases. The undetectable level of hydrolases might result from defects in delivery, efficient recycling or leakage out of the MCV. In the case lysosomes do fuse with the MCV and deliver hydrolases, these might be retrieved by a mechanism dependent on WASH and Arp2/3 complexes. These complexes induce actin polymerization at the MCV, contributing to the retrieval of the vATPase and possibly hydrolases. In addition, *M. marinum* secretes ESAT-6 that damages the membrane, which renders the MCV leaky for ions and possibly lysosomal enzymes. Moreover, present evidence does not exclude the possibility that lysosomal fusion with the MCV is blocked. The membrane perforations induce an autophagic response in *D. discoideum*: TORC1 is inactivated and induces transcription of autophagy genes and formation of phagophores. The phagophores are recruited to the damaged MCV to repair the membrane and possibly deliver nutrients. *M. marinum* consequently survives and proliferates within a repaired and permissive MCV.

During uptake, *M. marinum* is surrounded by a circular ruffle and ingested into a newly produced phagosome (Figure 2). The engulfment of the bacterium by the tight phagocytic cup has been observed by microscopy of live and fixed cells, using markers of the actin cytoskeleton of *D. discoideum* (Hagedorn et al., 2009). In addition, phagocytosis of GFP-producing or Alexa 488 hydrazide-labeled *M. marinum* has been quantitatively monitored by flow cytometry (Sattler et al., 2013). It is important to notice that the labeling of surface amines with Alexa Fluor 488-tetrafluorophenyl ester (Alexa Fluor 488-TFP), normally used for other bacteria such as *Klebsiella pneumoniae* or *Salmonella enterica*, is not suitable for *M. marinum*, presumably due to its extremely hydrophobic and poor-in-proteins cell wall (Sattler et al., 2013).

Ten minutes after phagocytosis, the early endosomal marker Rab5a is withdrawn from the *M. marinum* phagosome, compared to 3 min for the non-pathogenic *M. smegmatis* (Barisch et al., 2015a). Subsequently, the late endosome and lysosome marker Rab7a is detected at the early MCV (Cardenal-Muñoz et al., 2017), as occurs in macrophages (Lerena and Colombo, 2011). However, *M. marinum* rapidly bypasses the phagocytic pathway and blocks the maturation of its compartment. Thus, late maturation markers such as vacuolin and cathepsin D are absent from the MCV from 0.3 to 6 hpi, and the vacuolar H^+^-ATPase (vATPase) is almost undetectable on the MCV at the end of this infection phase (12 hpi, Figure 2) (Hagedorn and Soldati, 2007). In this context, and contrary to *M. smegmatis*, only a small percentage of the phagocytosed *M. marinum* are killed by *D. discoideum* within the first hours (Hagedorn and Soldati, 2007), even in conditions of mild starvation that stimulate autophagy (i.e., phosphate buffer supplemented with 5% nutrient-rich medium) (Delince et al., 2016).

It has been shown that actin polymerization is required for the efficient maturation arrest of the *M. marinum* MCV during early infection of *D. discoideum* (Kolonko et al., 2014). Thus, Arp2/3 and WASH (Wiskott-Aldrich syndrome protein and Scar Homolog), two complexes involved in actin nucleation and polymerization on endosomal membranes (Duleh and Welch, 2010), can be observed in increasing association with the MCV from 0 to 6 hpi (Figure 2). This is concomitant with the presence of a patchy F-actin coat covering the MCV and with the absence of VatA, a peripheral subunit of the vATPase, on the same MCV at 6 hpi (Kolonko et al., 2014). On the contrary, amoebae either treated with the inhibitor of actin polymerization latrunculin A (LatA), producing a fusion construct between VacA and the actin-depolymerizing factor cofilin (VMC), or lacking the WASH complex (*wshA*-) harbor actin-negative MCVs which accumulate VatA and VatM (a transmembrane subunit of the vATPase), while losing the endocytic marker p80. Interestingly, p80 is not lost from the phagosome containing *M. smegmatis* and the avirulent mutant *M. marinum* L1D (Kolonko et al., 2014) (Supplementary Table 2). In agreement with the LatA-dependent accumulation of the vATPase on MCVs, LatA treatment also leads to acidification of the bacterial compartment, resulting in decreased bacteria viability (Kolonko et al., 2014). Interestingly, actin polymerization also prevents the acidification of the *M. marinum* and Mtb phagosomes in murine phagocytes (Kolonko et al., 2014). In addition to Arp2/3 and WASH, the *D. discoideum* homolog of mammalian flotillin, vacuolin B (VacB), is also essential for the establishment of the MCV, assisting *M. marinum* in the retrieval of the vATPase from its niche. On the contrary, the small GTPase RacH of *D. discoideum* contributes to the maturation of the MCV into phagolysosomes, reducing the intracellular bacterial load (Hagedorn and Soldati, 2007) (Supplementary Table 3).

It has to be noticed that *M. marinum* does not interfere with the contractile vacuole of *D. discoideum* (Kolonko et al., 2014). This osmoregulatory organelle consists of interconnected vesicles, cisternae and tubules that fuse with the plasma membrane to expel the excess of water ingested during macropinocytosis and maintain cell volume (Patterson, 1980). The contractile vacuole accumulates Rab11 GTPase, vATPase, and calmodulin (Du et al., 2008), but the latter does not localize to the MCV (Kolonko et al., 2014; Cardenal-Muñoz et al., 2017). In addition, tetramethylrhodamine isothiocyanate (TRITC)-dextran, which labels the endolysosomal system but not the contractile vacuole (Clarke et al., 2002), can be found inside the MCV (Gerstenmaier et al., 2015).

### *M. marinum* proliferation phase (~12–37 hpi)

After establishment as a replication-permissive niche, the MCV becomes more spacious (Figures 1, 2 and Table 1). It still harbors Rab7a and, as occurs in human monocytes, it accumulates the post-lysosomal marker vacuolin/flotillin (Hagedorn and Soldati, 2007; Barisch et al., 2015b). On the contrary, VatA association with MCVs significantly decreases (Kolonko et al., 2014). Intriguingly, WASH contributes to *M. marinum* growth, and the WASH and Arp2/3 complexes can be observed at the MCV at 24 hpi (Kolonko et al., 2014). However, the F-actin coat of the MCV is almost completely lost and treatment with LatA does not alter the association of p80 or VatA with the MCV (Kolonko et al., 2014). Kil2, a *D. discoideum* P-type ATPase involved in intra-phagosomal killing of *Klebsiella*, does not play any role in the establishment of the MCV nor in *M. marinum* intracellular replication (Lelong et al., 2011). In addition, the *D. discoideum* homolog of coronin, a mammalian protein involved in the organization of the actin cytoskeleton that positively modulates intracellular mycobacterial growth in macrophages (Jayachandran et al., 2007), appears heterogeneously localized at the MCV but seems to negatively regulate the uptake or early survival and proliferation of *M. marinum* (Solomon et al., 2003). These data suggest that other host factors must be responsible for the support of the *M. marinum* intracellular replication in this amoeba.

One of the bacterial effectors required for the successful proliferation of *M. marinum* within *D. discoideum* is MAG24-1 (Solomon et al., 2003; Hagedorn and Soldati, 2007), a PE-PGRS protein also essential for replication in mammalian macrophages, frogs and flies (Ramakrishnan et al., 2000; Dionne et al., 2003). Actually, due to its role in intracellular growth, MAG24-1 can be used as a tool to monitor the proliferation of *M. marinum* mutant strains in *D. discoideum* by simply following the expression of GFP under the control of the *mag24-1* promoter in bacteria carrying the map24::GFP vector (Hagedorn and Soldati, 2007) (Supplementary Table 1). Another factor essential for optimal *M. marinum* intracellular growth is the ESX-1 secretion system, responsible for the rupture of the phagosomes containing *M. marinum* and its release into *D. discoideum* cytosol at the end of this infection phase (21–37 hpi) (Hagedorn and Soldati, 2007; Hagedorn et al., 2009). Mtb and *M. avium* can be seen in *D. discoideum* within spacious compartments that accumulate vacuolin but, contrary to Mtb, the ESX-1-deficient *M. avium* (Houben et al., 2014) does not escape its MCV (Hagedorn et al., 2009). A functional ESX-1 is important for optimal *M. marinum* proliferation (Hagedorn et al., 2009; Cardenal-Muñoz et al., 2017), and the exogenous expression of *M. marinum* ESAT-6 in *D. discoideum* rescues the inefficient intracellular replication of ΔRD1 bacteria (Hagedorn et al., 2009). The role of ESX-1 in phagosomal escape and intracellular proliferation of *M. marinum* and Mtb has also been described in mammalian cells (Stamm et al., 2003; Gao et al., 2004; Volkman et al., 2004; Tan et al., 2006; Simeone et al., 2015; Zhang et al., 2016). In addition to ESX-1, other mycobacterial factors have been shown to contribute to phagosomal rupture. The mycobacterial cell wall components phthiocerol dimycocerosates (PDIMs) have been revealed essential for ESAT-6 activity, suggesting some sort of cooperativity not yet understood (Augenstreich et al., 2017).

The *D. discoideum* ammonium transporter AmtA is a useful marker to determine whether or not mycobacteria are inside a compartment, since its fluorescent versions ubiquitously localize to all the endosomes and the MCV (Barisch et al., 2015b; Barisch and Soldati, 2017b) (Supplementary Table 4). Methods based on fluorescence resonance energy transfer (FRET), such as FRET-based microscopy or flow cytometry, have been performed in macrophages infected with Mtb and *M. marinum* to detect the mycobacteria-mediated phagosome disruption (Simeone et al., 2012, 2015; Acosta et al., 2014).

### Late *M. marinum* infection phase (~37–72 hpi)

After 37 hpi, when *M. marinum* has escaped its MCV, the intracellular bacterial load stabilizes and even decreases (Hagedorn and Soldati, 2007). This might be the result of the convergence between recapture and killing of cytosolic bacteria in lytic compartments, and the release of the bacteria to the extracellular medium or neighboring cells (Figure 1). Events of recapture have not been reported so far in *D. discoideum*, but in macrophages cytosolic *M. marinum* is ubiquitinated and recaptured into non-autophagic double membrane compartments (LAMP-1-positive, LC3/Atg8-negative) (Collins et al., 2009). It was proposed that ESCRT, a protein machinery that sorts ubiquitinated cargo to degradation into intraluminal vesicles (Schmidt and Teis, 2012), would deliver these bacteria to multivesicular bodies (MVB) and finally to lysosomes, but the bactericidal activity of these putative recapture compartments was not established (Collins et al., 2009).

Regarding release, and as a consequence of the wide range of strategies used by bacteria to survive and replicate within their hosts, multiple egress strategies have emerged in the course of evolution (Friedrich et al., 2012) (Figure 1 and Table 1). In some cases, egress implies the disruption of one or several host membranes, which may lead to the host cell death. Mycobacteria modulate the death of their host cells, inducing apoptotic or necrotic processes with different biological implications. These processes vary depending on the mycobacterial species, host cell types, and infection stage (reviewed in Aguilo et al., 2013; Srinivasan et al., 2014). In this context, many studies have associated ESX-1 activity and mycobacterial vacuolar escape to an increased host cell cytotoxicity and death (Hsu et al., 2003; Derrick and Morris, 2007; Kaku et al., 2007; Kinhikar et al., 2010; Simeone et al., 2012; Augenstreich et al., 2017). Thus, incubation with purified ESAT-6, as well as infection with wild-type (wt) Mtb and *M. marinum* but not with ΔRD1 mutant bacteria, lyse epithelial and red blood cells and macrophages (Hsu et al., 2003; Gao et al., 2004). This is of special relevance since mycobacterial egress from its host might influence spread of the infection in a tissue. Similarly, by using a microfluidic device (the InfectChip) to microscopically monitor single-cell long-term infection in *D. discoideum*, it has been shown that a quarter of the phagocytosed *M. marinum* induces death and lysis of the amoebae, contributing to bacteria release (Delince et al., 2016).

Alternative non-lytic mechanisms of bacterial egress also occur during mycobacterial infection in *D. discoideum*. For instance, release of *M. marinum* within the first hours of infection, when most of the bacilli are still intravacuolar, thereby suggesting an exocytic process, can be induced by mild starvation of *D. discoideum* (Delince et al., 2016). This phenomenon is likely akin to the known massive exocytosis of lysosomal enzymes that *D. discoideum* undergoes upon starvation (Smith et al., 2010). In addition, bacterial ejection occurs at later time points (or earlier if the MOI is increased to 50–100, Gerstenmaier et al., 2015) once mycobacteria have escaped the MCV and accessed the host cytosol (Hagedorn et al., 2009). During ejection, *M. marinum* and Mtb, but not the ESX-1-deficient *M. avium*, use the actin cytoskeleton of *D. discoideum* to form an actin-based short barrel structure formed at the plasma membrane, called the ejectosome, through which they exit the cell (Hagedorn et al., 2009). In this context, some *D. discoideum* actin-binding proteins such as coronin and myosin IB are enriched at the ejectosome (Hagedorn et al., 2009) (Supplementary Table 4). In addition, ejection cannot occur in amoebae lacking the actin polymerization complex WASH (Kolonko et al., 2014). Strikingly, other markers of the actin cytoskeleton such as the actin nucleator complex Arp2/3 and myosin II are not enriched at the ejectosome, suggesting that this structure may assemble *de novo* during ejection (Hagedorn et al., 2009). It has been proposed that the *D. discoideum* plasma membrane reseals at the posterior of the bacterium after ejection, preventing the amoebal lysis or leakage (Gerstenmaier et al., 2015) (section *D. discoideum* Plasma Membrane Damage and Sealing at Later Time Points). Exclusion of DAPI (a membrane-impermeant DNA binding dye) from the nuclei of live amoebae during ejection demonstrates that *M. marnium* does not kill or induce leakage of its host while egressing by this mean (Hagedorn et al., 2009).

Exocytosis and ejection can be discriminated by fluorescence microscopy with various *D. discoideum* markers. For instance, only during exocytosis the intracellular pole of the egressing bacterium is positive for endosomal and MCV markers such as p80 and vacuolins. In addition, the extracellular part of the exocytosed bacterium is free of any plasma membrane marker (Hagedorn et al., 2009; Gerstenmaier et al., 2015). On the contrary, upon ejection, extracellular Mtb and *M. marinum* are wrapped in plasma membrane remnants positive for p80 or PM4C4 (Supplementary Table 4). Clumps of bacteria can be exocytosed, but ejectosomes, which are often observed to form clusters, only release individual bacteria (Hagedorn et al., 2009).

Once bacteria exit the cell, neighboring cells might become new hosts. Within macrophages, a subpopulation of cytosolic *M. marinum* sheds its ubiquitinated cell wall possibly to avoid recapture into lysosomes. These bacteria induce the polymerization of actin tails and spread via actin-dense filopodia that can be caught by other cells (Stamm et al., 2003; Collins et al., 2009; Hagedorn et al., 2009). However, and presumably due to the high rates of actin turnover, cytosolic *M. marinum* does not form persistent actin tails in *D. discoideum*, but events of cell-to-cell transmission synchronized with actin-based ejection are observed (Hagedorn et al., 2009). *D. discoideum* RacH, mentioned before to contribute to the maturation of the MCV, also assists *M. marinum* in its release from the amoeba and the subsequent intercellular dissemination. Accordingly, ejection of *M. marinum* is not observed in *racH*-cells (Hagedorn et al., 2009), and the population of infected *racH*- amoebae almost fully retain all bacteria until 37 hpi (Hagedorn and Soldati, 2007; Hagedorn et al., 2009) (Supplementary Table 3). In agreement with these results, quantitative dissemination assays have confirmed the positive role of RacH in *M. marinum* cell-to-cell spread. In these fluorescence microscopy assays, an unlabeled donor *D. discoideum* strain (wt or *racH*-) was infected with red fluorescent *M. marinum*, and a wt acceptor strain expressing GFP was added to the infection after 12 h. Transmission from *racH*- infected cells to wt cells was reduced over eight-fold (Hagedorn et al., 2009).

## *M. marinum* induces membrane damage that is counteracted by *D. discoideum*

Rupture of host membranes by bacterial pathogens is achieved by the well-orchestrated secretion of lipases, proteases and pore-forming toxins. This is the case of vacuolar bacteria such as *Mycobacterium, Shigella, Listeria*, or *Salmonella*, which escape to the host cytosol via the disruption of their vacuole. Although the membranolytic activity of the mycobacteria ESX-1 secretion system is widely accepted, it has been proposed that ESX-1-dependent membrane disruptions do not require ESAT-6 activity in physiological conditions in macrophages (Conrad et al., 2017). Instead, the perforations would occur upon direct contact of Mtb and *M. marinum* ESX-1 with host MCV membranes (Conrad et al., 2017), which would confine the membranolytic activity to the bacterial poles, where the ESX-1 is enriched (Carlsson et al., 2009). Incompatible with this proposed model, *M. marinum* mutants specifically lacking only ESAT-6 and CFP10 (ΔCE) in an otherwise intact RD1 locus are defective in hemolysis, cytolysis, and cytotoxicity (Gao et al., 2004). Similarly, the virulent Mtb H37Rv strain with a 12 amino acids deletion in ESAT-6 shows impaired membranolytic activity in the THP-1 human macrophages (Houben et al., 2012). Regarding CFP10, it has been shown that the protein dissociates from ESAT-6 in acidic conditions similar to those encountered within the phagosome, and it does not disrupt artificial liposomes on its own (de Jonge et al., 2007). As a consequence, CFP10 has been proposed to act as a chaperone of ESAT-6, conferring stability or protection against proteases. Despite the fact that the structures of ESAT-6 and CFP10 have been solved (Renshaw et al., 2005), it is not yet well-understood how ESAT-6 induces membrane disruptions.

### Perforation and repair of the MCV membrane at early time points

*M. marinum* resides inside a phagosome that shields it against the host intracellular immune responses but limits nutrient access. Maybe as a reaction, the bacterium creates a porous vacuole which might appear ideal to obtain unrestricted access to nutrients. Indeed, very early during infection of *D. discoideum, M. marinum* induces ESX-1-dependent damages in the MCV membrane, which results in the decoration of cytosol-exposed bacteria with Ub already at 1.5 hpi (Cardenal-Muñoz et al., 2017). This MCV membrane damage leads to a well-orchestrated early autophagic response consisting in: (i) transient downregulation of TORC1 and upregulation of transcription of autophagy genes such as *atg1*, the two paralogs *atg8a* and *atg8b*, and *p62*, (ii) enhanced formation of autophagosomes, and (iii) recruitment of the xenophagy machinery (Ub, GFP-p62, and Atg8a) to the damaged MCV. These responses to MCV damage are significantly reduced or even abolished when *M. marinum* lacks the RD1 locus and, more specifically, ESAT-6 and CFP-10 (Cardenal-Muñoz et al., 2017).

However, contrary to the increased bacterial killing expected from an enhanced xenophagic response, *M. marinum* survives and proliferates within *D. discoideum* by blocking the autophagic flux and its subsequent degradation within autolysosomes (Cardenal-Muñoz et al., 2017). Once again, the main player involved in this blockage is the mycobacterial ESX-1 secretion system, also shown to block the autophagic flux in human cells infected with Mtb (Romagnoli et al., 2012). Along these lines, even though 40–50% of the early *M. marinum* MCVs are positive for the autophagosomal marker Atg8a and for markers of acidic compartments [vATPase LysoSensor Green and neutral red (NR) (Hagedorn and Soldati, 2007; Kolonko et al., 2014; Cardenal-Muñoz et al., 2017)], and assuming that the presence of Rab7 on the MCV (Cardenal-Muñoz et al., 2017) should ensure fusion with late endosomes and lysosomes (Zhang et al., 2009), the MCVs containing wt bacteria are devoid of lysosomal enzymes and degradative activity, as demonstrated by the lack of cathepsin D and proteolysis-dependent DQ-Green BSA fluorescence (Hagedorn and Soldati, 2007; Cardenal-Muñoz et al., 2017). This suggests that the ESX-1-induced membrane damage make MCVs leaky for hydrolytic enzymes. This MCV leakage, together with the retrieval of the vATPase, and possibly hydrolases, by WASH- and Arp2/3-dependent polymerization of actin[see section Early *M. marinum* Infection Phase (~0–12 hpi) and Figure 2], would affect both autophagic flux and killing of mycobacteria. Interestingly, depletion of hydrolases from the acidic *Salmonella*-containing compartment (SCV) has already been described, although the mechanism is different. *Salmonella* subverts the Rab9-dependent retrograde trafficking of mannose-6-phosphate receptors in human cells, which results in fusion of the SCV with lysosomes that are reduced in hydrolytic enzymes (McGourty et al., 2012).

The membrane damage inflicted by *Salmonella* induces an amino acid starvation response in the host cell that inhibits TOR and induces autophagy and membrane repair, leading to the normalization of the amino acid levels and the reactivation of TOR at the surface of SCVs (Tattoli et al., 2012a,b; Kreibich et al., 2015). Interestingly, it has been recently shown that mammalian TRIM16, a member of the TRIM family, recognizes the endomembrane perturbations induced by Mtb ESX-1 and affects TORC1 activity, inducing autophagy to protect macrophages from damage (Chauhan et al., 2016). The role of autophagy in mediating membrane repair during *D. discoideum* infection with *M. marinum* has also been recently proposed (Cardenal-Muñoz et al., 2017). After downregulation of TORC1 and induction of the autophagic response mentioned above, *D. discoideum* Lst8 (a TORC1 component) and two TORC1 activators, the Rheb GTPase and Lamtor1 [a member of the guanine nucleotide exchange factor (GEF) Ragulator complex] localize to damaged MCVs coinciding with reactivation of TORC1 at 7 hpi (Cardenal-Muñoz et al., 2017) (Figure 2). In addition, EM inspection of infected amoebae lacking the major autophagy initiator Atg1 (ULK in humans) (Mesquita et al., 2015) reveals that most bacteria escape very early to the cytosol (1 hpi), followed by a striking increased load of highly ubiquitinated cytosolic bacteria that cannot be degraded by xenophagy. This suggests that the autophagy pathway of *D. discoideum* is required for the repair of the MCV membranes, preventing the escape of mycobacteria to the cytosol. It remains unclear why *M. marinum* accelerates its growth only after 24 hpi in the *atg1*-amoebae (Cardenal-Muñoz et al., 2017). One plausible explanation is that autophagy would not only recognize damage and repair the MCV, but it could also provide *M. marinum* with cytoplasmic nutrients such as membranes and lipid droplets (LDs) by the fusion of autophagosomes with the MCV (see section *M. marinum* Exploits *D. discoideum* Lipids).

### *D. discoideum* plasma membrane damage and sealing at later time points

Despite the function of autophagy in membrane repair during early infection, most *M. marinum* bacilli are able to break completely their MCV and finally access the *D. discoideum* cytosol (Hagedorn and Soldati, 2007). As previously described, *M. marinum* perforates the plasma membrane of *D. discoideum* to egress through an actin-based ejectosome (Hagedorn et al., 2009). The ESX1 secretion system, and more specifically secreted ESAT-6, are required for ejection of the bacteria (Hagedorn et al., 2009). This is demonstrated by restoration of ejection of the ΔRD1 mutant by ectopic expression of *M. marinum* ESAT-6 in *D. discoideum* (Hagedorn et al., 2009). Despite this perforation, the integrity of the *D. discoideum* plasma membrane is continuously maintained during and after ejection by the autophagy machinery (Figure 1). This is necessary to avoid lytic cell death and to improve cell-to-cell transmission of the bacteria (Gerstenmaier et al., 2015). Accordingly, the autophagosomal markers Atg8, GFP-p62, and Ub are present at a pocket formed around the intracellular distal pole of ejecting *M. marinum* (Gerstenmaier et al., 2015). On the contrary, GFP-Atg18 and GFP-2xFYVE, two reporters of expanding phagophores (Calvo-Garrido et al., 2014; Mesquita et al., 2016), are less or rarely present, respectively (Gerstenmaier et al., 2015). This, together with the fact that the transcription levels of autophagy genes remain unchanged at these late stages of infection (Cardenal-Muñoz et al., 2017), suggest that *M. marinum* exploits pre-formed autophagosomes during its ejection.

The core autophagy machinery of *D. discoideum* (Atg1, Atg5, Atg6, and Atg7) is required for the recruitment of Atg8 to the distal pole of ejecting *M. marinum* (Supplementary Table 3). Strikingly, *atg1*- cells can form the actin-based structure of ejectosomes, but these are not functional for cell-to-cell transmission (Gerstenmaier et al., 2015). In addition, the selective receptor p62 is not required for the recruitment of Atg8 to the ejecting bacterium (Gerstenmaier et al., 2015). This, together with the fact that *M. marinum* grows normally within *p62*-cells (Cardenal-Muñoz et al., 2017), suggest that alternative autophagy receptors might exist in *D. discoideum*. Interesingly, the ESX-1 secretion system, which induces the recruitment of autophagic markers to bacteria escaping the MCV (Cardenal-Muñoz et al., 2017), does not play a role in the localization of Atg8 to bacteria during ejection (Gerstenmaier et al., 2015). This was shown by co-infecting cells with wt and either *M. marinum* ΔRD1 or the non-pathogenic *M. smegmatis* (whose ESAT-6 is inactive in membrane disruption). In these cells both bacteria form ejectosomes and recruit Atg8 to their distal pole (Gerstenmaier et al., 2015).

## *M. marinum* exploits *D. discoideum* lipids

Inside their hosts, pathogens are restricted to a limited supply of nutrients. For instance, to drive proliferation, intracellular bacteria need to exploit a suitable energy source (Abu Kwaik, 2015; Abu Kwaik and Bumann, 2015). In recent years, it has become evident that *Mycobacterium* spp. access host lipids (Peyron et al., 2008; Caire-Brandli et al., 2014) to gain energy via β-oxidation and the glyoxylate shunt (McKinney et al., 2000; Munoz-Elias and McKinney, 2005). The importance of lipids for Mtb, but also for other *Mycobacterium* spp., is supported by the high number of genes (more than 250) harbored in the Mtb genome and required for lipid and fatty acid (FA) metabolism (Cole et al., 1998). The encoded proteins have diverse roles in cell wall synthesis, formation of intracytosolic lipid inclusions (ILIs) and production of energy (reviewed in Dumas and Haanappel, 2017). Strikingly, 100 of these genes exclusively encode proteins involved in the five reactions of β-oxidation (Cole et al., 1998). Interestingly, the chromosome of *M. marinum* is 50% larger than the one of Mtb (6.6 million base pairs compared to 4.4 million), which is also reflected by a higher number of genes involved in lipid metabolism (Stinear et al., 2008). For instance, the genome of *M. marinum* encodes 32 *fadD* acyl-CoA synthase paralog, compared with 23 in Mtb. This larger genome likely reflects the capacity of *M. marinum* to infect and produce a tuberculosis-like disease in a larger range of environmental hosts compared to Mtb (Stinear et al., 2000).

### Lipid droplets are recruited to the MCV during the early infection phase

**Figure 3:**
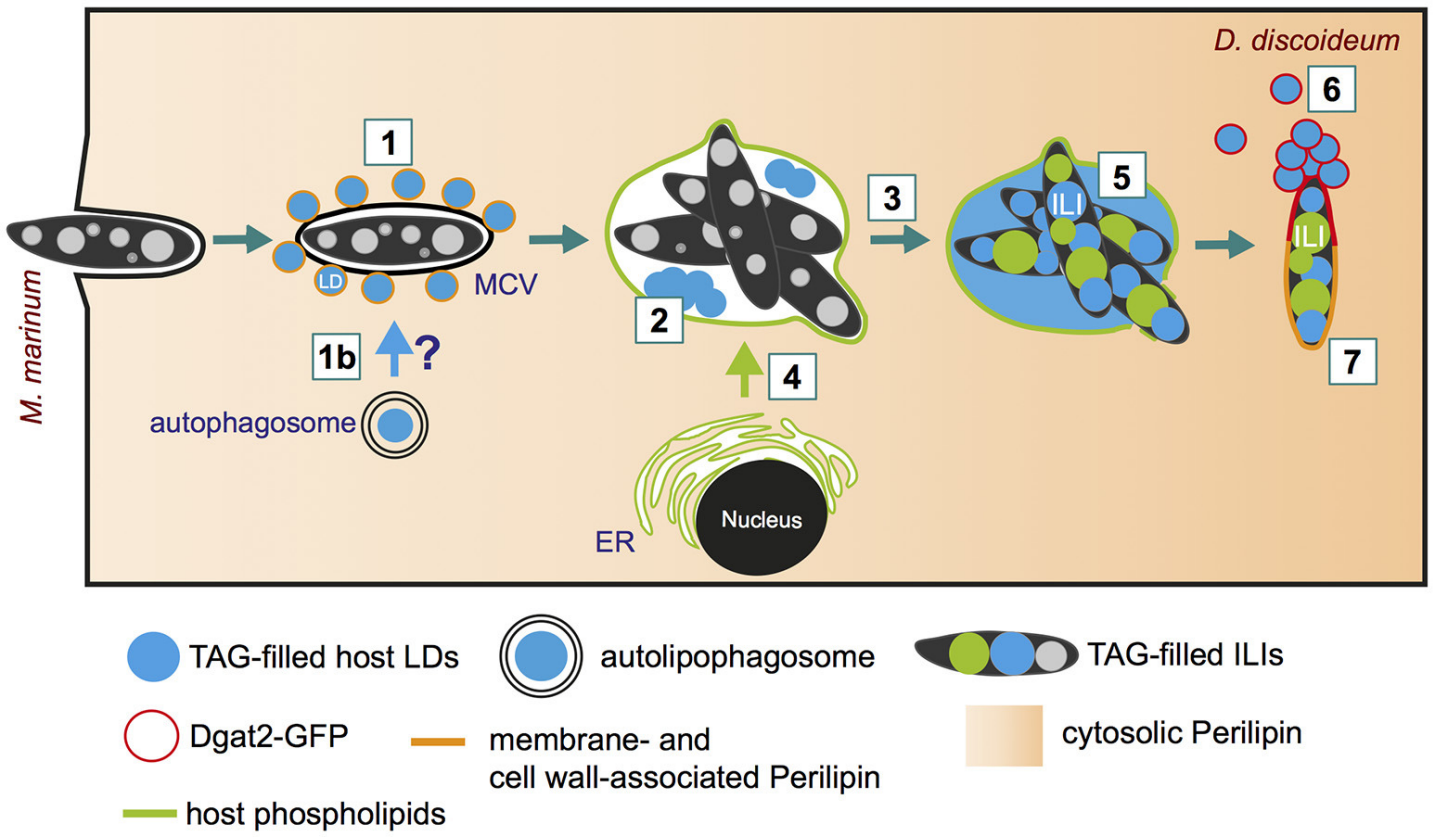
Lipid distribution and re-arrangement during infection. (1) Soon after bacteria uptake (10 min post-infection), host lipid droplets (LDs) are clustered around the MCV (Barisch et al., 2015b). (1b) Indicates the possibility of LDs capture and translocation via lipophagy (Barisch and Soldati, 2017a); (2) LDs might be imported into the MCV by a mechanism similar to phagosomal fusion; (3) Neutral lipids (and sterols) accumulate within the MCV at late infection stages; (4) Host phospholipids are transferred to the MCV by membrane trafficking (Barisch and Soldati, 2017b); (5) Host triacylglycerols (TAGs) and phospholipids serve as fatty acid (FA) source for bacterial intracytosolic lipid inclusion (ILI) formation (Barisch et al., 2015b); (6) Dgat2-positive LDs aggregate at bacteria poles as soon as the MCV breaks, leading to the coalescence of LDs onto the *M. marinum* cell wall and the complete surrounding of the bacteria by Dgat2 (Barisch and Soldati, 2017a); (7) The *D. discoideum* homolog of perilipin reaches *M. marinum* from the cytosol and targets the bacterial cell wall with the help of amphipathic and hydrophobic domains (Barisch et al., 2015b; Barisch and Soldati, 2017b).

Despite the increasing evidence pointing to a central role of lipids during Mtb infection, the basic cellular mechanisms by which pathogenic mycobacteria exploit host lipids are still poorly understood. One of the major characteristics of tuberculosis is the appearance of foamy macrophages during chronic infection. The accumulation of LDs that leads to the foamy characteristics of the respective host cells was observed during Mtb infection (reviewed in Russell et al., 2009), but also in infections with *M. bovis* (D'Avila et al., 2006) and *M. leprae* (de Mattos et al., 2012). By using *D. discoideum* as a model for foamy macrophages, it was recently shown that host LDs move to the MCV within minutes after phagocytosis (Figure 3, step 1; Barisch et al., 2015b). The clustering of host LDs at the MCV can be monitored by live microscopy using Bodipy 493/503 as a neutral lipid marker for LDs that were induced by feeding cells with exogenous FAs prior to infection. We propose that host LDs might be translocated into the MCV by a process similar to lipophagy (Figure 3, step 1b), a selective autophagy pathway for LDs (reviewed in Schulze et al., 2017), or by a fusion-like process similar to that observed in a macrophage/*M. avium* infection model system (Caire-Brandli et al., 2014) (Figure 3, step 2). In analogy to LD-bilayer or LD-LD fusion (Thiam et al., 2013), the phospholipid monolayer of an LD might first fuse with the outer leaflet of the MCV membrane leading to the release of the hydrophobic core lipids into the membrane bilayer of the MCV. In a second step, a new LD-like structure might bud into the lumen of the MCV (for more detailed information, please see (Barisch and Soldati, 2017a).

### Host lipid distribution at the *M. marinum* proliferation phase

At later infection stages (19 hpi), LD-like structures disappear, but neutral lipids and sterols, which can be detected by Bodipy 493/503 and filipin staining, are homogenously distributed within the MCV (Figure 3, step 3; Barisch et al., 2015b). Interestingly, these observations are in agreement with the fact that mycobacteria are one of the rare species able to catabolize host sterols and, more precisely, cholesterol (Pandey and Sassetti, 2008; Senaratne et al., 2008). By labeling *D. discoideum* with Topfluor-Lysophosphatidylcholine (Topfluor-LysoPC), a fluorescent phospholipid precursor, it was observed by live microscopy that host phospholipids accumulate at the membranes of the MCV, most likely by standard membrane trafficking (Figure 3, step 4; Barisch and Soldati, 2017b).

Strikingly, intravacuolar bacteria accumulate considerably more ILIs when *D. discoideum* cells are treated with exogenous FAs (Figure 3, step 5). Not only the FAs released from host triacylglycerols (TAGs) but also those from phospholipids are used by *M. marinum* for ILI formation (Figure 3, step 5; Barisch and Soldati, 2017b). This was shown by thin layer chromatography (TLC) using a *D. discoideum dgat1/2* double mutant, which is depleted in both diacyglycerol O-acyltransferases Dgat1 and Dgat2. This mutant shuttles excessive lipids into phospholipids, and does not synthesize TAGs and generate LDs (Du et al., 2013; Barisch and Soldati, 2017b). Interestingly, by measuring bacteria growth with the help of high-content microscopy and a luminescence-based assay, it was observed that *M. marinum* growth was unaffected in the *dgat1/2* double mutant (Barisch and Soldati, 2017b) leading to the conclusion that host phospholipids can successfully substitute for host TAGs. The transfer of host lipids to the intracellular pathogen can also be monitored by live-cell microscopy. To this end, host cells are labeled with Topfluor-LysoPC prior to infection. Strikingly, fluorescently-labeled host phospholipids become first enriched at the membrane of the MCV 10 min post-infection, and inside the bacteria at 21 hpi. Consequently, it was proposed that host lipids are transferred to the MCV by membrane trafficking and then further processed by bacterial or host phospholipases to facilitate uptake of FAs into the bacteria (Barisch and Soldati, 2017b).

Recently, the question whether Mtb has enzymes to successfully cleave/hydrolyse host lipids was addressed (Singh et al., 2017). The authors propose that host lipids are cleaved by Msh1, which is upregulated upon hypoxic conditions, and secreted into the host cytosol, where it hydrolyses TAGs. For *M. marinum*, it has been shown that the formation of ILIs depends on its type VII secretion system ESX-5, presumably thanks to the absorption of nutrients such as FAs through membrane porins or channels that are formed into the outer membrane of *M. marinum* by this ESX-5 apparatus (Ates et al., 2015). In the case of Mtb, FAs are transported into the bacteria via the mammalian cell entry 1 (Mce1) complex and LucA that interacts with subunits of the complex and coordinates its activity, and which is required for full virulence of Mtb *in vivo* (Nazarova et al., 2017).

The reason why intracellular mycobacteria accumulate ILIs during infection is so far poorly understood. One possibility is that these bacteria use ILIs as an energy source. For instance, it has been shown that in macrophages infected with *M. avium* and without any additional fat source, LDs are rapidly depleted from the macrophages, immediately followed by ILIs depletion from the pathogens (Caire-Brandli et al., 2014). Moreover, with the help of an *in vitro* dormancy model, it was proposed that *M. bovis* BCG uses the TAGs released from ILIs as an energy source during reactivation from dormancy (Low et al., 2009). In addition, when the synthesis of TAGs is inhibited in *Drosophila melanogaster*, the accumulation of host LDs induced by *M. marinum* infection is also prevented, and the mycobacteria-LDs association, as well as the number of intracellular viable *M. marinum*, are reduced (Pean et al., 2017). Furthermore, ILI-rich mycobacteria have been shown to be more tolerant to rifampicin, isoniazid, ethambutol, and ciprofloxacin (Hammond et al., 2015). Finally, an antioxidant role of LDs (or ILIs) was proposed recently (Bailey et al., 2015).

Importantly, host lipids might not only be used by these bacteria as an energy source but also as building blocks for cell wall lipids. The core of the mycobacterial cell wall consists of mycolic acids that are esterified to arabinogalactan (AGs) polysaccharides, which are in turn covalently attached to a peptidoglycan backbone (reviewed in Brennan, 2003; Jackson, 2014). This core structure serves as anchor for extractible “free lipids” such as PDIMs and phenolic glycolipids (PGLs). One unique feature of pathogenic *Mycobacterium* spp. is their antibiotic resistance that is conferred by the “mycomembrane,” the impermeable lipid bilayer that is formed by the mycolic acids and the free lipids (Jackson, 2014). During infection, PDIMs are involved in Mtb uptake (Astarie-Dequeker et al., 2009), block of phagosomal maturation and acidification arrest (Pethe et al., 2004; Stewart et al., 2005), in escape to the cytosol and egress from the host cells (Quigley et al., 2017). In addition, PDIMs are essential for the multiplication and persistence of Mtb in the lungs of infected mice (Cox et al., 1999), and Mtb strains inhibited in PDIM synthesis are more susceptible to killing by an early innate host response (Day et al., 2014). Corroborating this, it has been demonstrated that FAs released from host LDs are used by Mtb to synthesize PDIMs (Lee et al., 2013).

PDIMs are also implicated in the virulence of *M. marinum*. Like Mtb in macrophages, *M. marinum* synthesizes PDIMs (and TAGs) from FAs released from *D. discoideum* LDs and phospholipids (Barisch and Soldati, 2017b). To monitor lipid transfer from the host to the pathogen, *D. discoideum* lipids can be labeled by using fluorescent FAs such as Bodipy 558/568 C12 or Topfluor-LysoPC prior to infection (Barisch and Soldati, 2017b). The incorporation of the fluorescent label into *D. discoideum* and *M. marinum* lipids can be detected by TLC and fluorescent lipid species can be identified with the help of fluorescent standard lipids.

A transposon insertion in the *M. marinum tesA* gene, encoding a putative type II thioesterase, leads to an altered cell wall without PDIMs and PGLs and the subsequent lack of the permeability barrier (Alibaud et al., 2011). As a consequence, the *M. marinum tesA* mutant is attenuated in *D. discoideum* and in zebrafish embryos (Alibaud et al., 2011). Inhibitor and gene mutations altering the PDIMs and PGLs of *M. marinum* affect its virulence in *D. discoideum* and adult zebrafish, respectively (Yu et al., 2012; Ouertatani-Sakouhi et al., 2017). In addition, Cambier et al., elegantly described how Mtb and *M. marinum* preferentially recruit and infect permissive macrophages while microbicidal ones are evaded (Cambier et al., 2014). This bypass of the innate immune system was proposed to be the result of cell-surface-associated PDIMs hiding underlying pathogen-associated molecular patterns (PAMPs).

### LD proteins target cytosolic *M. marinum* at late infection phases

At later infection stages (42 hpi), LD proteins such as the *D. discoideum* homolog of perilipin as well as Dgat2 localize to cytosolic *M. marinum* (Figure 3, steps 6 and 7; Barisch et al., 2015b; Barisch and Soldati, 2017b). Perilipin belongs to the class II of LD proteins, which bind the LD surface through hydrophobic and amphipatic domains, and circulates between LDs and the cytosol. During infection, perilipin is proposed to partition into the waxy mycobacterial cell wall in a similar way, via its amphipathic and hydrophobic regions (Barisch and Soldati, 2017a). Importantly, by using a luminescence-based assay to measure intracellular *M. marinum* growth, it became evident that in a *D. discoideum* perilipin (*plnA*) mutant, bacteria growth is inhibited starting from 20 hpi, the time at which *M. marinum* reaches the cytosol (Barisch et al., 2015b). Consequently, it was proposed that perilipin might exert a barrier function, protecting the *M. marinum* cell wall during the cytosolic phase of the infection from degradative host processes such as neutral lipases.

Dgat2 belongs to the class I of LD proteins, which bind the LD surface through an hydrophobic hairpin motif, and is exclusively localized at LDs. Consequently, Dgat2 is transferred to the waxy cell wall of *M. marinum* by coalescence with LDs and lateral diffusion (Barisch and Soldati, 2017b).

## The end of the romance: using *D. discoideum* to uncover therapeutic targets in *M. marinum*

### *D. discoideum* plaque assays

As for animals, *M. marinum* is pathogenic for *D. discoideum*. Contrary to the food bacterium *Klebsiella*, the growth of *D. discoideum* is restricted when plated on *M. marinum* lawns (Ouertatani-Sakouhi et al., 2017). Researchers have made use of this growth incapacity to perform genetic and chemical screens for the search of virulence genes and anti-mycobacterial compounds. For instance, *M. marinum* TesA, a putative type II thioesterase required for the synthesis of the cell wall lipids PDIMs and PGLs, was identified in a *D. discoideum* plaque assay where a *M. marinum* transposon mutant library was screened for bacterial attenuation. The role of TesA in virulence was further confirmed during infection of zebrafish embryos, and the *tesA* gene was proposed as a possible genetic target for disruption in human mycobacterial pathogens (Alibaud et al., 2011). In addition, another plaque assay screen of *M. marinum* transposon mutants allowed Chen and collaborators to discover that two genes, *mmar_2318* and *mmar_2319*, involved in the biosynthesis of lipooligosaccharide (LOS) contribute to *M. marinum* virulence in *D. discoideum* and limit bacterial entry in human macrophages (Chen et al., 2015) (Supplementary Table 2). Chemical screens can also be performed in amoebae (Ouertatani-Sakouhi et al., 2017; Trofimov et al., in press). For instance, drugs targeting the *M. marinum* cell wall have been recently identified to block/decrease *M. marinum* virulence by inhibiting bacterial aggregation and permeability, sliding motility and/or intracellular replication within *D. discoideum* (Ouertatani-Sakouhi et al., 2017). All these findings highlight *D. discoideum* as an alternative host for the screening of *Mycobacterium* virulence factors and potential antibacterial compounds.

### High throughput screening

Since the advent of next-generation sequencing (NGS) technologies, numerous methods to investigate the host-pathogen interaction at different levels (i.e., RNA, DNA, and proteins) have been developed. Among them, RNA sequencing (RNA-Seq) is becoming the standard to analyze the transcripts of each actor involved in a bacterial infection, either independently (bacteria or host) or simultaneously (bacteria and host) by dual RNA-Seq (Saliba et al., 2017; Westermann et al., 2017). This method has been used to describe the gene expression profile of *M. marinum* during exponential and stationary growth (Wang et al., 2013), as well as under stress conditions (Pettersson et al., 2015). In addition, it has allowed to determine the contribution of specific cells (neutrophils and macrophages) to the innate immune response against *M. marinum*, Mtb, and *M. bovis* (Schnappinger et al., 2003; Nalpas et al., 2015; Kenyon et al., 2017) and to identify genes such as *whiB4*, which encodes a protein regulating the PE/PPE genes and is required for *M. marinum* and Mtb virulence (Chawla et al., 2012; Wu et al., 2017). Moreover, a recent metabolomics study coupled to dual RNA-Seq allowed to reconstruct the dietary map of Mtb during macrophage infection (Zimmermann et al., 2017). Since the RNA-Seq methodology has already been established in *D. discoideum* (Miranda et al., 2013), it will be of high interest to perform dual RNA-seq in the *D. discoideum*—*M. marinum* infection model.

Another NGS method is transposon sequencing (Tn-Seq), which allows the analysis of fitness and genetic interactions in microorganisms by using random transposon insertion libraries in bacteria (van Opijnen et al., 2009; DeJesus and Ioerger, 2013; DeJesus et al., 2013, 2017a,b; van Opijnen and Camilli, 2013; Nambi et al., 2015) Thanks to this method, various researchers have identified essential genes for Mtb growth and cholesterol metabolism (Griffin et al., 2011; Zhang et al., 2012; DeJesus and Ioerger, 2013) and have investigated the Mtb virulence factors required for survival in human dendritic cells (Mendum et al., 2015). Concerning *M. marinum*, only one study has been published so far (Weerdenburg et al., 2015), in which the authors identified ~300 genes essential for survival of the bacteria *in vitro*. These genes are mostly shared with Mtb and correspond to 6% of the total coding sequences. The authors investigated the strategies used by *M. marinum* to exploit different phagocytic cells, from protozoan and vertebrate origin. The behavior of the *M. marinum* transposon mutant pool was highly similar between the two mammalian cell lines (human THP-1 monocytes and mouse RAW264.7 macrophages) and between the two amoebae *D. discoidum* and *A. castellani*, while an intermediate behavior was observed in cells derived from fish (carp CLC leukocytes), probably due to their higher similarity with natural *M. marinum* hosts. Thus, the authors concluded that *M. marinum* has both conserved and host-specific virulence determinants. Regarding the conserved virulence factors, transposon interruption of PDIMs, ESX-1, or Mce1/4 leads to high attenuation of *M. marinum* virulence in multiple hosts. In addition, a new virulence factor of *M. marinum* was highlighted and validated *in vivo*, the *cpsA* gene. This gene encodes a protein of the LytR family of transcriptional regulators and has been recently demonstrated to be required for *M. marinum* cell wall integrity and virulence in the zebrafish model (Wang et al., 2015; Weerdenburg et al., 2015). Moreover, amoebae-specific pathways have been pointed out by the authors. For instance, transposon insertions in multiple genes involved in biosynthesis of vitamin B_12_ highly increased fitness of *M. marinum* in *Acanthamoeba*, while they had no or little impact on the bacteria within the other host cells (Weerdenburg et al., 2015). To conclude, *M. marinum* uses specific mechanisms to adapt to different intracellular environments. Some specific requirements for infection of mammalian cells are not applicable for protozoa infection, and *vice versa*.

Finally, REMI sequencing (REMI-Seq) has recently been developed for *D. discoideum* by the group of C. Thompson (https://thethompsonlab.wordpress.com/mutant-library/). The REMI method was first described in *Saccharomyces* (Schiestl and Petes, 1991) and then adapted to *D. discoideum* (Kuspa and Loomis, 1992; Kuspa, 2006). The technique comprises the integration of linear DNA into eukaryotic genomes for insertional mutagenesis. Combining REMI with NGS, a genome-wide collection of tagged mutants can be created to investigate host genes. However, REMI-Seq has not yet been used in the context of mycobacterial infection. Therefore, this tool, combined with other methods described above, are promising and innovative approaches that could help identify genes and pathways involved in mycobacteria-host interaction.

## Concluding remarks

In this review, we have described the so far known processes involved in the intracellular cycle of *M. marinum* within the amoeba *D. discoideum*. The comparison with studies carried out in human cells with Mtb revealed that the infection cycles of both bacteria share numerous similarities. However, many of the virulence mechanisms used by both pathogens to subvert the immune response of their hosts remain to be elucidated. We propose the *D. discoideum*—*M. marinum* system as a suitable and powerful model to study infection by tuberculous mycobacteria.

It needs to be noticed that not all the markers listed here were monitored in all the listed strains, and vice versa. Please see main text and supplementary tables for more details. IFA, immunofluorescence assay; TEM, transmission electron microscopy; qPCR, quantitative polymerase chain reaction; SEM, scanning electron microscopy; CLEM, correlative light and electron microscopy.

## Author contributions

EC-M, CB, LL, and AL-J wrote the original draft. EC-M and CB drew the figures and prepared the tables. EC-M, CB, LL, and TS reviewed and edited the draft. TS supervised this work.

### Conflict of interest statement

The authors declare that the research was conducted in the absence of any commercial or financial relationships that could be construed as a potential conflict of interest.

## References

[B1] Abdallah A. M. Gey van Pittius N. C. Champion P. A. Cox J. Luirink J. Vandenbroucke-Grauls C. M.. (2007). Type VII secretion–mycobacteria show the way. Nat. Rev. Microbiol. 5, 883–891. 10.1038/nrmicro1773 17922044

[B2] Abu Kwaik Y. (2015). Nutrition-based evolution of intracellular pathogens. Environ. Microbiol. Rep. 7, 2–3. 10.1111/1758-2229.12236 25721587

[B3] Abu Kwaik Y. Bumann D. (2015). Host delivery of favorite meals for intracellular pathogens. PLoS Pathog. 11:e1004866. 10.1371/journal.ppat.1004866 26110434 PMC4482385

[B4] Acosta Y. Zhang Q. Rahaman A. Ouellet H. Xiao C. Sun J.. (2014). Imaging cytosolic translocation of Mycobacteria with two-photon fluorescence resonance energy transfer microscopy. Biomed. Opt. Express 5, 3990–4001. 10.1364/BOE.5.003990 25426325 PMC4242033

[B5] Aguilo N. Marinova D. Martin C. Pardo J. (2013). ESX-1-induced apoptosis during mycobacterial infection: to be or not to be, that is the question. Front. Cell. Infect. Microbiol. 3:88 10.3389/fcimb.2013.00088 24364000 PMC3850411

[B6] Alibaud L. Rombouts Y. Trivelli X. Burguiere A. Cirillo S. L. Cirillo J. D.. (2011). A *Mycobacterium marinum* TesA mutant defective for major cell wall-associated lipids is highly attenuated in *Dictyostelium discoideum* and zebrafish embryos. Mol. Microbiol. 80, 919–934. 10.1111/j.1365-2958.2011.07618.x 21375593

[B7] Arafah S. Kicka S. Trofimov V. Hagedorn M. Andreu N. Wiles S.. (2013). Setting up and monitoring an infection of *Dictyostelium discoideum* with mycobacteria. Methods Mol. Biol. 983, 403–417. 10.1007/978-1-62703-302-2_22 23494320

[B8] Astarie-Dequeker C. Le Guyader L. Malaga W. Seaphanh F. K. Chalut C. Lopez A.. (2009). Phthiocerol dimycocerosates of *M. tuberculosis* participate in macrophage invasion by inducing changes in the organization of plasma membrane lipids. PLoS Pathog 5:e1000289. 10.1371/journal.ppat.1000289 19197369 PMC2632888

[B9] Ates L. S. Ummels R. Commandeur S. van de Weerd R. Sparrius M. Weerdenburg E.. (2015). Essential ROLE of the ESX-5 secretion system in outer membrane permeability of pathogenic mycobacteria. PLoS Genet. 11:e1005190. 10.1371/journal.pgen.1005190 25938982 PMC4418733

[B10] Aubry A. Mougari F. Reibel F. Cambau E. (2017). Mycobacterium marinum. Microbiol. Spectr. 5 10.1128/microbiolspec.TNMI7-0038-2016 PMC11687479 28387180

[B11] Augenstreich J. Arbues A. Simeone R. Haanappel E. Wegener A. Sayes F.. (2017). ESX-1 and phthiocerol dimycocerosates of *Mycobacterium tuberculosis* act in concert to cause phagosomal rupture and host cell apoptosis. Cell. Microbiol. 19:e12726. 10.1111/cmi.12726 28095608

[B12] Bailey A. P. Koster G. Guillermier C. Hirst E. M. MacRae J. I. Lechene C. P.. (2015). Antioxidant role for lipid droplets in a stem cell niche of Drosophila. Cell 163, 340–353. 10.1016/j.cell.2015.09.020 26451484 PMC4601084

[B13] Barisch C. López-Jiménez A. T. Soldati T. (2015a). Live imaging of *Mycobacterium marinum* infection in *Dictyostelium discoideum*. Methods Mol. Biol. 1285, 369–385. 10.1007/978-1-4939-2450-9_23 25779329

[B14] Barisch C. Paschke P. Hagedorn M. Maniak M. Soldati T. (2015b). Lipid droplet dynamics at early stages of *Mycobacterium marinum* infection in Dictyostelium. Cell. Microbiol. 17, 1332–1349. 10.1111/cmi.12437 25772333

[B15] Barisch C. Soldati T. (2017a). Breaking fat! How Mycobacteria and other intracellular pathogens manipulate lipid droplets. Biochimie 141, 54–61. 10.1016/j.biochi.2017.06.001 28587792

[B16] Barisch C. Soldati T. (2017b). *Mycobacterium marinum* degrades both triacylglycerols and phospholipids from its Dictyostelium host to synthesise its own triacylglycerols and generate lipid inclusions. PLoS Pathog. 13:e1006095. 10.1371/journal.ppat.1006095 28103313 PMC5245797

[B17] Barondes S. H. Cerra R. F. Cooper D. N. Haywood-Reid P. L. Roberson M. M. (1984). Localization of soluble endogenous lectins and their ligands at specific extracellular sites. Biol. Cell 51, 165–172. 10.1111/j.1768-322X.1984.tb00295.x 6240298

[B18] Barry N. P. Bretscher M. S. (2010). Dictyostelium amoebae and neutrophils can swim. Proc. Natl. Acad. Sci. U.S.A. 107, 11376–11380. 10.1073/pnas.1006327107 20534502 PMC2895083

[B19] Bitter W. Houben E. N. Bottai D. Brodin P. Brown E. J. Cox J. S.. (2009). Systematic genetic nomenclature for type VII secretion systems. PLoS Pathog. 5:e1000507. 10.1371/journal.ppat.1000507 19876390 PMC2763215

[B20] Bottai D. Di Luca M. Majlessi L. Frigui W. Simeone R. Sayes F.. (2012). Disruption of the ESX-5 system of *Mycobacterium tuberculosis* causes loss of PPE protein secretion, reduction of cell wall integrity and strong attenuation. Mol. Microbiol. 83, 1195–1209. 10.1111/j.1365-2958.2012.08001.x 22340629

[B21] Boulais J. Trost M. Landry C. R. Dieckmann R. Levy E. D. Soldati T.. (2010). Molecular characterization of the evolution of phagosomes. Mol. Syst. Biol. 6:423. 10.1038/msb.2010.80 20959821 PMC2990642

[B22] Boyle K. B. Randow F. (2013). The role of ‘eat-me’ signals and autophagy cargo receptors in innate immunity. Curr. Opin. Microbiol. 16, 339–348. 10.1016/j.mib.2013.03.010 23623150

[B23] Bozzaro S. Eichinger L. (2011). The professional phagocyte *Dictyostelium discoideum* as a model host for bacterial pathogens. Curr. Drug Targets 12, 942–954. 10.2174/138945011795677782 21366522 PMC3267156

[B24] Brennan P. J. (2003). Structure, function, and biogenesis of the cell wall of *Mycobacterium tuberculosis*. Tuberculosis 83, 91–97. 10.1016/S1472-9792(02)00089-6 12758196

[B25] Brenz Y. Ohnezeit D. Winther-Larsen H. C. Hagedorn M. (2017). Nramp1 and NrampB contribute to resistance against Francisella in Dictyostelium. Front. Cell. Infect. Microbiol. 7:282. 10.3389/fcimb.2017.00282 28680861 PMC5478718

[B26] Caire-Brändli I. Papadopoulos A. Malaga W. Marais D. Canaan S. Thilo L.. (2014). Reversible lipid accumulation and associated division arrest of *Mycobacterium avium* in lipoprotein-induced foamy macrophages may resemble key events during latency and reactivation of tuberculosis. Infect. Immun. 82, 476–490. 10.1128/IAI.01196-13 24478064 PMC3911402

[B27] Calvo-Garrido J. Carilla-Latorre S. Kubohara Y. Santos-Rodrigo N. Mesquita A. Soldati T.. (2010). Autophagy in Dictyostelium: genes and pathways, cell death and infection. Autophagy 6, 686–701. 10.4161/auto.6.6.12513 20603609

[B28] Calvo-Garrido J. Escalante R. (2010). Autophagy dysfunction and ubiquitin-positive protein aggregates in Dictyostelium cells lacking Vmp1. Autophagy 6, 100–109. 10.4161/auto.6.1.10697 20009561

[B29] Calvo-Garrido J. King J. S. Muñoz-Braceras S. Escalante R. (2014). Vmp1 regulates PtdIns3P signaling during autophagosome formation in *Dictyostelium discoideum*. Traffic 15, 1235–1246. 10.1111/tra.12210 25131297

[B30] Cambier C. J. Takaki K. K. Larson R. P. Hernandez R. E. Tobin D. M. Urdahl K. B.. (2014). Mycobacteria manipulate macrophage recruitment through coordinated use of membrane lipids. Nature 505, 218–222. 10.1038/nature12799 24336213 PMC3961847

[B31] Cardenal-Muñoz E. Arafah S. Lopez-Jimenez A. T. Kicka S. Falaise A. Bach F.. (2017). *Mycobacterium marinum* antagonistically induces an autophagic response while repressing the autophagic flux in a TORC1- and ESX-1-dependent manner. PLoS Pathog. 13:e1006344. 10.1371/journal.ppat.1006344 28414774 PMC5407849

[B32] Carlsson F. Joshi S. A. Rangell L. Brown E. J. (2009). Polar localization of virulence-related Esx-1 secretion in mycobacteria. PLoS Pathog. 5:e1000285. 10.1371/journal.ppat.1000285 19180234 PMC2628743

[B33] Chauhan S. Kumar S. Jain A. Ponpuak M. Mudd M. H. Kimura T.. (2016). TRIMs and galectins globally cooperate and TRIM16 and Galectin-3 co-direct autophagy in endomembrane damage homeostasis. Dev. Cell 39, 13–27. 10.1016/j.devcel.2016.08.003 27693506 PMC5104201

[B34] Chawla M. Parikh P. Saxena A. Munshi M. Mehta M. Mai D.. (2012). *Mycobacterium tuberculosis* WhiB4 regulates oxidative stress response to modulate survival and dissemination *in vivo*. Mol. Microbiol. 85, 1148–1165. 10.1111/j.1365-2958.2012.08165.x 22780904 PMC3438311

[B35] Chen Y. Y. Yang F. L. Wu S. H. Lin T. L. Wang J. T. (2015). *Mycobacterium marinum* mmar_2318 and mmar_2319 are responsible for lipooligosaccharide biosynthesis and virulence toward Dictyostelium. Front. Microbiol. 6:1458. 10.3389/fmicb.2015.01458 26779131 PMC4703794

[B36] Clarke M. Köhler J. Arana Q. Liu T. Heuser J. Gerisch G. (2002). Dynamics of the vacuolar H(+)-ATPase in the contractile vacuole complex and the endosomal pathway of Dictyostelium cells. J. Cell. Sci. 115(Pt 14), 2893–2905. 12082150 10.1242/jcs.115.14.2893

[B37] Cole S. T. Brosch R. Parkhill J. Garnier T. Churcher C. Harris D.. (1998). Deciphering the biology of *Mycobacterium tuberculosis* from the complete genome sequence. Nature 393, 537–544. 10.1038/31159 9634230

[B38] Collins C. A. De Mazière A. van Dijk S. Carlsson F. Klumperman J. Brown E. J. (2009). Atg5-independent sequestration of ubiquitinated mycobacteria. PLoS Pathog. 5:e1000430. 10.1371/journal.ppat.1000430 19436699 PMC2673685

[B39] Conrad W. H. Osman M. M. Shanahan J. K. Chu F. Takaki K. K. Cameron J.. (2017). Mycobacterial ESX-1 secretion system mediates host cell lysis through bacterium contact-dependent gross membrane disruptions. Proc. Natl. Acad. Sci. U.S.A. 114, 1371–1376. 10.1073/pnas.1620133114 28119503 PMC5307465

[B40] Cooper D. N. Barondes S. H. (1984). Colocalization of discoidin-binding ligands with discoidin in developing *Dictyostelium discoideum*. Dev. Biol. 105, 59–70. 10.1016/0012-1606(84)90261-6 6468764

[B41] Cooper D. N. Lee S. C. Barondes S. H. (1983). Discoidin-binding polysaccharide from *Dictyostelium discoideum*. J. Biol. Chem. 258, 8745–8750. 6345545

[B42] Cox J. S. Chen B. McNeil M. Jacobs W. R. Jr. (1999). Complex lipid determines tissue-specific replication of *Mycobacterium tuberculosis* in mice. Nature 402, 79–83. 10.1038/47042 10573420

[B43] Daffe M. Laneelle M. A. Lacave C. (1991). Structure and stereochemistry of mycolic acids of *Mycobacterium marinum* and *Mycobacterium ulcerans*. Res. Microbiol. 142, 397–403. 10.1016/0923-2508(91)90109-N 1871424

[B44] D'Avila H. Melo R. C. Parreira G. G. Werneck-Barroso E. H. C. Castro-Faria-Neto Bozza P. T. (2006). *Mycobacterium bovis* bacillus Calmette-Guerin induces TLR2-mediated formation of lipid bodies: intracellular domains for eicosanoid synthesis *in vivo*. J. Immunol. 176, 3087–3097. 10.4049/jimmunol.176.5.3087 16493068

[B45] Day T. A. Mittler J. E. Nixon M. R. Thompson C. Miner M. D. Hickey M. J.. (2014). *Mycobacterium tuberculosis* strains lacking surface lipid phthiocerol dimycocerosate are susceptible to killing by an early innate host response. Infect. Immun. 82, 5214–5222. 10.1128/IAI.01340-13 25287926 PMC4249296

[B46] Decostere A. Hermans K. Haesebrouck F. (2004). Piscine mycobacteriosis: a literature review covering the agent and the disease it causes in fish and humans. Vet. Microbiol. 99, 159–166. 10.1016/j.vetmic.2003.07.011 15066718

[B47] DeJesus M. A. Gerrick E. R. Xu W. Park S. W. Long J. E. Boutte C. C.. (2017a). Comprehensive essentiality analysis of the *Mycobacterium tuberculosis* genome via saturating transposon mutagenesis. MBio 8, e02133-16. 10.1128/mBio.02133-16 28096490 PMC5241402

[B48] DeJesus M. A. Ioerger T. R. (2013). A Hidden Markov Model for identifying essential and growth-defect regions in bacterial genomes from transposon insertion sequencing data. BMC Bioinformatics 14:303. 10.1186/1471-2105-14-303 24103077 PMC3854130

[B49] DeJesus M. A. Nambi S. Smith C. M. Baker R. E. Sassetti C. M. Ioerger T. R. (2017b). Statistical analysis of genetic interactions in Tn-Seq data. Nucleic Acids Res. 45:e93. 10.1093/nar/gkx128 28334803 PMC5499643

[B50] DeJesus M. A. Zhang Y. J. Sassetti C. M. Rubin E. J. Sacchettini J. C. Ioerger T. R. (2013). Bayesian analysis of gene essentiality based on sequencing of transposon insertion libraries. Bioinformatics 29, 695–703. 10.1093/bioinformatics/btt043 23361328 PMC3597147

[B51] de Jonge M. I. Pehau-Arnaudet G. Fretz M. M. Romain F. Bottai D. Brodin P.. (2007). ESAT-6 from *Mycobacterium tuberculosis* dissociates from its putative chaperone CFP-10 under acidic conditions and exhibits membrane-lysing activity. J. Bacteriol. 189, 6028–6034. 10.1128/JB.00469-07 17557817 PMC1952024

[B52] Delincé M. J. Bureau J. B. López-Jiménez A. T. Cosson P. Soldati T. McKinney J. D. (2016). A microfluidic cell-trapping device for single-cell tracking of host-microbe interactions. Lab Chip 16, 3276–3285. 10.1039/C6LC00649C 27425421

[B53] de Mattos K. A. Sarno E. N. Pessolani M. C. Bozza P. T. (2012). Deciphering the contribution of lipid droplets in leprosy: multifunctional organelles with roles in Mycobacterium leprae pathogenesis. Mem. Inst. Oswaldo Cruz 107(Suppl. 1), 156–166. 10.1590/S0074-02762012000900023 23283467

[B54] Derrick S. C. Morris S. L. (2007). The ESAT6 protein of *Mycobacterium tuberculosis* induces apoptosis of macrophages by activating caspase expression. Cell. Microbiol. 9, 1547–1555. 10.1111/j.1462-5822.2007.00892.x 17298391

[B55] dictyBase (2004). dictyBase. Available online at: http://dictybase.org/

[B56] Dionne M. S. Ghori N. Schneider D. S. (2003). *Drosophila melanogaster* is a genetically tractable model host for *Mycobacterium marinum*. Infect. Immun. 71, 3540–3550. 10.1128/IAI.71.6.3540-3550.2003 12761139 PMC155752

[B57] Dominguez-Martin E. Cardenal-Munoz E. King J. S. Soldati T. Coria R. Escalante R. (2017). Methods to monitor and quantify autophagy in the social amoeba *Dictyostelium discoideum*. Cells 6:18. 10.3390/cells6030018 28671610 PMC5617964

[B58] Dormann D. Vasiev B. Weijer C. J. (2000). The control of chemotactic cell movement during *Dictyostelium morphogenesis*. Philos. Trans. R. Soc. Lond. B Biol. Sci. 355, 983–991. 10.1098/rstb.2000.0634 11128992 PMC1692793

[B59] Du F. Edwards K. Shen Z. Sun B. De Lozanne A. Briggs S.. (2008). Regulation of contractile vacuole formation and activity in Dictyostelium. EMBO J. 27, 2064–2076. 10.1038/emboj.2008.131 18636095 PMC2516880

[B60] Du X. Barisch C. Paschke P. Herrfurth C. Bertinetti O. Pawolleck N.. (2013). Dictyostelium lipid droplets host novel proteins. Eukaryot. Cell 12, 1517–1529. 10.1128/EC.00182-13 24036346 PMC3837934

[B61] Duleh S. N. Welch M. D. (2010). WASH and the Arp2/3 complex regulate endosome shape and trafficking. Cytoskeleton 67, 193–206. 10.1002/cm.20437 20175130 PMC2887680

[B62] Dumas F. Haanappel E. (2017). Lipids in infectious diseases - The case of AIDS and tuberculosis. Biochim. Biophys. Acta 1859(9 Pt B), 1636–1647. 10.1016/j.bbamem.2017.05.007 28535936

[B63] Dunn J. D. Bosmani C. Barisch C. Raykov L. Lefrançois L. H. Cardenal-Muñoz E. (2018). Eat prey, live: *Dictyostelium discoideum* as a model for cell-autonomous defenses. Front. Immunol. 8:1906 10.3389/fimmu.2017.01906 PMC5758549 29354124

[B64] Eichinger L. Pachebat J. A. Glockner G. Rajandream M. A. Sucgang R. Berriman M.. (2005). The genome of the social amoeba *Dictyostelium discoideum*. Nature 435, 43–57. 10.1038/nature03481 15875012 PMC1352341

[B65] Eichinger L. Rivero F. (2013). Dictyostelium discoideum Protocols. Methods in Molecular Biology, Vol. 57 Heidelberg: Humana Press; Springer Verlag.

[B66] Eitle E. Keller T. Parish C. R. Parish R. W. (1993). Polysaccharides influence the aggregation of *Dictyostelium discoideum* cells and bind to developmentally regulated cell surface proteins. Exp. Cell Res. 205, 374–382. 10.1006/excr.1993.1100 8482342

[B67] Friedrich N. Hagedorn M. Soldati-Favre D. Soldati T. (2012). Prison break: pathogens' strategies to egress from host cells. Microbiol. Mol. Biol. Rev. 76, 707–720. 10.1128/MMBR.00024-12 23204363 PMC3510522

[B68] Gao L. Y. Guo S. McLaughlin B. Morisaki H. Engel J. N. Brown E. J. (2004). A mycobacterial virulence gene cluster extending RD1 is required for cytolysis, bacterial spreading and ESAT-6 secretion. Mol. Microbiol. 53, 1677–1693. 10.1111/j.1365-2958.2004.04261.x 15341647

[B69] Gerasimova A. Kazakov A. E. Arkin A. P. Dubchak I. Gelfand M. S. (2011). Comparative genomics of the dormancy regulons in mycobacteria. J. Bacteriol. 193, 3446–3452. 10.1128/JB.00179-11 21602344 PMC3133309

[B70] Gerstenmaier L. Pilla R. Herrmann L. Herrmann H. Prado M. Villafano G. J.. (2015). The autophagic machinery ensures nonlytic transmission of mycobacteria. Proc. Natl. Acad. Sci. U.S.A. 112, E687–E692. 10.1073/pnas.1423318112 25646440 PMC4343083

[B71] Gey Van Pittius N. C. Gamieldien J. Hide W. Brown G. D. Siezen R. J. Beyers A. D. (2001). The ESAT-6 gene cluster of *Mycobacterium tuberculosis* and other high G+C Gram-positive bacteria. Genome Biol. 2:RESEARCH0044. 11597336 10.1186/gb-2001-2-10-research0044 PMC57799

[B72] Griffin J. E. Gawronski J. D. Dejesus M. A. Ioerger T. R. Akerley B. J. Sassetti C. M. (2011). High-resolution phenotypic profiling defines genes essential for mycobacterial growth and cholesterol catabolism. PLoS Pathog. 7:e1002251. 10.1371/journal.ppat.1002251 21980284 PMC3182942

[B73] Groschel M. I. Sayes F. Simeone R. Majlessi L. Brosch R. (2016). ESX secretion systems: mycobacterial evolution to counter host immunity. Nat. Rev. Microbiol. 14, 677–691. 10.1038/nrmicro.2016.131 27665717

[B74] Hagedorn M. Rohde K. H. Russell D. G. Soldati T. (2009). Infection by tubercular mycobacteria is spread by nonlytic ejection from their amoeba hosts. Science 323, 1729–1733. 10.1126/science.1169381 19325115 PMC2770343

[B75] Hagedorn M. Soldati T. (2007). Flotillin and RacH modulate the intracellular immunity of Dictyostelium to *Mycobacterium marinum* infection. Cell. Microbiol. 9, 2716–2733. 10.1111/j.1462-5822.2007.00993.x 17587329

[B76] Hammond R. J. Baron V. O. Oravcova K. Lipworth S. Gillespie S. H. (2015). Phenotypic resistance in mycobacteria: is it because I am old or fat that I resist you? J. Antimicrob. Chemother. 70, 2823–2827. 10.1093/jac/dkv178 26163401

[B77] Houben D. Demangel C. van Ingen J. Perez J. Baldeon L. Abdallah A. M.. (2012). ESX-1-mediated translocation to the cytosol controls virulence of mycobacteria. Cell. Microbiol. 14, 1287–1298. 10.1111/j.1462-5822.2012.01799.x 22524898

[B78] Houben E. N. Korotkov K. V. Bitter W. (2014). Take five - Type VII secretion systems of Mycobacteria. Biochim. Biophys. Acta 1843, 1707–1716. 10.1016/j.bbamcr.2013.11.003 24263244

[B79] Hsu T. Hingley-Wilson S. M. Chen B. Chen M. Dai A. Z. Morin P. M.. (2003). The primary mechanism of attenuation of bacillus Calmette-Guerin is a loss of secreted lytic function required for invasion of lung interstitial tissue. Proc. Natl. Acad. Sci. U.S.A. 100, 12420–12425. 10.1073/pnas.1635213100 14557547 PMC218773

[B80] Jackson M. (2014). The mycobacterial cell envelope-lipids. Cold Spring Harb. Perspect. Med. 4:a021105. 10.1101/cshperspect.a021105 25104772 PMC4200213

[B81] Jamwal S. V. Mehrotra P. Singh A. Siddiqui Z. Basu A. Rao K. V. (2016). Mycobacterial escape from macrophage phagosomes to the cytoplasm represents an alternate adaptation mechanism. Sci. Rep. 6:23089. 10.1038/srep23089 26980157 PMC4793295

[B82] Jayachandran R. Sundaramurthy V. Combaluzier B. Mueller P. Korf H. Huygen K.. (2007). Survival of mycobacteria in macrophages is mediated by coronin 1-dependent activation of calcineurin. Cell 130, 37–50. 10.1016/j.cell.2007.04.043 17632055

[B83] Kaku T. Kawamura I. Uchiyama R. Kurenuma T. Mitsuyama M. (2007). RD1 region in mycobacterial genome is involved in the induction of necrosis in infected RAW264 cells via mitochondrial membrane damage and ATP depletion. FEMS Microbiol. Lett. 274, 189–195. 10.1111/j.1574-6968.2007.00838.x 17610510

[B84] Kenyon A. Gavriouchkina D. Zorman J. Napolitani G. Cerundolo V. Sauka-Spengler T. (2017). Active nuclear transcriptome analysis reveals inflammasome-dependent mechanism for early neutrophil response to *Mycobacterium marinum*. Sci. Rep. 7:6505. 10.1038/s41598-017-06099-x 28747644 PMC5529371

[B85] Kicka S. Trofimov V. Harrison C. Ouertatani-Sakouhi H. McKinney J. Scapozza L.. (2014). Establishment and validation of whole-cell based fluorescence assays to identify anti-mycobacterial compounds using the *Acanthamoeba castellanii*-*Mycobacterium marinum* host-pathogen system. PLoS ONE 9:e87834. 10.1371/journal.pone.0087834 24498207 PMC3909256

[B86] Kimura T. Jain A. Choi S. W. Mandell M. A. Schroder K. Johansen T.. (2015). TRIM-mediated precision autophagy targets cytoplasmic regulators of innate immunity. J. Cell Biol. 210, 973–989. 10.1083/jcb.201503023 26347139 PMC4576868

[B87] King J. S. Veltman D. M. Insall R. H. (2011). The induction of autophagy by mechanical stress. Autophagy 7, 1490–1499. 10.4161/auto.7.12.17924 22024750 PMC3327616

[B88] Kinhikar A. G. Verma I. Chandra D. Singh K. K. Weldingh K. Andersen P.. (2010). Potential role for ESAT6 in dissemination of *M. tuberculosis* via human lung epithelial cells. Mol. Microbiol. 75, 92–106. 10.1111/j.1365-2958.2009.06959.x 19906174 PMC2846543

[B89] Kolonko M. Geffken A. C. Blumer T. Hagens K. Schaible U. E. Hagedorn M. (2014). WASH-driven actin polymerization is required for efficient mycobacterial phagosome maturation arrest. Cell. Microbiol. 16, 232–246. 10.1111/cmi.12217 24119059

[B90] Kreibich S. Emmenlauer M. Fredlund J. Rämö P. Munz C. Dehio C.. (2015). Autophagy proteins promote repair of endosomal membranes damaged by the Salmonella type three secretion system 1. Cell Host Microbe 18, 527–537. 10.1016/j.chom.2015.10.015 26567507

[B91] Kuspa A. (2006). Restriction enzyme-mediated integration (REMI) mutagenesis. Methods Mol. Biol. 346, 201–209. 10.1385/1-59745-144-4:201 16957292

[B92] Kuspa A. Loomis W. F. (1992). Tagging developmental genes in Dictyostelium by restriction enzyme-mediated integration of plasmid DNA. Proc. Natl. Acad. Sci. U.S.A. 89, 8803–8807. 10.1073/pnas.89.18.8803 1326764 PMC50009

[B93] Lampe E. O. Brenz Y. Herrmann L. Repnik U. Griffiths G. Zingmark C.. (2015). Dissection of Francisella-host cell interactions in *Dictyostelium discoideum*. Appl. Environ. Microbiol. 82, 1586–1598. 10.1128/AEM.02950-15 26712555 PMC4771330

[B94] Lee W. VanderVen B. C. Fahey R. J. Russell D. G. (2013). Intracellular *Mycobacterium tuberculosis* exploits host-derived fatty acids to limit metabolic stress. J. Biol. Chem. 288, 6788–6800. 10.1074/jbc.M112.445056 23306194 PMC3591590

[B95] Lelong E. Marchetti A. Gueho A. Lima W. C. Sattler N. Molmeret M.. (2011). Role of magnesium and a phagosomal P-type ATPase in intracellular bacterial killing. Cell. Microbiol. 13, 246–258. 10.1111/j.1462-5822.2010.01532.x 21040356

[B96] Lerena M. C. Colombo M. I. (2011). *Mycobacterium marinum* induces a marked LC3 recruitment to its containing phagosome that depends on a functional ESX-1 secretion system. Cell. Microbiol. 13, 814–835. 10.1111/j.1462-5822.2011.01581.x 21447143

[B97] Loomis W. F. (2014). Cell signaling during development of Dictyostelium. Dev. Biol. 391, 1–16. 10.1016/j.ydbio.2014.04.001 24726820 PMC4075484

[B98] Low K. L. Rao P. S. Shui G. Bendt A. K. Pethe K. Dick T.. (2009). Triacylglycerol utilization is required for regrowth of *in vitro* hypoxic nonreplicating *Mycobacterium bovis* bacillus Calmette-Guerin. J. Bacteriol. 191, 5037–5043. 10.1128/JB.00530-09 19525349 PMC2725574

[B99] Madley I. C. Hames B. D. (1981). An analysis of discoidin I binding sites in *Dictyostelium discoideum* (NC4). Biochem. J. 200, 83–91. 10.1042/bj2000083 7036990 PMC1163505

[B100] Mandell M. A. Jain A. Arko-Mensah J. Chauhan S. Kimura T. Dinkins C.. (2014). TRIM proteins regulate autophagy and can target autophagic substrates by direct recognition. Dev. Cell 30, 394–409. 10.1016/j.devcel.2014.06.013 25127057 PMC4146662

[B101] Maniak M. (2011). Dictyostelium as a model for human lysosomal and trafficking diseases. Semin. Cell Dev. Biol. 22, 114–119. 10.1016/j.semcdb.2010.11.001 21056680

[B102] McGourty K. Thurston T. L. Matthews S. A. Pinaud L. Mota L. J. Holden D. W. (2012). Salmonella inhibits retrograde trafficking of mannose-6-phosphate receptors and lysosome function. Science 338, 963–967. 10.1126/science.1227037 23162002 PMC6485626

[B103] McKinney J. D. Höner zu Bentrup K. Muñoz-Elías E. J. Miczak A. Chen B. Chan W. T.. (2000). Persistence of *Mycobacterium tuberculosis* in macrophages and mice requires the glyoxylate shunt enzyme isocitrate lyase. Nature 406, 735–738. 10.1038/35021074 10963599

[B104] Mendum T. A. Wu H. Kierzek A. M. Stewart G. R. (2015). Lipid metabolism and Type VII secretion systems dominate the genome scale virulence profile of *Mycobacterium tuberculosis* in human dendritic cells. BMC Genomics 16:372. 10.1186/s12864-015-1569-2 25956932 PMC4425887

[B105] Mesquita A. Cardenal-Munoz E. Dominguez E. Munoz-Braceras S. Nunez-Corcuera B. Phillips B. A.. (2016). Autophagy in Dictyostelium: mechanisms, regulation and disease in a simple biomedica*l* model. Autophagy 13, 24–40. 10.1080/15548627.2016.1226737 27715405 PMC5240833

[B106] Mesquita A. Tabara L. C. Martinez-Costa O. Santos-Rodrigo N. Vincent O. Escalante R. (2015). Dissecting the function of Atg1 complex in Dictyostelium autophagy reveals a connection with the pentose phosphate pathway enzyme transketolase. Open Biol. 5:150088. 10.1098/rsob.150088 26246495 PMC4554924

[B107] Miranda E. R. Rot G. Toplak M. Santhanam B. Curk T. Shaulsky G.. (2013). Transcriptional profiling of Dictyostelium with RNA sequencing. Methods Mol. Biol. 983, 139–171. 10.1007/978-1-62703-302-2_8 23494306 PMC3892559

[B108] Mukai A. Ichiraku A. Horikawa K. (2016). Reliable handling of highly A/T-rich genomic DNA for efficient generation of knockin strains of *Dictyostelium discoideum*. BMC Biotechnol. 16:37. 10.1186/s12896-016-0267-8 27075750 PMC4831088

[B109] Munoz-Elias E. J. McKinney J. D. (2005). *Mycobacterium tuberculosis* isocitrate lyases 1 and 2 are jointly required for *in vivo* growth and virulence. Nat. Med. 11, 638–644. 10.1038/nm1252 15895072 PMC1464426

[B110] Nakatogawa H. (2015). Regulated degradation: controlling the stability of autophagy gene transcripts. Dev. Cell 34, 132–134. 10.1016/j.devcel.2015.07.002 26218319

[B111] Nalpas N. C. Magee D. A. Conlon K. M. Browne J. A. Healy C. McLoughlin K. E.. (2015). RNA sequencing provides exquisite insight into the manipulation of the alveolar macrophage by tubercle bacilli. Sci. Rep. 5:13629. 10.1038/srep13629 26346536 PMC4642568

[B112] Nambi S. Long J. E. Mishra B. B. Baker R. Murphy K. C. Olive A. J.. (2015). The oxidative stress network of *Mycobacterium tuberculosis* reveals coordination between radical detoxification systems. Cell Host Microbe 17, 829–837. 10.1016/j.chom.2015.05.008 26067605 PMC4465913

[B113] Nazarova E. V. Montague C. R. La T. Wilburn K. M. Sukumar N. Lee W.. (2017). Rv3723/LucA coordinates fatty acid and cholesterol uptake in *Mycobacterium tuberculosis*. Elife 6:e26969. 10.7554/eLife.26969 28708968 PMC5487216

[B114] Nichols J. M. Veltman D. Kay R. R. (2015). Chemotaxis of a model organism: progress with Dictyostelium. Curr. Opin. Cell Biol. 36, 7–12. 10.1016/j.ceb.2015.06.005 26183444

[B115] Noad J. A. von der Malsburg Pathe C. Michel M. A. Komander D. Randow F. (2017). LUBAC-synthesized linear ubiquitin chains restrict cytosol-invading bacteria by activating autophagy and NF-kappaB. Nat. Microbiol. 2:17063. 10.1038/nmicrobiol.2017.63 28481331 PMC5576533

[B116] Otto G. P. Wu M. Y. Kazgan N. Anderson O. R. Kessin R. H. (2004). Dictyostelium macroautophagy mutants vary in the severity of their developmental defects. J. Biol. Chem. 279, 15621–15629. 10.1074/jbc.M311139200 14736886

[B117] Ouertatani-Sakouhi H. Kicka S. Chiriano G. Harrison C. F. Hilbi H. Scapozza L.. (2017). Inhibitors of *Mycobacterium marinum* virulence identified in a *Dictyostelium discoideum* host model. PLoS ONE 12:e0181121. 10.1371/journal.pone.0181121 28727774 PMC5519057

[B118] Pallen M. J. (2002). The ESAT-6/WXG100 superfamily – and a new Gram-positive secretion system? Trends Microbiol. 10, 209–212. 10.1016/S0966-842X(02)02345-4 11973144

[B119] Pandey A. K. Sassetti C. M. (2008). Mycobacterial persistence requires the utilization of host cholesterol. Proc. Natl. Acad. Sci. U.S.A. 105, 4376–4380. 10.1073/pnas.0711159105 18334639 PMC2393810

[B120] Parikka M. Hammaren M. M. Harjula S. K. Halfpenny N. J. Oksanen K. E. Lahtinen M. J.. (2012). *Mycobacterium marinum* causes a latent infection that can be reactivated by gamma irradiation in adult zebrafish. PLoS Pathog. 8:e1002944. 10.1371/journal.ppat.1002944 23028333 PMC3459992

[B121] Patterson D. J. (1980). Contractile vacuoles and associated structures: their organization and function. Biol. Rev. 55, 1–46. 10.1111/j.1469-185X.1980.tb00686.x

[B122] Pean C. B. Schiebler M. Tan S. W. Sharrock J. A. Kierdorf K. Brown K. P.. (2017). Regulation of phagocyte triglyceride by a STAT-ATG2 pathway controls mycobacterial infection. Nat. Commun. 8:14642. 10.1038/ncomms14642 28262681 PMC5343520

[B123] Pethe K. Swenson D. L. Alonso S. Anderson J. Wang C. Russell D. G. (2004). Isolation of *Mycobacterium tuberculosis* mutants defective in the arrest of phagosome maturation. Proc. Natl. Acad. Sci. U.S.A. 101, 13642–13647. 10.1073/pnas.0401657101 15340136 PMC518761

[B124] Pettersson B. M. Das S. Behra P. R. Jordan H. R. Ramesh M. Mallick A.. (2015). Comparative sigma factor-mRNA levels in *Mycobacterium marinum* under stress conditions and during host infection. PLoS ONE 10:e0139823. 10.1371/journal.pone.0139823 26445268 PMC4596819

[B125] Peyron P. Vaubourgeix J. Poquet Y. Levillain F. Botanch C. Bardou F.. (2008). Foamy macrophages from tuberculous patients' granulomas constitute a nutrient-rich reservoir for *M. tuberculosis* persistence. PLoS Pathog 4:e1000204. 10.1371/journal.ppat.1000204 19002241 PMC2575403

[B126] Pym A. S. Brodin P. Brosch R. Huerre M. Cole S. T. (2002). Loss of RD1 contributed to the attenuation of the live tuberculosis vaccines *Mycobacterium bovis* BCG and *Mycobacterium microti*. Mol. Microbiol. 46, 709–717. 10.1046/j.1365-2958.2002.03237.x 12410828

[B127] Quigley J. Hughitt V. K. Velikovsky C. A. Mariuzza R. A. El-Sayed N. M. Briken V. (2017). The cell wall lipid PDIM contributes to phagosomal escape and host cell exit of *Mycobacterium tuberculosis*. MBio 8:e00148-17. 10.1128/mBio.00148-17 28270579 PMC5340868

[B128] Ramakrishnan L. (2004). Using *Mycobacterium marinum* and its hosts to study tuberculosis. Curr. Sci. 86, 82–92. Available online at: https://iths.pure.elsevier.com/en/publications/using-mycobacterium-marinum-and-its-hosts-to-study-tuberculosis

[B129] Ramakrishnan L. Federspiel N. A. Falkow S. (2000). Granuloma-specific expression of Mycobacterium virulence proteins from the glycine-rich PE-PGRS family. Science 288, 1436–1439. 10.1126/science.288.5470.1436 10827956

[B130] Raper K. B. (1935). *Dictyostelium discoideum*, a new species of slime mold from decaying forest leaves. J. Agric. Res. 50, 135–147.

[B131] Renshaw P. S. Lightbody K. L. Veverka V. Muskett F. W. Kelly G. Frenkiel T. A.. (2005). Structure and function of the complex formed by the tuberculosis virulence factors CFP-10 and ESAT-6. EMBO J. 24, 2491–2498. 10.1038/sj.emboj.7600732 15973432 PMC1176459

[B132] Romagnoli A. Etna M. P. Giacomini E. Pardini M. Remoli M. E. Corazzari M.. (2012). ESX-1 dependent impairment of autophagic flux by *Mycobacterium tuberculosis* in human dendritic cells. Autophagy 8, 1357–1370. 10.4161/auto.20881 22885411 PMC3442882

[B133] Rosel D. Khurana T. Majithia A. Huang X. Bhandari R. Kimmel A. R. (2012). TOR complex 2 (TORC2) in Dictyostelium suppresses phagocytic nutrient capture independently of TORC1-mediated nutrient sensing. J. Cell. Sci. 125(Pt 1), 37–48. 10.1242/jcs.077040 22266904 PMC3269021

[B134] Rosen S. D. Kafka J. A. Simpson D. L. Barondes S. H. (1973). Developmentally regulated, carbohydrate-binding protein in *Dictyostelium discoideum*. Proc. Natl. Acad. Sci. U.S.A. 70, 2554–2557. 10.1073/pnas.70.9.2554 4517669 PMC427054

[B135] Rosengarten R. D. Santhanam B. Fuller D. Katoh-Kurasawa M. Loomis W. F. Zupan B.. (2015). Leaps and lulls in the developmental transcriptome of *Dictyostelium discoideum*. BMC Genomics 16:294. 10.1186/s12864-015-1491-7 25887420 PMC4403905

[B136] Russell D. G. Cardona P. J. Kim M. J. Allain S. Altare F. (2009). Foamy macrophages and the progression of the human tuberculosis granuloma. Nat. Immunol. 10, 943–948. 10.1038/ni.1781 19692995 PMC2759071

[B137] Ryu Y. J. Koh W. J. Daley C. L. (2016). Diagnosis and treatment of nontuberculous mycobacterial lung disease: clinicians' perspectives. Tuberc. Respir. Dis. 79, 74–84. 10.4046/trd.2016.79.2.74 27066084 PMC4823187

[B138] Saliba A. E. Santos S. Vogel J. (2017). New RNA-seq approaches for the study of bacterial pathogens. Curr. Opin. Microbiol. 35, 78–87. 10.1016/j.mib.2017.01.001 28214646

[B139] Sattler N. Monroy R. Soldati T. (2013). Quantitative analysis of phagocytosis and phagosome maturation. Methods Mol. Biol. 983, 383–402. 10.1007/978-1-62703-302-2_21 23494319

[B140] Schiestl R. H. Petes T. D. (1991). Integration of DNA fragments by illegitimate recombination in Saccharomyces cerevisiae. Proc. Natl. Acad. Sci. U.S.A. 88, 7585–7589. 10.1073/pnas.88.17.7585 1881899 PMC52346

[B141] Schmidt O. Teis D. (2012). The ESCRT machinery. Curr. Biol. 22, R116–R120. 10.1016/j.cub.2012.01.028 22361144 PMC3314914

[B142] Schnappinger D. Ehrt S. Voskuil M. I. Liu Y. Mangan J. A. Monahan I. M.. (2003). Transcriptional adaptation of *Mycobacterium tuberculosis* within macrophages: insights into the phagosomal environment. J. Exp. Med. 198, 693–704. 10.1084/jem.20030846 12953091 PMC2194186

[B143] Schulze R. J. Sathyanarayan A. Mashek D. G. (2017). Breaking fat: the regulation and mechanisms of lipophagy. Biochim. Biophys. Acta 1862(10 Pt B), 1178–1187. 10.1016/j.bbalip.2017.06.008 28642194 PMC5595645

[B144] Senaratne R. H. Sidders B. Sequeira P. Saunders G. Dunphy K. Marjanovic O.. (2008). *Mycobacterium tuberculosis* strains disrupted in mce3 and mce4 operons are attenuated in mice. J. Med. Microbiol. 57(Pt 2), 164–170. 10.1099/jmm.0.47454-0 18201981

[B145] Serafini A. Boldrin F. Palu G. Manganelli R. (2009). Characterization of a *Mycobacterium tuberculosis* ESX-3 conditional mutant: essentiality and rescue by iron and zinc. J. Bacteriol. 191, 6340–6344. 10.1128/JB.00756-09 19684129 PMC2753049

[B146] Serafini A. Pisu D. Palu G. Rodriguez G. M. Manganelli R. (2013). The ESX-3 secretion system is necessary for iron and zinc homeostasis in *Mycobacterium tuberculosis*. PLoS ONE 8:e78351. 10.1371/journal.pone.0078351 24155985 PMC3796483

[B147] Shevchuk O. Batzilla C. Hagele S. Kusch H. Engelmann S. Hecker M.. (2009). Proteomic analysis of Legionella-containing phagosomes isolated from Dictyostelium. Int. J. Med. Microbiol. 299, 489–508. 10.1016/j.ijmm.2009.03.006 19482547

[B148] Siegrist M. S. Steigedal M. Ahmad R. Mehra A. Dragset M. S. Schuster B. M.. (2014). Mycobacterial Esx-3 requires multiple components for iron acquisition. MBio 5, e01073–e01014. 10.1128/mBio.01073-14 24803520 PMC4010830

[B149] Siegrist M. S. Unnikrishnan M. McConnell M. J. Borowsky M. Cheng T. Y. Siddiqi N.. (2009). Mycobacterial Esx-3 is required for mycobactin-mediated iron acquisition. Proc. Natl. Acad. Sci. U.S.A. 106, 18792–18797. 10.1073/pnas.0900589106 19846780 PMC2774023

[B150] Simeone R. Bobard A. Lippmann J. Bitter W. Majlessi L. Brosch R.. (2012). Phagosomal rupture by *Mycobacterium tuberculosis* results in toxicity and host cell death. PLoS Pathog. 8:e1002507. 10.1371/journal.ppat.1002507 22319448 PMC3271072

[B151] Simeone R. Sayes F. Song O. Groschel M. I. Brodin P. Brosch R.. (2015). Cytosolic access of *Mycobacterium tuberculosis*: critical impact of phagosomal acidification control and demonstration of occurrence *in vivo*. PLoS Pathog. 11:e1004650. 10.1371/journal.ppat.1004650 25658322 PMC4450080

[B152] Singh K. H. Jha B. Dwivedy A. Choudhary E. AG N. Ashraf A.. (2017). Characterization of a secretory hydrolase from *Mycobacterium tuberculosis* sheds critical insight into host lipid utilization by *M*. tuberculosis. J. Biol. Chem. 292, 11326–11335. 10.1074/jbc.M117.794297 28515317 PMC5500798

[B153] Smith E. W. Lima W. C. Charette S. J. Cosson P. (2010). Effect of starvation on the endocytic pathway in Dictyostelium cells. Eukaryot. Cell 9, 387–392. 10.1128/EC.00285-09 20097741 PMC2837978

[B154] Solomon J. M. Leung G. S. Isberg R. R. (2003). Intracellular replication of *Mycobacterium marinum* within *Dictyostelium discoideum*: efficient replication in the absence of host coronin. Infect. Immun. 71, 3578–3586. 10.1128/IAI.71.6.3578-3586.2003 12761143 PMC155778

[B155] Sorensen A. L. Nagai S. Houen G. Andersen P. Andersen A. B. (1995). Purification and characterization of a low-molecular-mass T-cell antigen secreted by *Mycobacterium tuberculosis*. Infect. Immun. 63, 1710–1717. 7729876 10.1128/iai.63.5.1710-1717.1995 PMC173214

[B156] Srinivasan L. Ahlbrand S. Briken V. (2014). Interaction of *Mycobacterium tuberculosis* with host cell death pathways. Cold Spring Harb. Perspect. Med. 4:a022459. 10.1101/cshperspect.a022459 24968864 PMC4109581

[B157] Stamm L. M. Morisaki J. H. Gao L. Y. Jeng R. L. McDonald K. L. Roth R.. (2003). *Mycobacterium marinum* escapes from phagosomes and is propelled by actin-based motility. J. Exp. Med. 198, 1361–1368. 10.1084/jem.20031072 14597736 PMC2194249

[B158] Steiner B. Weber S. Hilbi H. (2017). Formation of the Legionella-containing vacuole: phosphoinositide conversion, GTPase modulation and ER dynamics. Int. J. Med. Microbiol. 10.1016/j.ijmm.2017.08.004 28865995

[B159] Stevense M. Chubb J. R. Muramoto T. (2011). Nuclear organization and transcriptional dynamics in Dictyostelium. Dev. Growth Differ. 53, 576–586. 10.1111/j.1440-169X.2011.01271.x 21585360

[B160] Stewart G. R. Patel J. Robertson B. D. Rae A. Young D. B. (2005). Mycobacterial mutants with defective control of phagosomal acidification. PLoS Pathog. 1, 269–278. 10.1371/journal.ppat.0010033 16322769 PMC1291353

[B161] Stinear T. P. Jenkin G. A. Johnson P. D. Davies J. K. (2000). Comparative genetic analysis of *Mycobacterium ulcerans* and *Mycobacterium marinum* reveals evidence of recent divergence. J. Bacteriol. 182, 6322–6330. 10.1128/JB.182.22.6322-6330.2000 11053375 PMC94777

[B162] Stinear T. P. Seemann T. Harrison P. F. Jenkin G. A. Davies J. K. Johnson P. D.. (2008). Insights from the complete genome sequence of *Mycobacterium marinum* on the evolution of *Mycobacterium tuberculosis*. Genome Res. 18, 729–741. 10.1101/gr.075069.107 18403782 PMC2336800

[B163] Tan T. Lee W. L. Alexander D. C. Grinstein S. Liu J. (2006). The ESAT-6/CFP-10 secretion system of *Mycobacterium marinum* modulates phagosome maturation. Cell. Microbiol. 8, 1417–1429. 10.1111/j.1462-5822.2006.00721.x 16922861

[B164] Tattoli I. Philpott D. J. Girardin S. E. (2012a). The bacterial and cellular determinants controlling the recruitment of mTOR to the Salmonella-containing vacuole. Biol. Open 1, 1215–1225. 10.1242/bio.20122840 23259056 PMC3522883

[B165] Tattoli I. Sorbara M. T. Vuckovic D. Ling A. Soares F. Carneiro L. A.. (2012b). Amino acid starvation induced by invasive bacterial pathogens triggers an innate host defense program. Cell Host Microbe 11, 563–575. 10.1016/j.chom.2012.04.012 22704617

[B166] Thiam A. R. Farese R. V. Jr. Walther T. C. (2013). The biophysics and cell biology of lipid droplets. Nat. Rev. Mol. Cell Biol. 14, 775–786. 10.1038/nrm3699 24220094 PMC4526153

[B167] Thurston T. L. Boyle K. B. Allen M. Ravenhill B. J. Karpiyevich M. Bloor S.. (2016). Recruitment of TBK1 to cytosol-invading Salmonella induces WIPI2-dependent antibacterial autophagy. EMBO J. 35, 1779–1792. 10.15252/embj.201694491 27370208 PMC5010046

[B168] Thurston T. L. Ryzhakov G. Bloor S. von Muhlinen N. Randow F. (2009). The TBK1 adaptor and autophagy receptor NDP52 restricts the proliferation of ubiquitin-coated bacteria. Nat. Immunol. 10, 1215–1221. 10.1038/ni.1800 19820708

[B169] Tobin D. M. Ramakrishnan L. (2008). Comparative pathogenesis of *Mycobacterium marinum* and *Mycobacterium tuberculosis*. Cell. Microbiol. 10, 1027–1039. 10.1111/j.1462-5822.2008.01133.x 18298637

[B170] Tonjum T. Welty D. B. Jantzen E. Small P. L. (1998). Differentiation of *Mycobacterium ulcerans, M. marinum*, and *M. haemophilum*: mapping of their relationships to *M. tuberculosis* by fatty acid profile analysis, DNA-DNA hybridization, and 16S rRNA gene sequence analysis. J. Clin. Microbiol. 36, 918–925. 9542909 10.1128/jcm.36.4.918-925.1998 PMC104661

[B171] Tosetti N. Croxatto A. Greub G. (2014). Amoebae as a tool to isolate new bacterial species, to discover new virulence factors and to study the host-pathogen interactions. Microb. Pathog. 77, 125–130. 10.1016/j.micpath.2014.07.009 25088032

[B172] Trofimov V. Kicka S. Mucaria S. Hanna N. Ramon-Olayo F. Vela-González Del Peral L. (in press). Phenotypic screening of antimycobacterial compounds in alternative infection models identifies anti-infectives their molecular targets in cell wall-related pathways. Sci. Rep. 10.1038/s41598-018-22228-6 PMC5834492 29500372

[B173] Unnikrishnan M. Constantinidou C. Palmer T. Pallen M. J. (2017). The enigmatic Esx proteins: looking beyond Mycobacteria. Trends Microbiol. 25, 192–204. 10.1016/j.tim.2016.11.004 27894646

[B174] Urwyler S. Nyfeler Y. Ragaz C. Lee H. Mueller L. N. Aebersold R.. (2009). Proteome analysis of Legionella vacuoles purified by magnetic immunoseparation reveals secretory and endosomal GTPases. Traffic 10, 76–87. 10.1111/j.1600-0854.2008.00851.x 18980612

[B175] van Opijnen T. Bodi K. L. Camilli A. (2009). Tn-seq: high-throughput parallel sequencing for fitness and genetic interaction studies in microorganisms. Nat. Methods 6, 767–772. 10.1038/nmeth.1377 19767758 PMC2957483

[B176] van Opijnen T. Camilli A. (2013). Transposon insertion sequencing: a new tool for systems-level analysis of microorganisms. Nat. Rev. Microbiol. 11, 435–442. 10.1038/nrmicro3033 23712350 PMC3842022

[B177] Veltman D. M. Akar G. Bosgraaf L. Van Haastert P. J. (2009a). A new set of small, extrachromosomal expression vectors for *Dictyostelium discoideum*. Plasmid 61, 110–118. 10.1016/j.plasmid.2008.11.003 19063918

[B178] Veltman D. M. Keizer-Gunnink I. Haastert P. J. (2009b). An extrachromosomal, inducible expression system for *Dictyostelium discoideum*. Plasmid 61, 119–125. 10.1016/j.plasmid.2008.11.002 19046986

[B179] Volkman H. E. Clay H. Beery D. Chang J. C. Sherman D. R. Ramakrishnan L. (2004). Tuberculous granuloma formation is enhanced by a mycobacterium virulence determinant. PLoS Biol. 2:e367. 10.1371/journal.pbio.0020367 15510227 PMC524251

[B180] Wang Q. Zhu L. Jones V. Wang C. Hua Y. Shi X.. (2015). CpsA, a LytR-CpsA-Psr family protein in *Mycobacterium marinum*, is required for cell wall integrity and virulence. Infect. Immun. 83, 2844–2854. 10.1128/IAI.03081-14 25939506 PMC4468561

[B181] Wang S. Dong X. Zhu Y. Wang C. Sun G. Luo T.. (2013). Revealing of *Mycobacterium marinum* transcriptome by RNA-seq. PLoS ONE 8:e75828. 10.1371/journal.pone.0075828 24098731 PMC3786904

[B182] Watts D. J. Ashworth J. M. (1970). Growth of myxameobae of the cellular slime mould *Dictyostelium discoideum* in axenic culture. Biochem. J. 119, 171–174. 10.1042/bj1190171 5530748 PMC1179339

[B183] Weber S. Hilbi H. (2014). Live cell imaging of phosphoinositide dynamics during Legionella infection. Methods Mol. Biol. 1197, 153–167. 10.1007/978-1-4939-1261-2_9 25172280

[B184] Weerdenburg E. M. Abdallah A. M. Rangkuti F. Abd El Ghany M. Otto T. D. Adroub S. A.. (2015). Genome-wide transposon mutagenesis indicates that *Mycobacterium marinum* customizes its virulence mechanisms for survival and replication in different hosts. Infect. Immun. 83, 1778–1788. 10.1128/IAI.03050-14 25690095 PMC4399070

[B185] Westermann A. J. Barquist L. Vogel J. (2017). Resolving host-pathogen interactions by dual RNA-seq. PLoS Pathog. 13:e1006033. 10.1371/journal.ppat.1006033 28207848 PMC5313147

[B186] Wiegand S. Kruse J. Gronemann S. Hammann C. (2011). Efficient generation of gene knockout plasmids for *Dictyostelium discoideum* using one-step cloning. Genomics 97, 321–325. 10.1016/j.ygeno.2011.02.001 21316445

[B187] Williams J. G. (2006). Transcriptional regulation of Dictyostelium pattern formation. EMBO Rep. 7, 694–698. 10.1038/sj.embor.7400714 16819464 PMC1500839

[B188] World Health Organization (2015). Global Tuberculosis Report 2015. Available online at: http://apps.who.int/iris/bitstream/10665/191102/1/9789241565059_eng.pdf

[B189] Wu J. Ru H. W. Xiang Z. H. Jiang J. Wang Y. C. Zhang L.. (2017). WhiB4 Regulates the PE/PPE gene family and is essential for virulence of *Mycobacterium marinum*. Sci. Rep. 7:3007. 10.1038/s41598-017-03020-4 28592799 PMC5462746

[B190] Wullschleger S. Loewith R. Hall M. N. (2006). TOR signaling in growth and metabolism. Cell 124, 471–484. 10.1016/j.cell.2006.01.016 16469695

[B191] Yu J. Tran V. Li M. Huang X. Niu C. Wang D.. (2012). Both phthiocerol dimycocerosates and phenolic glycolipids are required for virulence of *Mycobacterium marinum*. Infect. Immun. 80, 1381–1389. 10.1128/IAI.06370-11 22290144 PMC3318414

[B192] Zhang M. Chen L. Wang S. Wang T. (2009). Rab7: roles in membrane trafficking and disease. Biosci. Rep. 29, 193–209. 10.1042/BSR20090032 19392663

[B193] Zhang Q. Wang D. Jiang G. Liu W. Deng Q. Li X.. (2016). EsxA membrane-permeabilizing activity plays a key role in mycobacterial cytosolic translocation and virulence: effects of single-residue mutations at glutamine 5. Sci. Rep. 6:32618. 10.1038/srep32618 27600772 PMC5013644

[B194] Zhang Y. J. Ioerger T. R. Huttenhower C. Long J. E. Sassetti C. M. Sacchettini J. C.. (2012). Global assessment of genomic regions required for growth in *Mycobacterium tuberculosis*. PLoS Pathog. 8:e1002946. 10.1371/journal.ppat.1002946 23028335 PMC3460630

[B195] Zimmermann M. Kogadeeva M. Gengenbacher M. McEwen G. Mollenkopf H. J. Zamboni N.. (2017). Integration of metabolomics and transcriptomics reveals a complex diet of *Mycobacterium tuberculosis* during early macrophage infection. mSystems 2:e00057-17. 10.1128/mSystems.00057-17 28845460 PMC5566787

