# Supplementary material for: When Dicty Met Myco, a (Not So) Romantic Story about One Amoeba and Its Intracellular Pathogen
Source: Front Cell Infect Microbiol. 2018 Jan 9;7:529. doi: 10.3389/fcimb.2017.00529 (PMC5767268; doi:10.3389/fcimb.2017.00529)
Supplement: Supplementary file 1 [file Table1.docx]

Supplementary Material

When Dicty met Myco, a (not so) Romantic Story about one Amoeba and its Intracellular Pathogen

Elena Cardenal-Muñoz^*^, Caroline Barisch, Louise Lefrançois, Ana Teresa López-Jiménez, Thierry Soldati

*** Correspondence:** Dr Elena Cardenal-Muñoz: elena.cardenal@unige.ch

# Supplementary Tables

**Supplementary Table 1. *M. marinum* markers examined during infection of *D. discoideum*.** *It needs to be noticed that these antibodies might not always recognize *M. marinum*, since its cell wall might be shed during infection, as observed in macrophages (Collins et al. 2009) and/or it might be extracted upon methanol fixation in IFA.

| **Marker** | **Description** | **Experimental purpose** |
| --- | --- | --- |
| Alexa Fluor 488 Hydrazide | Dye labelling aldehydes or ketones in polysaccharides or glycoproteins. It can be used to label *M. marinum* (Sattler, Monroy, and Soldati 2013) | FACS (Sattler, Monroy, and Soldati 2013) |
| anti-ESAT-6 | Mouse HYB076-08 monoclonal antibody | Immunoblot (Hagedorn et al. 2009) |
| anti-mar* | Rabbit anti-*M. marinum* serum (Hagedorn et al. 2009) | IFA (Hagedorn et al. 2009) |
| anti-MLSA-LAM, anti-MLMA-LAM, anti-MLCwA-LAM antibody cocktail* | Rabbit antibodies directed against the *M. leprae* cell wall [Biodefense and Emerging Infections Research (BEI) Resources  Repository, American Type Culture Collection (ATCC)]. It can be used to recognise *M. marinum* (Barisch et al. 2015) | IFA (Barisch et al. 2015) |
| AuramineO | TB Fluorescent Stain Kit from BD. It stains the mycobacterial cell wall in growing non-dormant *M. marinum* (Barisch and Soldati 2017) | IFA (Barisch and Soldati 2017) |
| Bodipy 493/503 | Neutral lipids dye. It stains ILIs in *M. marinum* (Barisch and Soldati 2017) | IFA (Barisch and Soldati 2017; Barisch et al. 2015), Live microscopy (Barisch and Soldati 2017; Barisch et al. 2015) |
| LD540 | Neutral lipids dye. It stains ILIs in *M. marinum* (Barisch and Soldati 2017). Not commercial (Spandl et al. 2009) | IFA (Barisch and Soldati 2017) |
| *map24*::GFP | GFP expression given by the *mag24-1* promoter | IFA (Hagedorn and Soldati 2007) |
| pCherry3 | mCherry codon-optimised for expression in Mtb and cloned in pSMT3-S (expression under control of the engineered *M. smegmatis* Psmyc promoter), Hyg^r^ (Zelmer et al. 2012) | CLEM (Gerstenmaier et al. 2015), IFA (Kolonko et al. 2014; Gerstenmaier et al. 2015), Live microscopy (Kolonko et al. 2014) |
| pCherry10 | mCherry codon-optimised for expression in Mtb and cloned in pSMT3-G13 (expression under control of the *M. marinum* G13 promoter), Hyg^r^ (Zelmer et al. 2012) | Fluorescence recording in microplate reader (Arafah et al. 2013), IFA (Cardenal-Munoz et al. 2017), Immunoblot (Cardenal-Munoz et al. 2017), InfectChip (Delince et al. 2016), Live microscopy (Cardenal-Munoz et al. 2017; Barisch, Lopez-Jimenez, and Soldati 2015) |
| pMm*2318*::pMN437 | *MMAR_2318* under control of its predicted promoter, Hyg^r^. It complements the *∆2318* mutation (Chen et al. 2015) | Plaque assay (Chen et al. 2015) |
| pMm*2319*::pMN437 | *MMAR_2319* under control of the *M. bovis* BCG *hsp60* promoter, Hyg^r^. It complements the *∆2319* mutation (Chen et al. 2015) | Plaque assay (Chen et al. 2015) |
| pMSP12::DsRed2/GFP | DsRed2/GFP under control of the *Mycobacterium* strong promoter (MSP), Kan^r^ (Cosma, Humbert, and Ramakrishnan 2004) | CFUs counting (Hagedorn and Soldati 2007; Arafah et al. 2013), FACS (Hagedorn and Soldati 2007; Arafah et al. 2013; Lelong et al. 2011; Sattler, Monroy, and Soldati 2013; Kolonko et al. 2014; Gerstenmaier et al. 2015), IFA (Hagedorn and Soldati 2007; Hagedorn et al. 2009; Lelong et al. 2011; Kolonko et al. 2014; Gerstenmaier et al. 2015), Immunoblot (Cardenal-Munoz et al. 2017), Live microscopy (Solomon, Leung, and Isberg 2003; Cardenal-Munoz et al. 2017; Hagedorn et al. 2009; Kolonko et al. 2014; Gerstenmaier et al. 2015) |
| pMV261-*tesA* | *tesA* under control of the constitutive *hsp60* promoter. It complements the *tesA*::Tn mutation (Alibaud et al. 2011) | Plaque assay (Alibaud et al. 2011) |
| pMV306::*lux* | bacterial luciferase under control of the G13 promoter, Kan^r^ | Luminescence recording in microplate reader (Arafah et al. 2013; Cardenal-Munoz et al. 2017; Ouertatani-Sakouhi et al. 2017) |
| pR2Hyg | Derived from pMSP12::DsRed2 (Addgene) | Fixed fluorescence microscopy (Hagedorn et al. 2009), IFA (Hagedorn et al. 2009), Live microscopy (Hagedorn et al. 2009) |
| pRD1-2F9 | *Mtb* extended RD1 locus, Hyg^r^ (Pym et al. 2002). It complements the ∆RD1 mutation (Cardenal-Munoz et al. 2017) | Live microscopy (Cardenal-Munoz et al. 2017) |
| Vybrant DyeCycle Ruby | DNA-dye. It can be used for bacteria staining | Live microscopy (Barisch et al. 2015; Barisch and Soldati 2017; Cardenal-Munoz et al. 2017) |

# Supplementary references

Alibaud, L., Y. Rombouts, X. Trivelli, A. Burguiere, S. L. Cirillo, J. D. Cirillo, J. F. Dubremetz, Y. Guerardel, G. Lutfalla, and L. Kremer. 2011. 'A Mycobacterium marinum TesA mutant defective for major cell wall-associated lipids is highly attenuated in Dictyostelium discoideum and zebrafish embryos', *Mol Microbiol*, 80: 919-34.

Arafah, S., S. Kicka, V. Trofimov, M. Hagedorn, N. Andreu, S. Wiles, B. Robertson, and T. Soldati. 2013. 'Setting up and monitoring an infection of Dictyostelium discoideum with mycobacteria', *Methods Mol Biol*, 983: 403-17.

Barisch, C., A. T. Lopez-Jimenez, and T. Soldati. 2015. 'Live imaging of Mycobacterium marinum infection in Dictyostelium discoideum', *Methods Mol Biol*, 1285: 369-85.

Barisch, C., P. Paschke, M. Hagedorn, M. Maniak, and T. Soldati. 2015. 'Lipid droplet dynamics at early stages of Mycobacterium marinum infection in Dictyostelium', *Cell Microbiol*, 17: 1332-49.

Barisch, C., and T. Soldati. 2017. 'Mycobacterium marinum Degrades Both Triacylglycerols and Phospholipids from Its Dictyostelium Host to Synthesise Its Own Triacylglycerols and Generate Lipid Inclusions', *PLoS Pathog*, 13: e1006095.

Cardenal-Munoz, E., S. Arafah, A. T. Lopez-Jimenez, S. Kicka, A. Falaise, F. Bach, O. Schaad, J. S. King, M. Hagedorn, and T. Soldati. 2017. 'Mycobacterium marinum antagonistically induces an autophagic response while repressing the autophagic flux in a TORC1- and ESX-1-dependent manner', *PLoS Pathog*, 13: e1006344.

Chen, Y. Y., F. L. Yang, S. H. Wu, T. L. Lin, and J. T. Wang. 2015. 'Mycobacterium marinum mmar_2318 and mmar_2319 are Responsible for Lipooligosaccharide Biosynthesis and Virulence Toward Dictyostelium', *Front Microbiol*, 6: 1458.

Collins, C. A., A. De Maziere, S. van Dijk, F. Carlsson, J. Klumperman, and E. J. Brown. 2009. 'Atg5-independent sequestration of ubiquitinated mycobacteria', *PLoS Pathog*, 5: e1000430.

Cosma, C. L., O. Humbert, and L. Ramakrishnan. 2004. 'Superinfecting mycobacteria home to established tuberculous granulomas', *Nat Immunol*, 5: 828-35.

Delince, M. J., J. B. Bureau, A. T. Lopez-Jimenez, P. Cosson, T. Soldati, and J. D. McKinney. 2016. 'A microfluidic cell-trapping device for single-cell tracking of host-microbe interactions', *Lab Chip*, 16: 3276-85.

Gerstenmaier, L., R. Pilla, L. Herrmann, H. Herrmann, M. Prado, G. J. Villafano, M. Kolonko, R. Reimer, T. Soldati, J. S. King, and M. Hagedorn. 2015. 'The autophagic machinery ensures nonlytic transmission of mycobacteria', *Proc Natl Acad Sci U S A*, 112: E687-92.

Hagedorn, M., K. H. Rohde, D. G. Russell, and T. Soldati. 2009. 'Infection by tubercular mycobacteria is spread by nonlytic ejection from their amoeba hosts', *Science*, 323: 1729-33.

Hagedorn, M., and T. Soldati. 2007. 'Flotillin and RacH modulate the intracellular immunity of Dictyostelium to Mycobacterium marinum infection', *Cell Microbiol*, 9: 2716-33.

Kolonko, M., A. C. Geffken, T. Blumer, K. Hagens, U. E. Schaible, and M. Hagedorn. 2014. 'WASH-driven actin polymerization is required for efficient mycobacterial phagosome maturation arrest', *Cell Microbiol*, 16: 232-46.

Lelong, E., A. Marchetti, A. Gueho, W. C. Lima, N. Sattler, M. Molmeret, M. Hagedorn, T. Soldati, and P. Cosson. 2011. 'Role of magnesium and a phagosomal P-type ATPase in intracellular bacterial killing', *Cell Microbiol*, 13: 246-58.

Ouertatani-Sakouhi, H., S. Kicka, G. Chiriano, C. F. Harrison, H. Hilbi, L. Scapozza, T. Soldati, and P. Cosson. 2017. 'Inhibitors of Mycobacterium marinum virulence identified in a Dictyostelium discoideum host model', *PLoS One*, 12: e0181121.

Pym, A. S., P. Brodin, R. Brosch, M. Huerre, and S. T. Cole. 2002. 'Loss of RD1 contributed to the attenuation of the live tuberculosis vaccines Mycobacterium bovis BCG and Mycobacterium microti', *Mol Microbiol*, 46: 709-17.

Sattler, N., R. Monroy, and T. Soldati. 2013. 'Quantitative analysis of phagocytosis and phagosome maturation', *Methods Mol Biol*, 983: 383-402.

Solomon, J. M., G. S. Leung, and R. R. Isberg. 2003. 'Intracellular replication of Mycobacterium marinum within Dictyostelium discoideum: efficient replication in the absence of host coronin', *Infect Immun*, 71: 3578-86.

Spandl, J., D. J. White, J. Peychl, and C. Thiele. 2009. 'Live cell multicolor imaging of lipid droplets with a new dye, LD540', *Traffic*, 10: 1579-84.

Zelmer, A., P. Carroll, N. Andreu, K. Hagens, J. Mahlo, N. Redinger, B. D. Robertson, S. Wiles, T. H. Ward, T. Parish, J. Ripoll, G. J. Bancroft, and U. E. Schaible. 2012. 'A new in vivo model to test anti-tuberculosis drugs using fluorescence imaging', *J Antimicrob Chemother*, 67: 1948-60.
