# Supplementary material for: When Dicty Met Myco, a (Not So) Romantic Story about One Amoeba and Its Intracellular Pathogen
Source: Front Cell Infect Microbiol. 2018 Jan 9;7:529. doi: 10.3389/fcimb.2017.00529 (PMC5767268; doi:10.3389/fcimb.2017.00529)
Supplement: Supplementary file 2 [file Table2.docx]

Supplementary Material

When Dicty met Myco, a (not so) Romantic Story about one Amoeba and its Intracellular Pathogen

Elena Cardenal-Muñoz^*^, Caroline Barisch, Louise Lefrançois, Ana Teresa López-Jiménez, Thierry Soldati

*** Correspondence:** Dr Elena Cardenal-Muñoz: elena.cardenal@unige.ch

# Supplementary Tables

**Supplementary Table 2. *M. marinum* strains used to infect *D. discoideum*.** SEM: scanning electron microscopy; TEM: transmission electron microscopy.

| **Strain** | **Description** | **Phenotype during infection of *D. discoideum*** | **Experimental purpose** |
| --- | --- | --- | --- |
| wild-type (wt) | M strain | Virulent (reviewed in the main text) | CLEM (Gerstenmaier et al. 2015), CFUs counting (Kolonko et al. 2014), FACS (Lelong et al. 2011; Sattler, Monroy, and Soldati 2013; Kolonko et al. 2014; Gerstenmaier et al. 2015), IFA(Solomon, Leung, and Isberg 2003; Hagedorn and Soldati 2007; Cardenal-Munoz et al. 2017; Hagedorn et al. 2009; Lelong et al. 2011; Kolonko et al. 2014; Gerstenmaier et al. 2015), Immunoblot (Cardenal-Munoz et al. 2017), InfectChip (Delince et al. 2016), Live microscopy (Cardenal-Munoz et al. 2017; Barisch, Lopez-Jimenez, and Soldati 2015; Barisch and Soldati 2017; Kolonko et al. 2014; Gerstenmaier et al. 2015), Luminescence recording in microplate reader (Ouertatani-Sakouhi et al. 2017; Barisch et al. 2015; Barisch and Soldati 2017; Cardenal-Munoz et al. 2017), Plaque assay (Ouertatani-Sakouhi et al. 2017), qPCR (Cardenal-Munoz et al. 2017), SEM (Hagedorn et al. 2009), TEM (Hagedorn et al. 2009; Cardenal-Munoz et al. 2017; Barisch, Lopez-Jimenez, and Soldati 2015; Barisch and Soldati 2017; Gerstenmaier et al. 2015) |
| ∆CE | ESAT-6 and CFP-10 knockout (Gao et al. 2004) | No ubiquitination in *atg1*- cells (Cardenal-Munoz et al. 2017) | IFA (Cardenal-Munoz et al. 2017) |
| *mmar_2318*::Tn and ∆*2318* | *MMAR_2318* transposon and deletion mutants. This gene is involved in lipooligosaccharide (LOS) biosynthesis (Chen et al. 2015) | Attenuated (Chen et al. 2015), entry and intracellular replication like wt bacteria (Chen et al. 2015) | CFUs counting (Chen et al. 2015), Plaque assay (Chen et al. 2015) |
| *mmar_2319*::Tn and ∆*2319* | *MMAR_2319* transposon and deletion mutant. This gene is involved in LOS biosynthesis (Chen et al. 2015; van der Woude et al. 2012) | Attenuated (Chen et al. 2015), entry and intracellular replication like wt bacteria (Chen et al. 2015) | CFUs counting (Chen et al. 2015), Plaque assay (Chen et al. 2015) |
| *fadD28*::Tn | *fadD28* transposon mutant. FadD28 is an acyl coenzyme A synthase involved in the production of PDIMs and PGLs (Yu et al. 2012). | Attenuated (Ouertatani-Sakouhi et al. 2017) | Plaque assay (Ouertatani-Sakouhi et al. 2017) |
| L1D | Kan_r_ insertion in the *mag24-1* gene | Avirulant (Hagedorn and Soldati 2007; Alibaud et al. 2011), low intracellular proliferation (Solomon, Leung, and Isberg 2003; Hagedorn and Soldati 2007; Lelong et al. 2011), early exocytosis (Hagedorn and Soldati 2007), normal maturation of its phagosome (Hagedorn and Soldati 2007) | CFUs counting (Solomon, Leung, and Isberg 2003; Hagedorn and Soldati 2007), EM (Solomon, Leung, and Isberg 2003), FACS (Hagedorn and Soldati 2007; Lelong et al. 2011), IFA(Hagedorn and Soldati 2007; Kolonko et al. 2014), Plaque assay (Alibaud et al. 2011) |
| P59D | Kan_r_ insertion in the *mag24-2* gene, negative control for L1D (Solomon, Leung, and Isberg 2003) | Normal intracellular proliferation (Solomon, Leung, and Isberg 2003) | CFUs counting (Solomon, Leung, and Isberg 2003), EM (Solomon, Leung, and Isberg 2003) |
| ∆RD1 | *MM5446*-*MM5455* deletion mutant (Volkman et al. 2004) | Attenuated (Ouertatani-Sakouhi et al. 2017; Alibaud et al. 2011), normal GFP-Rab7a and VatB-RFP recruitment to early MCV (Cardenal-Munoz et al. 2017), normal LysoSensor Green fluorescence in early MCV (Cardenal-Munoz et al. 2017), transient increase of GFP-Rab11c recruitment to very early MCV (Cardenal-Munoz et al. 2017), inefficient downregulation of TORC1 activity during early infection (Cardenal-Munoz et al. 2017), decreased recruitment of Lamtor1-GFP, GFP-Rheb and GFP-Lst8 to early MCV (Cardenal-Munoz et al. 2017), inefficient MCV rupture and escape to cytosol (Cardenal-Munoz et al. 2017; Hagedorn et al. 2009; Barisch et al. 2015; Barisch and Soldati 2017), increased early MCV proteolytic activity (DQ Green BSA positive) (Cardenal-Munoz et al. 2017), inefficient increase of autophagy genes expression (Cardenal-Munoz et al. 2017), inefficient induction of autophagosome formation and blockage of autophagic flux (Cardenal-Munoz et al. 2017), decreased GFP-Atg8a and GFP-Ub recruitment (Cardenal-Munoz et al. 2017), decreased ubiquitination in *atg1*- cells (Cardenal-Munoz et al. 2017), decreased intracellular survival (Hagedorn et al. 2009), inefficient intracellular proliferation in wt and *atg1*- cells (Cardenal-Munoz et al. 2017; Hagedorn et al. 2009), increased autophagy-dependent bacterial killing (Cardenal-Munoz et al. 2017), rare clustering of Dgat2-GFP-LDs and targeting of RFP-Plin (Barisch and Soldati 2017; Barisch et al. 2015), complete labelling by Dgat2-GFP abolished (Barisch and Soldati 2017), absence of ejection (Hagedorn et al. 2009), normal ejection when co-infecting with wt *M. marinum* (Gerstenmaier et al. 2015) | FACS (Hagedorn et al. 2009), IFA (Hagedorn et al. 2009; Gerstenmaier et al. 2015), Immunoblot (Cardenal-Munoz et al. 2017), Live microscopy (Cardenal-Munoz et al. 2017; Barisch and Soldati 2017; Barisch et al. 2015), Luminescence recording in microplate reader (Cardenal-Munoz et al. 2017), Plaque assay (Ouertatani-Sakouhi et al. 2017; Alibaud et al. 2011), qPCR (Cardenal-Munoz et al. 2017), TEM (Hagedorn et al. 2009) |
| *tesA*::Tn | transposon insertion in *tesA* (*MMAR_1778)*. TesA is a type II thioesterase involved in the synthesis of major cell wall-associated lipids (Alibaud et al. 2011) | Attenuated (Alibaud et al. 2011; Ouertatani-Sakouhi et al. 2017) | Plaque assay (Alibaud et al. 2011; Ouertatani-Sakouhi et al. 2017) |

# Supplementary references

Alibaud, L., Y. Rombouts, X. Trivelli, A. Burguiere, S. L. Cirillo, J. D. Cirillo, J. F. Dubremetz, Y. Guerardel, G. Lutfalla, and L. Kremer. 2011. 'A Mycobacterium marinum TesA mutant defective for major cell wall-associated lipids is highly attenuated in Dictyostelium discoideum and zebrafish embryos', *Mol Microbiol*, 80: 919-34.

Barisch, C., A. T. Lopez-Jimenez, and T. Soldati. 2015. 'Live imaging of Mycobacterium marinum infection in Dictyostelium discoideum', *Methods Mol Biol*, 1285: 369-85.

Barisch, C., P. Paschke, M. Hagedorn, M. Maniak, and T. Soldati. 2015. 'Lipid droplet dynamics at early stages of Mycobacterium marinum infection in Dictyostelium', *Cell Microbiol*, 17: 1332-49.

Barisch, C., and T. Soldati. 2017. 'Mycobacterium marinum Degrades Both Triacylglycerols and Phospholipids from Its Dictyostelium Host to Synthesise Its Own Triacylglycerols and Generate Lipid Inclusions', *PLoS Pathog*, 13: e1006095.

Cardenal-Munoz, E., S. Arafah, A. T. Lopez-Jimenez, S. Kicka, A. Falaise, F. Bach, O. Schaad, J. S. King, M. Hagedorn, and T. Soldati. 2017. 'Mycobacterium marinum antagonistically induces an autophagic response while repressing the autophagic flux in a TORC1- and ESX-1-dependent manner', *PLoS Pathog*, 13: e1006344.

Chen, Y. Y., F. L. Yang, S. H. Wu, T. L. Lin, and J. T. Wang. 2015. 'Mycobacterium marinum mmar_2318 and mmar_2319 are Responsible for Lipooligosaccharide Biosynthesis and Virulence Toward Dictyostelium', *Front Microbiol*, 6: 1458.

Delince, M. J., J. B. Bureau, A. T. Lopez-Jimenez, P. Cosson, T. Soldati, and J. D. McKinney. 2016. 'A microfluidic cell-trapping device for single-cell tracking of host-microbe interactions', *Lab Chip*, 16: 3276-85.

Gao, L. Y., S. Guo, B. McLaughlin, H. Morisaki, J. N. Engel, and E. J. Brown. 2004. 'A mycobacterial virulence gene cluster extending RD1 is required for cytolysis, bacterial spreading and ESAT-6 secretion', *Mol Microbiol*, 53: 1677-93.

Gerstenmaier, L., R. Pilla, L. Herrmann, H. Herrmann, M. Prado, G. J. Villafano, M. Kolonko, R. Reimer, T. Soldati, J. S. King, and M. Hagedorn. 2015. 'The autophagic machinery ensures nonlytic transmission of mycobacteria', *Proc Natl Acad Sci U S A*, 112: E687-92.

Hagedorn, M., K. H. Rohde, D. G. Russell, and T. Soldati. 2009. 'Infection by tubercular mycobacteria is spread by nonlytic ejection from their amoeba hosts', *Science*, 323: 1729-33.

Hagedorn, M., and T. Soldati. 2007. 'Flotillin and RacH modulate the intracellular immunity of Dictyostelium to Mycobacterium marinum infection', *Cell Microbiol*, 9: 2716-33.

Kolonko, M., A. C. Geffken, T. Blumer, K. Hagens, U. E. Schaible, and M. Hagedorn. 2014. 'WASH-driven actin polymerization is required for efficient mycobacterial phagosome maturation arrest', *Cell Microbiol*, 16: 232-46.

Lelong, E., A. Marchetti, A. Gueho, W. C. Lima, N. Sattler, M. Molmeret, M. Hagedorn, T. Soldati, and P. Cosson. 2011. 'Role of magnesium and a phagosomal P-type ATPase in intracellular bacterial killing', *Cell Microbiol*, 13: 246-58.

Ouertatani-Sakouhi, H., S. Kicka, G. Chiriano, C. F. Harrison, H. Hilbi, L. Scapozza, T. Soldati, and P. Cosson. 2017. 'Inhibitors of Mycobacterium marinum virulence identified in a Dictyostelium discoideum host model', *PLoS One*, 12: e0181121.

Sattler, N., R. Monroy, and T. Soldati. 2013. 'Quantitative analysis of phagocytosis and phagosome maturation', *Methods Mol Biol*, 983: 383-402.

Solomon, J. M., G. S. Leung, and R. R. Isberg. 2003. 'Intracellular replication of Mycobacterium marinum within Dictyostelium discoideum: efficient replication in the absence of host coronin', *Infect Immun*, 71: 3578-86.

van der Woude, A. D., D. Sarkar, A. Bhatt, M. Sparrius, S. A. Raadsen, L. Boon, J. Geurtsen, A. M. van der Sar, J. Luirink, E. N. Houben, G. S. Besra, and W. Bitter. 2012. 'Unexpected link between lipooligosaccharide biosynthesis and surface protein release in Mycobacterium marinum', *J Biol Chem*, 287: 20417-29.

Volkman, H. E., H. Clay, D. Beery, J. C. Chang, D. R. Sherman, and L. Ramakrishnan. 2004. 'Tuberculous granuloma formation is enhanced by a mycobacterium virulence determinant', *PLoS Biol*, 2: e367.

Yu, J., V. Tran, M. Li, X. Huang, C. Niu, D. Wang, J. Zhu, J. Wang, Q. Gao, and J. Liu. 2012. 'Both phthiocerol dimycocerosates and phenolic glycolipids are required for virulence of Mycobacterium marinum', *Infect Immun*, 80: 1381-9.
