# Supplementary material for: When Dicty Met Myco, a (Not So) Romantic Story about One Amoeba and Its Intracellular Pathogen
Source: Front Cell Infect Microbiol. 2018 Jan 9;7:529. doi: 10.3389/fcimb.2017.00529 (PMC5767268; doi:10.3389/fcimb.2017.00529)
Supplement: Supplementary file 3 [file Table3.docx]

Supplementary Material

When Dicty met Myco, a (not so) Romantic Story about one Amoeba and its Intracellular Pathogen

Elena Cardenal-Muñoz^*^, Caroline Barisch, Louise Lefrançois, Ana Teresa López-Jiménez, Thierry Soldati

*** Correspondence:** Dr Elena Cardenal-Muñoz: elena.cardenal@unige.ch

# Supplementary Tables

**Supplementary Table 3.** ***D. discoideum* strains used to monitor *M. marinum* infection.**

| **Strain** | **Gene description** | ***M. marinum* behaviour / modulation of the infection** | **Experimental approach** |
| --- | --- | --- | --- |
| wild-type (wt) |  | *M. marinum* survives and replicates (reviewed in the main text) | CFUs counting (Solomon, Leung, and Isberg 2003; Hagedorn and Soldati 2007; Arafah et al. 2013; Chen et al. 2015; Kolonko et al. 2014), CLEM (Gerstenmaier et al. 2015), FACS (Hagedorn and Soldati 2007; Arafah et al. 2013; Hagedorn et al. 2009; Lelong et al. 2011; Sattler, Monroy, and Soldati 2013; Kolonko et al. 2014; Gerstenmaier et al. 2015), Fixed fluorescence microscopy (Hagedorn et al. 2009), IFA (Hagedorn and Soldati 2007; Hagedorn et al. 2009; Lelong et al. 2011; Kolonko et al. 2014; Gerstenmaier et al. 2015; Cardenal-Munoz et al. 2017), Immunoblot (Cardenal-Munoz et al. 2017), InfectChip (Delince et al. 2016), Live microscopy (Solomon, Leung, and Isberg 2003; Kolonko et al. 2014; Gerstenmaier et al. 2015; Cardenal-Munoz et al. 2017), Luminescence recording in microplate reader (Ouertatani-Sakouhi et al. 2017; Cardenal-Munoz et al. 2017), Plaque assay (Ouertatani-Sakouhi et al. 2017; Alibaud et al. 2011; Chen et al. 2015), pPCR (Cardenal-Munoz et al. 2017), SEM (Hagedorn et al. 2009), TEM (Solomon, Leung, and Isberg 2003; Hagedorn et al. 2009; Cardenal-Munoz et al. 2017; Gerstenmaier et al. 2015) |
| *atg1*- | Autophagy protein 1, homolog of the human ULK family, required for macroautophagy (Mesquita et al. 2015) | Early escape from MCV to cytosol (Cardenal-Munoz et al. 2017), increased wt bacteria ubiquitination (Cardenal-Munoz et al. 2017), inefficient ∆RD1 or ∆CE bacteria ubiquitination (Cardenal-Munoz et al. 2017), increased wt bacteria proliferation at late time points (Cardenal-Munoz et al. 2017), no effect on ∆RD1 proliferation (Cardenal-Munoz et al. 2017), normal formation of ejectosomes (Gerstenmaier et al. 2015), absence of phagophore at the distal pole of ejecting bacteria (Atg8a is absent and Ub is reduced) (Gerstenmaier et al. 2015), increased permeability during ejection leading to cell death (Gerstenmaier et al. 2015), deficient in intercellular dissemination (Gerstenmaier et al. 2015) | FACS (Gerstenmaier et al. 2015), IFA (Cardenal-Munoz et al. 2017; Gerstenmaier et al. 2015), Live microscopy (Cardenal-Munoz et al. 2017), Luminescence recording in microplate reader (Cardenal-Munoz et al. 2017), TEM (Cardenal-Munoz et al. 2017; Gerstenmaier et al. 2015) |
| *atg5*- | Autophagy protein 5, component of the ubiquitin-like conjugation machinery (Mesquita et al. 2016) | Atg8a absent from the distal pole of ejecting *M. marinum* (Gerstenmaier et al. 2015) | IFA (Gerstenmaier et al. 2015) |
| *atg6A/Beclin*- | Autophagy protein 6, homolog of human BECN1, component of the PI3K complex (Calvo-Garrido et al. 2010) | Reduced Atg8a recruitment to the distal pole of ejecting *M. marinum* (Gerstenmaier et al. 2015) | IFA (Gerstenmaier et al. 2015) |
| *atg7*- | Autophagy protein 7, component of the ubiquitin-like conjugation machinery (Mesquita et al. 2016) | Atg8a absent from the distal pole of ejecting *M. marinum* (Gerstenmaier et al. 2015) | IFA (Gerstenmaier et al. 2015) |
| *atg8a*- | Autophagy protein 8a, homolog of yeast Atg8 (dictyBase 2004) | Increased bacteria ubiquitination (Cardenal-Munoz et al. 2017), increased bacteria proliferation (Cardenal-Munoz et al. 2017) | IFA (Cardenal-Munoz et al. 2017), Luminescence recording in microplate reader (Cardenal-Munoz et al. 2017) |
| HG1569 (*corA*-) | Actin binding protein regulating actin nucleation (dictyBase 2004) | Lower bacteria uptake or early survival and proliferation (Solomon, Leung, and Isberg 2003) | CFUs counting (Solomon, Leung, and Isberg 2003) |
| *dgat1/2-* | Diacylglycerol transferases 1 and 2 | Accumulation of ILIs and morphology of bacteria and MCV like wt cells (Barisch and Soldati 2017) | High-content microscopy (Barisch and Soldati 2017), Luminescence recording in microplate reader (Barisch and Soldati 2017) |
| *kil2*- | Type V P-ATPase presumably involved in maintaining an optimal intraphagosomal magnesium concentration (Lelong et al. 2011) | *M. marinum* MCV and replication like in wt cells (Lelong et al. 2011) | IFA (Lelong et al. 2011) |
| *p62*/*sqstm1*- | Sequestosome-1, similar to the conserved p62 and ortholog of mammalian NBR1 (dictyBase 2004) | Normal recruitment of Atg8a to the distal pole of ejecting *M. marinum* (Gerstenmaier et al. 2015), increased bacteria proliferation (Cardenal-Munoz et al. 2017) | IFA (Gerstenmaier et al. 2015), Luminescence recording in microplate reader (Cardenal-Munoz et al. 2017) |
| *plnA-* | *D. discoideum* homologue of Perilipin previously named LSD1 (Barisch et al. 2015; Miura et al. 2002), structural LD protein (Du et al. 2013) | Inhibited bacterial growth starting from 20 hpi when *D. discoideum* is treated with FAs before infection (Barisch et al. 2015) | Luminescence recording in microplate reader (Barisch et al. 2015) |
| *racH*- | Rho GTPase regulating vesicle trafficking and acidification through regulation of actin polymerization (dictyBase 2004) | Normal bacterial escape from MCV to cytosol (Hagedorn et al. 2009), higher bacteria intracellular proliferation (Hagedorn and Soldati 2007), no bacteria egress/ejection (Hagedorn and Soldati 2007; Hagedorn et al. 2009), deficient in intercellular dissemination (Hagedorn et al. 2009) | CFUs counting (Hagedorn and Soldati 2007), FACS (Hagedorn and Soldati 2007; Hagedorn et al. 2009; Gerstenmaier et al. 2015), Fixed fluorescence microscopy (Hagedorn et al. 2009), IFA (Hagedorn and Soldati 2007; Hagedorn et al. 2009) |
| *vacA*- | Homolog of mammalian flotillin, expressed in post-lysosomes (dictyBase 2004) | Infection cycle like wt cells (Hagedorn and Soldati 2007; Hagedorn et al. 2009) | CFUs counting (Hagedorn and Soldati 2007), FACS (Hagedorn and Soldati 2007), IFA (Hagedorn and Soldati 2007; Hagedorn et al. 2009), |
| *vacB*- | Homolog of mammalian flotillin, marker of post-lysosomes(dictyBase 2004) | VatA accumulation in early MCV (Hagedorn and Soldati 2007), no net intracellular proliferation and low escape from MCV to cytosol (Hagedorn and Soldati 2007; Hagedorn et al. 2009), lower ejection rates (Hagedorn et al. 2009) | CFUs counting (Hagedorn and Soldati 2007), FACS (Hagedorn and Soldati 2007), IFA (Hagedorn and Soldati 2007; Hagedorn et al. 2009) |
| *wshA*- | WASH/WshA is involved in the recycling of the vATPase for maturation of lysosomes to post-lysosomes (Carnell et al. 2011) | F-actin does not associate with MCV (Kolonko et al. 2014), limited *M. marinum* growth (Kolonko et al. 2014), no ejection (Kolonko et al. 2014) | FACS (Kolonko et al. 2014), IFA (Kolonko et al. 2014) |

# Supplementary references

Alibaud, L., Y. Rombouts, X. Trivelli, A. Burguiere, S. L. Cirillo, J. D. Cirillo, J. F. Dubremetz, Y. Guerardel, G. Lutfalla, and L. Kremer. 2011. 'A Mycobacterium marinum TesA mutant defective for major cell wall-associated lipids is highly attenuated in Dictyostelium discoideum and zebrafish embryos', *Mol Microbiol*, 80: 919-34.

Arafah, S., S. Kicka, V. Trofimov, M. Hagedorn, N. Andreu, S. Wiles, B. Robertson, and T. Soldati. 2013. 'Setting up and monitoring an infection of Dictyostelium discoideum with mycobacteria', *Methods Mol Biol*, 983: 403-17.

Barisch, C., P. Paschke, M. Hagedorn, M. Maniak, and T. Soldati. 2015. 'Lipid droplet dynamics at early stages of Mycobacterium marinum infection in Dictyostelium', *Cell Microbiol*, 17: 1332-49.

Barisch, C., and T. Soldati. 2017. 'Mycobacterium marinum Degrades Both Triacylglycerols and Phospholipids from Its Dictyostelium Host to Synthesise Its Own Triacylglycerols and Generate Lipid Inclusions', *PLoS Pathog*, 13: e1006095.

Calvo-Garrido, J., S. Carilla-Latorre, Y. Kubohara, N. Santos-Rodrigo, A. Mesquita, T. Soldati, P. Golstein, and R. Escalante. 2010. 'Autophagy in Dictyostelium: genes and pathways, cell death and infection', *Autophagy*, 6: 686-701.

Cardenal-Munoz, E., S. Arafah, A. T. Lopez-Jimenez, S. Kicka, A. Falaise, F. Bach, O. Schaad, J. S. King, M. Hagedorn, and T. Soldati. 2017. 'Mycobacterium marinum antagonistically induces an autophagic response while repressing the autophagic flux in a TORC1- and ESX-1-dependent manner', *PLoS Pathog*, 13: e1006344.

Carnell, M., T. Zech, S. D. Calaminus, S. Ura, M. Hagedorn, S. A. Johnston, R. C. May, T. Soldati, L. M. Machesky, and R. H. Insall. 2011. 'Actin polymerization driven by WASH causes V-ATPase retrieval and vesicle neutralization before exocytosis', *J Cell Biol*, 193: 831-9.

Chen, Y. Y., F. L. Yang, S. H. Wu, T. L. Lin, and J. T. Wang. 2015. 'Mycobacterium marinum mmar_2318 and mmar_2319 are Responsible for Lipooligosaccharide Biosynthesis and Virulence Toward Dictyostelium', *Front Microbiol*, 6: 1458.

Delince, M. J., J. B. Bureau, A. T. Lopez-Jimenez, P. Cosson, T. Soldati, and J. D. McKinney. 2016. 'A microfluidic cell-trapping device for single-cell tracking of host-microbe interactions', *Lab Chip*, 16: 3276-85.

dictyBase. 2004. 'dictyBase'. [http://dictybase.org/](http://dictybase.org).

Du, X., C. Barisch, P. Paschke, C. Herrfurth, O. Bertinetti, N. Pawolleck, H. Otto, H. Ruhling, I. Feussner, F. W. Herberg, and M. Maniak. 2013. 'Dictyostelium lipid droplets host novel proteins', *Eukaryot Cell*, 12: 1517-29.

Gerstenmaier, L., R. Pilla, L. Herrmann, H. Herrmann, M. Prado, G. J. Villafano, M. Kolonko, R. Reimer, T. Soldati, J. S. King, and M. Hagedorn. 2015. 'The autophagic machinery ensures nonlytic transmission of mycobacteria', *Proc Natl Acad Sci U S A*, 112: E687-92.

Hagedorn, M., K. H. Rohde, D. G. Russell, and T. Soldati. 2009. 'Infection by tubercular mycobacteria is spread by nonlytic ejection from their amoeba hosts', *Science*, 323: 1729-33.

Hagedorn, M., and T. Soldati. 2007. 'Flotillin and RacH modulate the intracellular immunity of Dictyostelium to Mycobacterium marinum infection', *Cell Microbiol*, 9: 2716-33.

Kolonko, M., A. C. Geffken, T. Blumer, K. Hagens, U. E. Schaible, and M. Hagedorn. 2014. 'WASH-driven actin polymerization is required for efficient mycobacterial phagosome maturation arrest', *Cell Microbiol*, 16: 232-46.

Lelong, E., A. Marchetti, A. Gueho, W. C. Lima, N. Sattler, M. Molmeret, M. Hagedorn, T. Soldati, and P. Cosson. 2011. 'Role of magnesium and a phagosomal P-type ATPase in intracellular bacterial killing', *Cell Microbiol*, 13: 246-58.

Mesquita, A., E. Cardenal-Munoz, E. Dominguez, S. Munoz-Braceras, B. Nunez-Corcuera, B. A. Phillips, L. C. Tabara, Q. Xiong, R. Coria, L. Eichinger, P. Golstein, J. S. King, T. Soldati, O. Vincent, and R. Escalante. 2016. 'Autophagy in Dictyostelium: Mechanisms, regulation and disease in a simple biomedical model', *Autophagy*: 1-17.

Mesquita, A., L. C. Tabara, O. Martinez-Costa, N. Santos-Rodrigo, O. Vincent, and R. Escalante. 2015. 'Dissecting the function of Atg1 complex in Dictyostelium autophagy reveals a connection with the pentose phosphate pathway enzyme transketolase', *Open Biol*, 5.

Miura, S., J. W. Gan, J. Brzostowski, M. J. Parisi, C. J. Schultz, C. Londos, B. Oliver, and A. R. Kimmel. 2002. 'Functional conservation for lipid storage droplet association among Perilipin, ADRP, and TIP47 (PAT)-related proteins in mammals, Drosophila, and Dictyostelium', *J Biol Chem*, 277: 32253-7.

Ouertatani-Sakouhi, H., S. Kicka, G. Chiriano, C. F. Harrison, H. Hilbi, L. Scapozza, T. Soldati, and P. Cosson. 2017. 'Inhibitors of Mycobacterium marinum virulence identified in a Dictyostelium discoideum host model', *PLoS One*, 12: e0181121.

Sattler, N., R. Monroy, and T. Soldati. 2013. 'Quantitative analysis of phagocytosis and phagosome maturation', *Methods Mol Biol*, 983: 383-402.

Solomon, J. M., G. S. Leung, and R. R. Isberg. 2003. 'Intracellular replication of Mycobacterium marinum within Dictyostelium discoideum: efficient replication in the absence of host coronin', *Infect Immun*, 71: 3578-86.
