# Supplementary material for: When Dicty Met Myco, a (Not So) Romantic Story about One Amoeba and Its Intracellular Pathogen
Source: Front Cell Infect Microbiol. 2018 Jan 9;7:529. doi: 10.3389/fcimb.2017.00529 (PMC5767268; doi:10.3389/fcimb.2017.00529)
Supplement: Supplementary file 4 [file Table4.docx]

Supplementary Material

When Dicty met Myco, a (not so) Romantic Story about one Amoeba and its Intracellular Pathogen

Elena Cardenal-Muñoz^*^, Caroline Barisch, Louise Lefrançois, Ana Teresa López-Jiménez, Thierry Soldati

*** Correspondence:** Dr Elena Cardenal-Muñoz: elena.cardenal@unige.ch

# Supplementary Tables

**Supplementary Table 4.** ***D. discoideum* markers examined during *M. marinum* infection.** *AR-12/OSU-03012 is an autophagy inducer drug shown to decrease *M. marinum* load in *D. discoideum* (Cardenal-Munoz et al. 2017).

| **Marker** | **Description** | **Localisation of the marker in non-infected *D. discoideum*** | **Localisation of the marker during *M. marinum* infection** | **Experimental approach** |
| --- | --- | --- | --- | --- |
| GFP-2xFYVE | N-terminal GFP fused to the 2xFYVE domain, a specific PtdIns3P signalling lipid marker (Clarke et al. 2010) | The analogous RFP version localizes to PtIns3P-riched membranes, including omegasome-anchored phagophores (Calvo-Garrido et al. 2014) | Light localization to the distal pole of ejecting *M. marinum* (Gerstenmaier et al. 2015) | IFA (Gerstenmaier et al. 2015) |
| p-4E-BP1(T70) | 4E-BP1/FebA is the eukaryotic translation initiation factor 4E-binding protein 1. Similar to human EIF4EBP1 (Morio et al. 2001). Inactivated by phosphorylation by TOR (by homology with human). The anti-p-4E-BP1(T70) antibody can be used as TORC1 activity read out (Cardenal-Munoz et al. 2017; Rosel et al. 2012) | - | - | Immunoblot (Cardenal-Munoz et al. 2017) |
| GFP-ABD and ABD-GFP | N- or C-terminal GFP fused to the actin binding domain of filamin (Pang, Lee, and Knecht 1998) | Cytosol and F-actin (Pang, Lee, and Knecht 1998; Delince et al. 2016) | Cytosol and F-actin (Delince et al. 2016; Arafah et al. 2013; Gerstenmaier et al. 2015), phagocytic cup (Hagedorn et al. 2009), early MCV (Kolonko et al. 2014), ejectosome (Hagedorn et al. 2009; Gerstenmaier et al. 2015) | Fluorescence and Luminescence recording in microplate reader (Arafah et al. 2013), Live microscopy (Arafah et al. 2013; Hagedorn et al. 2009; Gerstenmaier et al. 2015), Fixed fluorescence microscopy (Hagedorn et al. 2009), IFA (Hagedorn et al. 2009), InfectChip (Delince et al. 2016) |
| Abp1 | F-actin binding protein. Used as loading control in immunoblot (Cardenal-Munoz et al. 2017) | Cytosol and cell cortex (Wang and O'Halloran 2006), phagosomes (Gopaldass et al. 2012) | - | Immunoblot (Cardenal-Munoz et al. 2017) |
| AmtA-mCherry/GFP | C-terminal mCherry/GFP fused to the ammonium transporter AmtA | Endo-lysosomes, phagosomes, ER membranes and nuclear envelope (Kirsten et al. 2008; Uchikawa, Yamamoto, and Inouye 2011) | MCV membrane (Barisch et al. 2015; Barisch and Soldati 2017) | Live microscopy (Barisch et al. 2015; Barisch and Soldati 2017) |
| Arp3 | Actin related protein 3, component of the Arp2/3 complex | Lamellipodia, filopodia and retraction fibers, cell-to-substrate adhesion region, phagocytic cup, macropinosome, cell poles during cytokinesis, post-lysosomes (Insall et al. 2001) | Absent from ejectosome (Hagedorn et al. 2009) | IFA (Hagedorn et al. 2009) |
| ArpC4-GFP | C-terminal GFP fused to ArpC4/ArcD, component of the Arp2/3 complex | Lysosomes and post-lysosomes (Thomason, King, and Insall 2017) | Transient presence in early MCV (Kolonko et al. 2014) | IFA (Kolonko et al. 2014) |
| *atg1* | Gene encoding the autophagy protein 1, homolog of the human ULK family, required for macroautophagy (Mesquita et al. 2015). Increased expression upon early ESX-1-dependent MCV damage (Cardenal-Munoz et al. 2017) | - | - | qPCR (Cardenal-Munoz et al. 2017) |
| Atg1-GFP | C-terminal GFP fused to Atg1 in pDXA-GFP. It complements the *atg1*- mutation (Cardenal-Munoz et al. 2017) | Cytosol (Tekinay et al. 2006) | - | IFA (Cardenal-Munoz et al. 2017) |
| Atg8a/Apg8 | Autophagy protein 8a, homolog of yeast Atg8 and ortholog of mammalian GABARAP subfamily (Mesquita et al. 2016). The *atg8a* expression increases upon early ESX-1-dependent MCV damage (Cardenal-Munoz et al. 2017) | - | Cytosol and recruited to damaged early MCV(Cardenal-Munoz et al. 2017), Patches along cytosolic bacteria (Gerstenmaier et al. 2015), Distal pole of ejecting *M. marinum* in wt but not *atg1*- cells (Gerstenmaier et al. 2015), Distal pole of ejecting *M. marinum* ∆RD1 during co-infection with *M. marinum* wt (Gerstenmaier et al. 2015) | IFA (Gerstenmaier et al. 2015; Cardenal-Munoz et al. 2017), qPCR (Cardenal-Munoz et al. 2017) |
| GFP-Atg8a | N-terminal GFP fused to Atg8a (Cardenal-Munoz et al. 2017) | Cytosol and autophagosomes (Cardenal-Munoz et al. 2017) | Decreased in cytosol and increased in autophagosomes (Cardenal-Munoz et al. 2017), recruited to damaged MCV (Cardenal-Munoz et al. 2017), increased recruitment as patches to *M. marinum* upon AR-12 treatment* (Cardenal-Munoz et al. 2017), increased recruitment to damaged MCV upon protease inhibitors treatment (Cardenal-Munoz et al. 2017) | Live microscopy (Cardenal-Munoz et al. 2017) |
| *atg8b* | Gene encoding the autophagy protein 8b, ortholog of mammalian LC3 subfamily (Mesquita et al. 2016). Increased expression upon early ESX-1-dependent MCV damage (Cardenal-Munoz et al. 2017) | - | - | qPCR (Cardenal-Munoz et al. 2017) |
| GFP-Atg18 | N-terminal GFP fused to autophagy protein 18, ortholog of mammalian WIPI2 (Mesquita et al. 2016) | Omegasome-anchored phagophores. It dissociates upon autophagosome completion [reviewed in (Mesquita et al. 2016)] | Phagophores (Cardenal-Munoz et al. 2017), recruited to damaged early MCV (Cardenal-Munoz et al. 2017), partially located to distal pole of ejecting *M. marinum* (Gerstenmaier et al. 2015) | IFA (Gerstenmaier et al. 2015), Live microscopy (Cardenal-Munoz et al. 2017) |
| Bodipy 493/503 | Neutral lipids dye | Host LDs after induction, cytosol when LDs are absent (Barisch et al. 2015) | Neutral lipids inside the MCV and bacterial ILIs depending on time after infection (Barisch et al. 2015) | Live microscopy (Barisch et al. 2015) |
| Bodipy 558/568 C12 | Fluorescently-labelled fatty acid | It incorporates into TAGs and stains LDs (Barisch et al. 2015; Barisch and Soldati 2017) | It incorporates into bacterial TAGs and ILIs (Barisch et al. 2015; Barisch and Soldati 2017) | Live microscopy (Barisch et al. 2015; Barisch and Soldati 2017), TLC (Barisch and Soldati 2017) |
| cathepsin D | Hydrolase | Lysosomes (Hagedorn and Soldati 2007) | Absent from early MCV (Hagedorn and Soldati 2007) | IFA (Hagedorn and Soldati 2007) |
| calmodulin | Calcium binding protein | Contractile vacuole (Zhu and Clarke 1992) | Absent from MCV (Kolonko et al. 2014) | IFA (Kolonko et al. 2014) |
| Coronin | CorA, actin binding protein | Phagocytic cup (Maniak et al. 1995), polar regions of mitotic cells (de Hostos et al. 1993), cell surface projections (de Hostos et al. 1991) | Ejectosome (Hagedorn et al. 2009) | IFA (Hagedorn et al. 2009) |
| Coronin-GFP | C-terminal GFP fused to the actin binding protein CorA | - | Interspersed around the MCV (Solomon, Leung, and Isberg 2003; Kolonko et al. 2014) | IFA (Solomon, Leung, and Isberg 2003; Kolonko et al. 2014) |
| DAPI | Membrane-impermeant DNA binding dye | Nuclei | Nuclei, absent in live amoeba during ejection (Hagedorn et al. 2009) | Live microscopy (Hagedorn et al. 2009) |
| Dgat2-GFP | C-terminal GFP fused to  the diacylglycerol transferase 2 | LDs (Du et al. 2014) | On LDs that first cluster at cytosol-exposed bacterial poles and then surrounds cytosolic bacteria (Barisch and Soldati 2017) | Live microscopy (Barisch and Soldati 2017), IFA (Barisch and Soldati 2017) |
| DQ Green BSA | Proteolysis sensor. Only fluorescent under proteolytic conditions | Proteolytic compartments such as mature phagosomes and lysosomes (Sattler, Monroy, and Soldati 2013; Bloomfield et al. 2015) | Devoid from damaged early MCV (Cardenal-Munoz et al. 2017) | Live microscopy (Cardenal-Munoz et al. 2017) |
| MB38::ESAT-6 | *M. marinum* ESAT-6 expressed under the Tet-Off system | - | - | FACS (Hagedorn et al. 2009), IFA (Hagedorn et al. 2009) |
| F-actin | Filamentous actin. Can be visualised by phalloidin staining with Alexa Fluor 488-phalloidin (green) (Gerstenmaier et al. 2015), Alexa Fluor 568-phalloidin (red) (Hagedorn et al. 2009; Kolonko et al. 2014; Gerstenmaier et al. 2015), Alexa Fluor 633-phalloidin (far-red) (Hagedorn et al. 2009) or Alexa Fluor 647-phalloidin (far-red) (Kolonko et al. 2014; Gerstenmaier et al. 2015), and with a Lifeact-GFP/RFP construct (Gerstenmaier et al. 2015) | Linear polymer microfilaments, cell cortex, lysosomes and post-lysosomes (Thomason, King, and Insall 2017) | Cell cortex (Kolonko et al. 2014; Gerstenmaier et al. 2015), phagocytic cup (Hagedorn et al. 2009), patchy at the early MCV (Kolonko et al. 2014), absent from VatA-positive early MCVs (Kolonko et al. 2014), absent from MCV during *M. marinum* proliferation phase (Kolonko et al. 2014), ejectosome (Hagedorn et al. 2009; Kolonko et al. 2014; Gerstenmaier et al. 2015), *M. marinum* ∆RD1ejectosome during co-infection with *M. marinum* wt (Gerstenmaier et al. 2015) | CLEM (Gerstenmaier et al. 2015), FACS (Gerstenmaier et al. 2015), IFA (Hagedorn et al. 2009; Kolonko et al. 2014; Gerstenmaier et al. 2015) |
| Filipin | Fluorescent dye staining non-esterified sterols | Sterols inside p80-positive endosomes and at the plasma membrane (Barisch et al. 2015) | MCV lumen (Barisch et al. 2015) | Fluorescence microscopy (Barisch et al. 2015) |
| GFP | Green fluorescent protein, negative control for GFP constructs | Cytosol (Myre et al. 2011) | Absent from distal pole of ejecting *M. marinum* (Gerstenmaier et al. 2015) | IFA (Gerstenmaier et al. 2015) |
| Lamtor1-GFP | C-terminal GFP fused to Lamtor1/p18, a member of the Ragulator complex (by similarity with human) (Cardenal-Munoz et al. 2017) | - | Accumulated in damaged early MCV (Cardenal-Munoz et al. 2017) | Live microscopy (Cardenal-Munoz et al. 2017) |
| GFP-Lst8 | N-terminal GFP fused to Lst8, component of the TORC1/2 complexes (Rosel et al. 2012) | - | Recruited to damaged early MCV (Cardenal-Munoz et al. 2017) | Live microscopy (Cardenal-Munoz et al. 2017) |
| LysoSensor Green DND-189 | Acidophilic dye. Only fluorescent under acidic conditions | Acidic compartments (Harris and Cardelli 2002; Neuhaus and Soldati 2000) | Slightly accumulated in early MCV (Cardenal-Munoz et al. 2017) | Live microscopy (Cardenal-Munoz et al. 2017) |
| Myosin IB | MyoB, motor protein binding actin | Lamellipodia, filopodia and cell-cell contact sites (Morita et al. 1996) | Ejectosome (Hagedorn et al. 2009) | IFA (Hagedorn et al. 2009) |
| Myosin II | MyoII/mhcA, motor protein binding actin | Posterior of the cell and in the cleavage furrow during cell migration and cytokinesis, respectively (Liang et al. 2002) | Absent from ejectosome (Hagedorn et al. 2009) | IFA (Hagedorn et al. 2009) |
| Neutral Red (NR) | Acidophilic dye | Acidic vesicles (Clarke and Maddera 2006) | Accumulated in early MCVs after LatA treatment (Kolonko et al. 2014) | Live microscopy (Kolonko et al. 2014) |
| *p62*/*sqstm1* | Gene encoding Sequestosome-1, a protein similar to the conserved p62 and ortholog of mammalian NBR1 (dictyBase 2004). Increased expression upon early ESX-1-dependent MCV damage (Cardenal-Munoz et al. 2017) | - | - | qPCR (Cardenal-Munoz et al. 2017) |
| GFP-p62/Sqstm1 | N-terminal GFP fused to p62 | - | Cytosol and recruited to damaged MCV(Cardenal-Munoz et al. 2017), patches along cytosolic bacteria (Gerstenmaier et al. 2015), distal pole of ejecting *M. marinum* (Gerstenmaier et al. 2015) | IFA (Gerstenmaier et al. 2015; Cardenal-Munoz et al. 2017) |
| p80 | Copper transporter | Ubiquitous in endocytic and phagocytic pathway (Hagedorn and Soldati 2007), plasma membrane (Hagedorn et al. 2009) | MCV membrane (Hagedorn and Soldati 2007; Kolonko et al. 2014; Cardenal-Munoz et al. 2017), present on a lower number of early MCVs in *wshA*- cells (Kolonko et al. 2014), reduced at early wt MCVs but not L1D MCVs upon LatA treatment (Kolonko et al. 2014), absent from cytosolic bacteria (Hagedorn et al. 2009; Cardenal-Munoz et al. 2017), at the outward plasma membrane deformation exerted by the ejecting bacterium and patchy on the ejected bacterium (Hagedorn et al. 2009) | IFA (Hagedorn and Soldati 2007; Hagedorn et al. 2009; Lelong et al. 2011; Kolonko et al. 2014; Cardenal-Munoz et al. 2017) |
| RFP/GFP-Plin | N-terminal RFP/GFP fused to Perilipin | Cytosol and on LDs surface upon induction (Barisch et al. 2015) | Co-localizes with cytosolic bacteria (Barisch et al. 2015) | Live microscopy (Barisch et al. 2015) |
| PM4C4 | Plasma membrane glycoprotein | Plasma membrane (Schwarz et al. 2000) | At the outward plasma membrane deformation exerted by the ejecting bacterium and patchy on the ejected bacterium (Hagedorn et al. 2009) | IFA (Hagedorn et al. 2009) |
| Propidium Iodide (PI) | Membrane impermeable DNA dye | It labels ded cells upon permeabilisation or lysis (Gerstenmaier et al. 2015) | Negligibly accumulated upon infection of wt cells (Gerstenmaier et al. 2015), highly accumulated upon infection of *atg1*- cells (Gerstenmaier et al. 2015) | FACS (Gerstenmaier et al. 2015), Fluorescence microscopy (Zhang et al. 2016) |
| GFP-Rab5a | N-terminal GFP fused to the Rab GTPase Rab5a | Early endosomes (by homology with human) | Transient presence after closure of the phagosome (Barisch, Lopez-Jimenez, and Soldati 2015) | Live microscopy (Barisch, Lopez-Jimenez, and Soldati 2015) |
| GFP-Rab7a | N-terminal GFP fused to the Rab GTPase Rab7a | Late endosomes and lysosomes (by homology with human) | Accumulated at MCV membrane (Barisch, Lopez-Jimenez, and Soldati 2015; Cardenal-Munoz et al. 2017) | Live microscopy (Barisch, Lopez-Jimenez, and Soldati 2015; Cardenal-Munoz et al. 2017) |
| GFP-Rab11c | N-terminal GFP fused to the Rab GTPase Rab11c. Rab11c is similar to human RAB11A. Presumably involved in phagophore elongation and autophagosome maturation (by homology with human RAB11) (Cardenal-Munoz et al. 2017) | Its RFP version localises on CV (Du et al. 2008), presumably in recycling endosomes (by homology with human) | Slightly present at early MCV (Cardenal-Munoz et al. 2017), accumulated at very early ∆RD1 MCV (Cardenal-Munoz et al. 2017) | Live microscopy (Cardenal-Munoz et al. 2017) |
| GFP-RacH | N-terminal GFP fused to RacH. It partially restores ejection in *racH*- cells (Hagedorn et al. 2009) | - | - | IFA (Hagedorn et al. 2009) |
| GFP-Raptor | N-terminal GFP fused to Raptor. Raptor is member and activator of the TORC1 complex (by similarity with yeasts and mammals) (Rosel et al. 2012) | - | Absent from early MCV (Cardenal-Munoz et al. 2017) | Live microscopy (Cardenal-Munoz et al. 2017) |
| p-Raptor(S863) | Anti-p-Raptor(S863) antibody, it can be used as TORC1 activity read out (Cardenal-Munoz et al. 2017; Rosel et al. 2012) | - | - | Immunoblot (Cardenal-Munoz et al. 2017) |
| GFP-Rheb | N-terminal GFP fused to the Ras GTPase Rheb | - | Recruited to damaged early MCV (Cardenal-Munoz et al. 2017) | Live microscopy (Cardenal-Munoz et al. 2017) |
| Topfluor-LysoPC | Fluorescently-labelled Lysophosphatidylcholine | It incorporates into phospholipids (Barisch and Soldati 2017) | The fluorescent label incorporates into bacterial TAGs and ILIs (Barisch and Soldati 2017) | Live microscopy (Barisch and Soldati 2017), TLC (Barisch and Soldati 2017) |
| TRITC-dextran | tetramethylrhodamine isothiocyanate (TRITC)-dextran. Red pH-insensitive fluid phase probe | Endolysosomes (Neuhaus, Almers, and Soldati 2002) | Present inside MCV (Gerstenmaier et al. 2015), absent from distal pole of ejecting *M. marinum* (Gerstenmaier et al. 2015) | Live microscopy (Gerstenmaier et al. 2015) |
| Ub (FK2) | Monoclonal antibody recognising mono- and polyubiquitinated conjugates | Cytosol (Cardenal-Munoz et al. 2017) | Cytosol and recruited to damaged MCV and cytosolic bacteria (Cardenal-Munoz et al. 2017; Gerstenmaier et al. 2015), increased localization to *M. marinum* in *atg1*- and *atg8*- cells (Cardenal-Munoz et al. 2017), distal pole of ejecting *M. marinum* (Gerstenmaier et al. 2015), reduced localization to ejecting *M. marinum* in *atg1*- cells (Gerstenmaier et al. 2015) | IFA (Cardenal-Munoz et al. 2017; Gerstenmaier et al. 2015) |
| GFP-Ub | N-terminal GFP fused to Ub (Cardenal-Munoz et al. 2017) | Cytosol | Cytosol and recruited to damaged MCV and cytosolic bacteria (Cardenal-Munoz et al. 2017) | IFA (Cardenal-Munoz et al. 2017), Live microscopy (Cardenal-Munoz et al. 2017) |
| vacuolins (VacA and VacB) | *D. discoideum* homologs of flotillin | Post-lysosomes (Hagedorn and Soldati 2007) | Absent from MCV during early infection (Hagedorn and Soldati 2007), accumulate on late MCV (Hagedorn et al. 2009), absent during ejection (Hagedorn et al. 2009) | IFA (Hagedorn and Soldati 2007; Hagedorn et al. 2009) |
| VacA-GFP | C-terminal GFP fused to  VacA | Post-lysosomes | Partially decorating the early MCV when overexpressed (Barisch, Lopez-Jimenez, and Soldati 2015) | Live microscopy (Barisch, Lopez-Jimenez, and Soldati 2015) |
| VatA | vATPase peripheral subunit | Contractile vacuole and lysosomes (Clarke et al. 2002) | Transient at early MCV (Hagedorn and Soldati 2007; Kolonko et al. 2014), absent from actin-positive early MCVs (Kolonko et al. 2014), present on a higher number of early MCVs in *wshA*- cells (Kolonko et al. 2014) | IFA (Hagedorn and Soldati 2007; Kolonko et al. 2014) |
| VatB-RFP | C-terminal RFP fused to VatB, a vATPase peripheral subunit | Lysosomes (Carnell et al. 2011), its GFP version localises on CV (Peracino et al. 2006) | early MCV (Cardenal-Munoz et al. 2017) | Live microscopy (Cardenal-Munoz et al. 2017) |
| VatM-GFP | C-terminal GFP fused to VatM, a transmembrane subunit of the vATPase | Contractile vacuole and lysosomes (Clarke et al. 2002) | Accumulated on early MCVs upon LatA treatment (Kolonko et al. 2014) | Live microscopy (Kolonko et al. 2014) |
| VMC (VacA-myc-cofilin) | Fusion construct between VacA and the actin-severing cofilin. It disrupts the actin coat on vacuolin-positive vesicles (Drengk et al. 2003) | Grouped endosomes without actin coat (Drengk et al. 2003) | Early MCV (Kolonko et al. 2014) | IFA (Kolonko et al. 2014) |
| GFP-WASH | N-terminal GFP fused to WASH/WshA. It partially restores ejection in *wshA*- cells (Kolonko et al. 2014) | Lysosomes and post-lysosomes (Thomason, King, and Insall 2017; Carnell et al. 2011) | Transient on early MCV (Kolonko et al. 2014) | IFA (Kolonko et al. 2014) |

# Supplementary references

Arafah, S., S. Kicka, V. Trofimov, M. Hagedorn, N. Andreu, S. Wiles, B. Robertson, and T. Soldati. 2013. 'Setting up and monitoring an infection of Dictyostelium discoideum with mycobacteria', *Methods Mol Biol*, 983: 403-17.

Barisch, C., A. T. Lopez-Jimenez, and T. Soldati. 2015. 'Live imaging of Mycobacterium marinum infection in Dictyostelium discoideum', *Methods Mol Biol*, 1285: 369-85.

Barisch, C., P. Paschke, M. Hagedorn, M. Maniak, and T. Soldati. 2015. 'Lipid droplet dynamics at early stages of Mycobacterium marinum infection in Dictyostelium', *Cell Microbiol*, 17: 1332-49.

Barisch, C., and T. Soldati. 2017. 'Mycobacterium marinum Degrades Both Triacylglycerols and Phospholipids from Its Dictyostelium Host to Synthesise Its Own Triacylglycerols and Generate Lipid Inclusions', *PLoS Pathog*, 13: e1006095.

Bloomfield, G., D. Traynor, S. P. Sander, D. M. Veltman, J. A. Pachebat, and R. R. Kay. 2015. 'Neurofibromin controls macropinocytosis and phagocytosis in Dictyostelium', *Elife*, 4.

Calvo-Garrido, J., J. S. King, S. Munoz-Braceras, and R. Escalante. 2014. 'Vmp1 regulates PtdIns3P signaling during autophagosome formation in Dictyostelium discoideum', *Traffic*, 15: 1235-46.

Cardenal-Munoz, E., S. Arafah, A. T. Lopez-Jimenez, S. Kicka, A. Falaise, F. Bach, O. Schaad, J. S. King, M. Hagedorn, and T. Soldati. 2017. 'Mycobacterium marinum antagonistically induces an autophagic response while repressing the autophagic flux in a TORC1- and ESX-1-dependent manner', *PLoS Pathog*, 13: e1006344.

Carnell, M., T. Zech, S. D. Calaminus, S. Ura, M. Hagedorn, S. A. Johnston, R. C. May, T. Soldati, L. M. Machesky, and R. H. Insall. 2011. 'Actin polymerization driven by WASH causes V-ATPase retrieval and vesicle neutralization before exocytosis', *J Cell Biol*, 193: 831-9.

Clarke, M., J. Kohler, Q. Arana, T. Liu, J. Heuser, and G. Gerisch. 2002. 'Dynamics of the vacuolar H(+)-ATPase in the contractile vacuole complex and the endosomal pathway of Dictyostelium cells', *J Cell Sci*, 115: 2893-905.

Clarke, M., and L. Maddera. 2006. 'Phagocyte meets prey: uptake, internalization, and killing of bacteria by Dictyostelium amoebae', *Eur J Cell Biol*, 85: 1001-10.

Clarke, M., L. Maddera, U. Engel, and G. Gerisch. 2010. 'Retrieval of the vacuolar H-ATPase from phagosomes revealed by live cell imaging', *PLoS One*, 5: e8585.

de Hostos, E. L., B. Bradtke, F. Lottspeich, R. Guggenheim, and G. Gerisch. 1991. 'Coronin, an actin binding protein of Dictyostelium discoideum localized to cell surface projections, has sequence similarities to G protein beta subunits', *EMBO J*, 10: 4097-104.

de Hostos, E. L., C. Rehfuess, B. Bradtke, D. R. Waddell, R. Albrecht, J. Murphy, and G. Gerisch. 1993. 'Dictyostelium mutants lacking the cytoskeletal protein coronin are defective in cytokinesis and cell motility', *J Cell Biol*, 120: 163-73.

Delince, M. J., J. B. Bureau, A. T. Lopez-Jimenez, P. Cosson, T. Soldati, and J. D. McKinney. 2016. 'A microfluidic cell-trapping device for single-cell tracking of host-microbe interactions', *Lab Chip*, 16: 3276-85.

dictyBase. 2004. 'dictyBase'. [http://dictybase.org/](http://dictybase.org).

Drengk, A., J. Fritsch, C. Schmauch, H. Ruhling, and M. Maniak. 2003. 'A coat of filamentous actin prevents clustering of late-endosomal vacuoles in vivo', *Curr Biol*, 13: 1814-9.

Du, F., K. Edwards, Z. Shen, B. Sun, A. De Lozanne, S. Briggs, and R. A. Firtel. 2008. 'Regulation of contractile vacuole formation and activity in Dictyostelium', *EMBO J*, 27: 2064-76.

Du, X., C. Herrfurth, T. Gottlieb, S. Kawelke, K. Feussner, H. Ruhling, I. Feussner, and M. Maniak. 2014. 'Dictyostelium discoideum Dgat2 can substitute for the essential function of Dgat1 in triglyceride production but not in ether lipid synthesis', *Eukaryot Cell*, 13: 517-26.

Gerstenmaier, L., R. Pilla, L. Herrmann, H. Herrmann, M. Prado, G. J. Villafano, M. Kolonko, R. Reimer, T. Soldati, J. S. King, and M. Hagedorn. 2015. 'The autophagic machinery ensures nonlytic transmission of mycobacteria', *Proc Natl Acad Sci U S A*, 112: E687-92.

Gopaldass, N., D. Patel, R. Kratzke, R. Dieckmann, S. Hausherr, M. Hagedorn, R. Monroy, J. Kruger, E. M. Neuhaus, E. Hoffmann, K. Hille, S. A. Kuznetsov, and T. Soldati. 2012. 'Dynamin A, Myosin IB and Abp1 couple phagosome maturation to F-actin binding', *Traffic*, 13: 120-30.

Hagedorn, M., K. H. Rohde, D. G. Russell, and T. Soldati. 2009. 'Infection by tubercular mycobacteria is spread by nonlytic ejection from their amoeba hosts', *Science*, 323: 1729-33.

Hagedorn, M., and T. Soldati. 2007. 'Flotillin and RacH modulate the intracellular immunity of Dictyostelium to Mycobacterium marinum infection', *Cell Microbiol*, 9: 2716-33.

Harris, E., and J. Cardelli. 2002. 'RabD, a Dictyostelium Rab14-related GTPase, regulates phagocytosis and homotypic phagosome and lysosome fusion', *J Cell Sci*, 115: 3703-13.

Insall, R., A. Muller-Taubenberger, L. Machesky, J. Kohler, E. Simmeth, S. J. Atkinson, I. Weber, and G. Gerisch. 2001. 'Dynamics of the Dictyostelium Arp2/3 complex in endocytosis, cytokinesis, and chemotaxis', *Cell Motil Cytoskeleton*, 50: 115-28.

Kirsten, J. H., Y. Xiong, C. T. Davis, and C. K. Singleton. 2008. 'Subcellular localization of ammonium transporters in Dictyostelium discoideum', *BMC Cell Biol*, 9: 71.

Kolonko, M., A. C. Geffken, T. Blumer, K. Hagens, U. E. Schaible, and M. Hagedorn. 2014. 'WASH-driven actin polymerization is required for efficient mycobacterial phagosome maturation arrest', *Cell Microbiol*, 16: 232-46.

Lelong, E., A. Marchetti, A. Gueho, W. C. Lima, N. Sattler, M. Molmeret, M. Hagedorn, T. Soldati, and P. Cosson. 2011. 'Role of magnesium and a phagosomal P-type ATPase in intracellular bacterial killing', *Cell Microbiol*, 13: 246-58.

Liang, W., L. Licate, H. Warrick, J. Spudich, and T. Egelhoff. 2002. 'Differential localization in cells of myosin II heavy chain kinases during cytokinesis and polarized migration', *BMC Cell Biol*, 3: 19.

Maniak, M., R. Rauchenberger, R. Albrecht, J. Murphy, and G. Gerisch. 1995. 'Coronin involved in phagocytosis: dynamics of particle-induced relocalization visualized by a green fluorescent protein Tag', *Cell*, 83: 915-24.

Mesquita, A., E. Cardenal-Munoz, E. Dominguez, S. Munoz-Braceras, B. Nunez-Corcuera, B. A. Phillips, L. C. Tabara, Q. Xiong, R. Coria, L. Eichinger, P. Golstein, J. S. King, T. Soldati, O. Vincent, and R. Escalante. 2016. 'Autophagy in Dictyostelium: Mechanisms, regulation and disease in a simple biomedical model', *Autophagy*: 1-17.

Mesquita, A., L. C. Tabara, O. Martinez-Costa, N. Santos-Rodrigo, O. Vincent, and R. Escalante. 2015. 'Dissecting the function of Atg1 complex in Dictyostelium autophagy reveals a connection with the pentose phosphate pathway enzyme transketolase', *Open Biol*, 5.

Morio, T., H. Yasukawa, H. Urushihara, T. Saito, H. Ochiai, I. Takeuchi, M. Maeda, and Y. Tanaka. 2001. 'FebA: a gene for eukaryotic translation initiation factor 4E-binding protein (4E-BP) in Dictyostelium discoideum', *Biochim Biophys Acta*, 1519: 65-9.

Morita, Y. S., G. Jung, J. A. Hammer, 3rd, and Y. Fukui. 1996. 'Localization of Dictyostelium myoB and myoD to filopodia and cell-cell contact sites using isoform-specific antibodies', *Eur J Cell Biol*, 71: 371-9.

Myre, M. A., A. L. Lumsden, M. N. Thompson, W. Wasco, M. E. MacDonald, and J. F. Gusella. 2011. 'Deficiency of huntingtin has pleiotropic effects in the social amoeba Dictyostelium discoideum', *PLoS Genet*, 7: e1002052.

Neuhaus, E. M., W. Almers, and T. Soldati. 2002. 'Morphology and dynamics of the endocytic pathway in Dictyostelium discoideum', *Mol Biol Cell*, 13: 1390-407.

Neuhaus, E. M., and T. Soldati. 2000. 'A myosin I is involved in membrane recycling from early endosomes', *J Cell Biol*, 150: 1013-26.

Pang, K. M., E. Lee, and D. A. Knecht. 1998. 'Use of a fusion protein between GFP and an actin-binding domain to visualize transient filamentous-actin structures', *Curr Biol*, 8: 405-8.

Peracino, B., C. Wagner, A. Balest, A. Balbo, B. Pergolizzi, A. A. Noegel, M. Steinert, and S. Bozzaro. 2006. 'Function and mechanism of action of Dictyostelium Nramp1 (Slc11a1) in bacterial infection', *Traffic*, 7: 22-38.

Rosel, D., T. Khurana, A. Majithia, X. Huang, R. Bhandari, and A. R. Kimmel. 2012. 'TOR complex 2 (TORC2) in Dictyostelium suppresses phagocytic nutrient capture independently of TORC1-mediated nutrient sensing', *J Cell Sci*, 125: 37-48.

Sattler, N., R. Monroy, and T. Soldati. 2013. 'Quantitative analysis of phagocytosis and phagosome maturation', *Methods Mol Biol*, 983: 383-402.

Schwarz, E. C., E. M. Neuhaus, C. Kistler, A. W. Henkel, and T. Soldati. 2000. 'Dictyostelium myosin IK is involved in the maintenance of cortical tension and affects motility and phagocytosis', *J Cell Sci*, 113 ( Pt 4): 621-33.

Solomon, J. M., G. S. Leung, and R. R. Isberg. 2003. 'Intracellular replication of Mycobacterium marinum within Dictyostelium discoideum: efficient replication in the absence of host coronin', *Infect Immun*, 71: 3578-86.

Tekinay, T., M. Y. Wu, G. P. Otto, O. R. Anderson, and R. H. Kessin. 2006. 'Function of the Dictyostelium discoideum Atg1 kinase during autophagy and development', *Eukaryot Cell*, 5: 1797-806.

Thomason, P. A., J. S. King, and R. H. Insall. 2017. 'Mroh1, a lysosomal regulator localized by WASH-generated actin', *J Cell Sci*, 130: 1785-95.

Uchikawa, T., A. Yamamoto, and K. Inouye. 2011. 'Origin and function of the stalk-cell vacuole in Dictyostelium', *Dev Biol*, 352: 48-57.

Wang, Y., and T. J. O'Halloran. 2006. 'Abp1 regulates pseudopodium number in chemotaxing Dictyostelium cells', *J Cell Sci*, 119: 702-10.

Zhang, X., O. Zhuchenko, A. Kuspa, and T. Soldati. 2016. 'Social amoebae trap and kill bacteria by casting DNA nets', *Nat Commun*, 7: 10938.

Zhu, Q., and M. Clarke. 1992. 'Association of calmodulin and an unconventional myosin with the contractile vacuole complex of Dictyostelium discoideum', *J Cell Biol*, 118: 347-58.
